# Metal-free pyridinium salts with strong room-temperature phosphorescence and microsecond radiative lifetime

**DOI:** 10.1039/d5sc03813h

**Published:** 2025-08-15

**Authors:** Eetu Hakkarainen, Hao-Cheng Lin, Anton A. Nechaev, Vsevolod A. Peshkov, Toni Eskelinen, Kai-Hsin Chang, Tzu-Hao Liao, Po-Yu Chen, Igor O. Koshevoy, Hao-Wu Lin, Pi-Tai Chou, Andrey Belyaev

**Affiliations:** a Department of Chemistry and Sustainable Technology, University of Eastern Finland Yliopistokatu 7 80101 Joensuu Finland andrei.beliaev@uef.fi; b Department of Materials Science and Engineering, National Tsing-Hua University 101, Sec. 2, Kuang-Fu Road Hsinchu 30013 Taiwan Republic of China hwlin@mx.nthu.edu.tw; c Department of Chemistry, University of Jyväskylä Survontie 9 B 40500 Jyväskylä Finland anton.a.nechaev@jyu.fi; d Department of Chemistry and Materials Science, Aalto University FI-00076 Aalto Finland; e Department of Chemistry, National Taiwan University Taipei 10617 Taiwan Republic of China chop@ntu.edu.tw; f Advanced Packaging Instrumentation and Metrology Laoratory, Industrial Technology Research Institute Hsinchu 30013 Taiwan Republic of China

## Abstract

Easily processed metal-free phosphorescent luminophores with a fast rate of phosphorescence are emerging as promising materials for advanced optoelectronics. Alkylation of a modified vitamin B6 vitamer (pyridoxine) affords a family of pyridinium-derived ionic pairs 1–7 exhibiting variable anion-π interactions in the solid state. Such a noncovalent cation–anion network promotes tunable room-temperature phosphorescence (RTP, *λ*_em_ = 510–565 nm) in crystalline materials stemming from anion(I^−^)-π(pyridinium^+^) charge transfer. Systematic X-ray structural and computational studies manifest the key role of the anion(I^−^)-π(pyridinium^+^) distance in the spin–orbit coupling, hence the observed RTP. For the studied pyridinium salts with RTP, the radiative rate constants (*k*_r_) reach up to 0.9–1.3 × 10^5^ s^−1^ which are competitive with those of many noble metal emitters. Ion pair 2 reached an RTP with a quantum yield of 93% and was successfully demonstrated as an excellent X-ray scintillating dye in neat films. The demonstrated strategy of attaining intense RTP in small metal-free accessible molecules, *i.e.*, atom-photon economy, represents a new twist in designing efficient and sustainable photofunctional molecular materials.

## Abstract 2

A new frontier of easily processed metal-free phosphorescent chromophores: tuning of anion(I^−^)-π^+^ interactions in pyridinium-based ionic pairs unlocks efficient and rapid charge-transfer phosphorescence with radiative rates of 0.9–1.3 × 10^5^ s^−1^.

## Graphical abstract

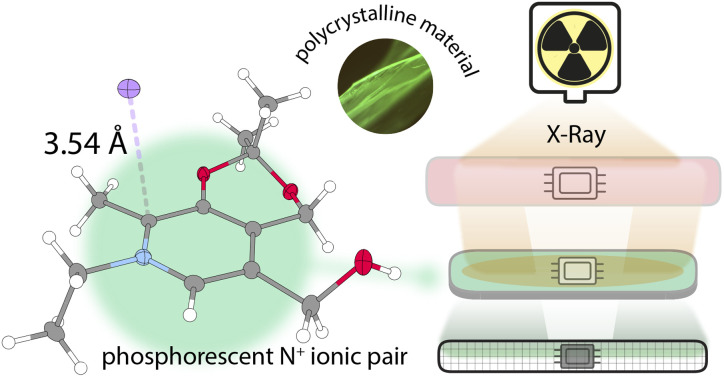

## Introduction

Luminescent materials, capable of harnessing a triplet excited state (T_*n*_) following light absorption or charge recombination, are critical for achieving theoretical efficiency maxima.^1^ The wide use of such luminophores includes the areas of OLED displays and photovoltaics and advanced solutions for photonic applications in sensing, (bio)imaging, X-ray diagnostics, photocatalysis, and anti-counterfeiting systems.^2–8^ Exploiting triplet states for radiative processes is typically associated with late transition metal complexes showing room temperature phosphorescence (RTP) or thermally activated delayed fluorescence (TADF).^9–12^ Apart from specific molecular design, this capability arises from the intrinsic strong spin–orbit coupling (SOC) of a metal center, which facilitates intersystem crossing (ISC) from the singlet (S_1_) to the triplet T_*n*_ states and subsequent relaxation to the T_1_ state, followed by T_1_ → S_0_ nonradiative or radiative decay. The radiative decay rate constant (*k*_r_ = Q.Y/*t*, where Q.Y. = quantum yield) of late transition metal complexes can be as high as 10^6^ s^−1^, crucial for minimizing bleaching due to hot reactions and radical formation.

In contrast, the majority of purely organic molecules suffer from small SOC, causing two main adverse effects: (i) the smaller SOC, hence the slow ISC, limits the efficiency of T_1_ population; (ii) the resulting small mixing of S_1_ and T_1_ states gives a small T_1_ → S_0_*k*_r_ value, hence the dominant nonradiative decay. Manipulation with SOC/ISC to achieve relatively high T_1_ population and *k*_r_ values for organic molecules under ambient conditions is possible through targeted molecular construction and materials fabrication strategies. For instance, stereochemical control of molecular fragments and their interconnectivity in donor–acceptor systems represents a promising strategy for attaining the required photophysical properties.^13–16^ Significant spin-vibronic coupling, combined with the presence of heteroatoms such as B, N, S, O, or P, enhances state mixing between the S_*n*_ and T_*n*_ states, thereby accelerating ISC.^17,18^ Recent progress in the rational design of RTP materials has been achieved by employing co-crystallization, H-aggregation, halogen bonding, and the non-metal heavy atom effect.^19–25^ In the latter case, structural modifications can tune the ISC rate, as SOC is proportional to *Z*^*4*^*/r*^*3*^ (where *Z* is the atomic number and *r* is the distance between the heavy atom and the center of a chromophore). Appending covalently bound heavy atoms to emissive molecules enables (ultra)long organic phosphorescence with relatively slow rates (*k*_r_) of 10^0−4^ s^−1^. While such properties are advantageous for applications in photodynamic therapy and photocatalysis,^26,27^ the development of precious metal-free luminophores capable of harvesting triplet excitons with *k*_r_ in the order of 10^6^–10^7^ s^−1^, *i.e.*, comparable to fast transition metal phosphors, remains a critical challenge. In this context, contact ion pairs (CIPs) have emerged as a promising avenue.^28^ Recent studies demonstrate that CIPs manifesting short distances between heavy anions (Br and I) and ‘onium’ chromophores, such as tetraphenyloxazolium,^29^ quinolinium,^30,31^ arylene diimides,^32^ benzophospholiums^33^ or mono-^34–38^ and diphosphonium,^39,40^ can achieve efficient solid-state RTP or TADF (for more details see Table S1). In these species, SOC is promoted by the external heavy atom effect and the anion → π charge-transfer (CT) configuration of the triplet excited state.

Herein, we make a significant contribution by presenting a new family of CIPs: pyridinium salts derived from pyridoxine (PN), a vitamer of Vitamin B6, with a nearly quantitative yield. Together with excellent RTP intensity, these efficient luminophores align well with the principles of atom-photon economy, *i.e.*, a sustainable strategy that optimizes overall energy utilization by harmonizing synthetic assessment and atomic structures with the photon-releasing process, maximizing light energy conversion and minimizing inefficiencies at both levels.

## Results and discussion

**Fig. 1:**
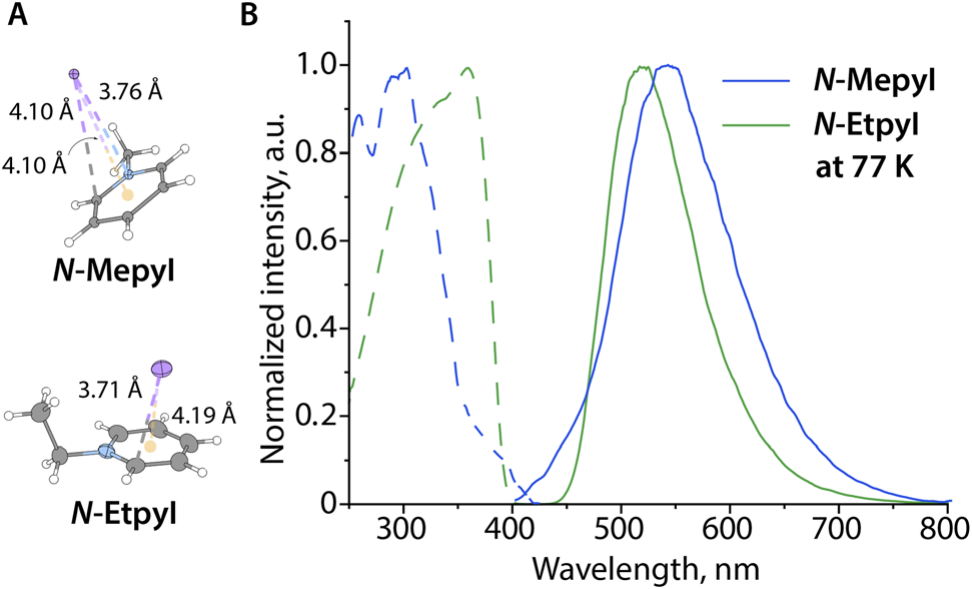
(A) Molecular structures of ion pairs *N*-MepyI (ball and stick representation) and *N*-EtpyI (displacement ellipsoids are shown at the 50% probability level) with depicted shortest anion-π interactions, and (B) their excitation (dashed) and emission (solid) spectra measured from solids at 77 K.

Short anion-π contacts, which are close to the sum of the van der Waals radii, are a prerequisite for realizing the CT states (*i.e.*, 3.53–3.68 Å for N or C atoms, respectively, see Table S2 for selected results of the Cambridge Structural Database search). These structural conditions were found only in a fraction of the pyridinium and quinolinium iodides. The simplest derivatives, *N*-methyl-(*N*-MepyI) and *N*-ethyl pyridinium iodides (*N*-EtpyI), show phosphorescence of a CT origin only at low temperature, probably as a result of insufficiently close contact (Fig. 1). The shortest I⋯N or I⋯C distances (I^…^centroid distances) fall in the range of 3.76/4.10 Å (4.10/5.12 Å) for *N*-MepyI and 3.70/4.03 Å (4.19/4.85 Å) for *N*-EtpyI (Fig. S1), implying small SOC and slow ISC, overall unable to compete with fast rates of non-radiative transitions.

**Fig. 2:**
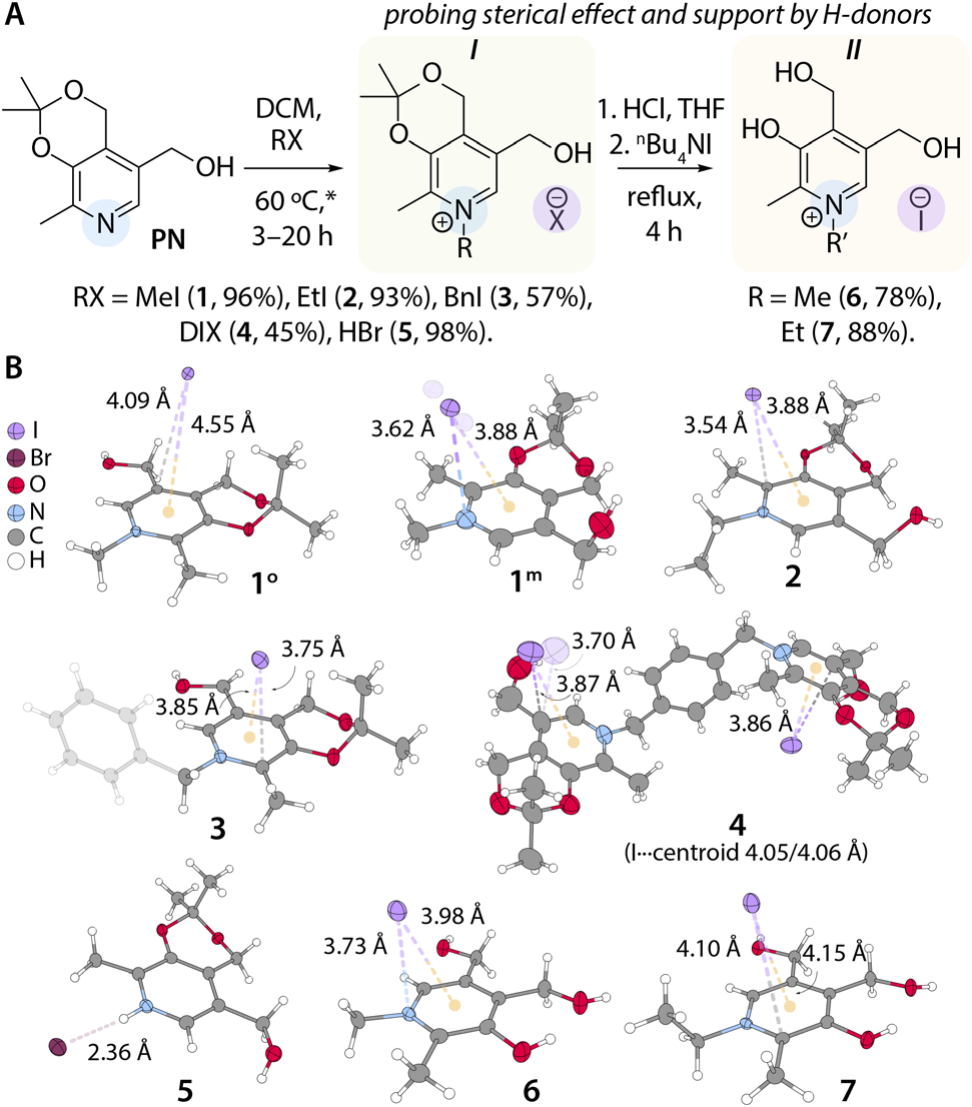
(A) Synthesis of pyridinium salts 1–7; * in a sealed tube and (B) molecular structures of ion pairs 1–7 (displacement ellipsoids are shown at the 50% probability level) with depicted shortest I⋯C, I⋯N, and I⋯centroid distances.

Since the strength of ionic pairing is likely governed by a combination of steric and electronic effects inherent to the ionic core and can be regulated by directing groups,^41,42^ optimal structural and electronic adjustments are thus of primary importance in achieving desired phosphorescence under ambient conditions. For this purpose, the B6 vitamer PN (Fig. 2A) was chosen as an up-and-coming candidate for the preparation of ionic crystalline materials, in which the packing of constituents can be controlled by introducing sterically different groups. Furthermore, the availability of hydroxyl (OH) groups attached to a pyridine core provides additional degrees of freedom for crystal engineering, as these groups can form hydrogen bonding with halide anions and solvent molecules that affect the overall robustness, stability, and optical characteristics of the system.

The pyridinium salts 1–5, forming group *I* with an ether (dioxino) fragment (Fig. 2A), were prepared *via* direct one-step alkylation (protonation in the case of 5) of isopropylidene pyridoxine. Compounds in group II, in which the pyridinium core bears three hydroxyl groups, were obtained *via* acidic ether cleavage of 1 and 2, followed by anion metathesis using ^*n*^Bu_4_NI. After multiple recrystallization cycles, the titled salts were isolated as uniform white or pale-yellow crystalline solids in moderate to excellent yields (see the SI). Thermal analysis (TGA and DSC) revealed that the samples exhibited limited thermal stability, decomposing after melting points, which range from 385 to 450 K (Fig. S2). The methylated derivatives display significantly higher values than their ethylated analogues. The composition and purity of the synthesized compounds were confirmed by nuclear magnetic resonance (NMR) spectroscopy, high-resolution mass spectrometry (HR-MS), and high-performance liquid chromatography (HPLC) (Fig. S3, see the SI).

All obtained species were characterized by single-crystal X-ray diffraction (scXRD, see the SI). The I^−^–pyridinium^+^ contacts range from 3.54 to 4.10 Å in alkylated compounds 1–4, 6, and 7 (Fig. 2B, S4–7 and Tables S3–S6) and are consistent with the data for corresponding anion–π interactions.^43,44^ Depending on crystallization conditions, the methylated derivative yielded two non-solvated polymorphs: 1° (orthorhombic, *Pbca* space group) and 1^m^ (monoclinic, *P*2_1_/*c* space group). In both packings, iodide positioning is primarily dictated by a network of C–H⋯I interactions. Thus, a smaller involvement of the I^−^ in hydrogen bonding in 1^m^ leads to a significantly shorter anion-π^+^ distance than that in 1° (η^1^-type, 3.62 Å *vs. η*^2^, 4.09 Å, Fig. 2B and S4). In salt 2, the iodide sits directly above the plane of the ring, exhibiting the shortest I⋯C distance (*η*^2^, 3.54/3.67 Å) among known emissive CIPs (Table S1), likely due to the weaker C–H acidity of the N-bound alkyl (Et) group. The synergy between steric and electrostatic effects promotes tight ion pairing *via* favorable non-covalent crystal forces. In contrast, crystallites 3 and 4, featuring bulky benzyl and xylyl groups, show increased anion–cation separation (3.75–3.86 Å) due to additional intramolecular π–π stacking and multiple C^bn/xyl^-H⋯I interactions. A combination of reduced steric hindrance and hydrogen donors (OH groups) in the pyridinium core within group *II* also affects the anion-π cation bonding by a systematic increase of the distances in salts 6 (3.73/3.78 Å) and 7 (3.93/4.11 Å) compared to 1^m^ and 2. The lower π-acidity of 6 and 7 (see calculated electronic potential surfaces, Fig. S5), along with enhanced hydrogen bonding, leads to the formation of intermolecular dimers, which further stabilize cations (see Fig. S6). Iodide-centroid separations for crystalline 1–4, 6, and 7 follow a similar trend and are found in the range of 3.85–4.66 Å, with 1^m^, 2, 3, and 6 demonstrating the shortest distances of 3.85–3.98 Å. Variable-temperature (VT) scXRD analyses of both 2 and 6 reveal positive thermal expansion of the unit cell, accompanied by the elongation of the anion–π and I⋯centroid contacts (Fig. S7 and S8).

The powder X-ray diffractograms (PXRDs) match simulated patterns for 1^m^, 1°, 2, and 4–7, confirming phase purity, whereas 3 becomes amorphous after losing crystallized dichloromethane (Fig. S9).

The reflectance spectra of the solid samples show the low-lying energy absorption shoulders extending up to 500 nm (Fig. S10). They originate from charge transfer anion–π states,^32,40,45^ which are confirmed by theoretically predicted transitions S_0_ → S_1_ and charge-density plots depicted in Fig. S11, S12 and 3C.

Upon photoexcitation, the behavior of solids 1°, 3, 4, and 7 correlates with the data obtained for *N*-MepyI and *N*-EtpyI (Fig. S1 and Table S7), which are emissive only under cryogenic conditions (Fig. S13 and Table S7). Note that all these salts appear to lack short-range anion (I^−^)–π distances (Table S7) and thus show insufficient intermolecular interactions. The corresponding emission presumably originates from either a triplet charge-transfer state (^3^CT, anion–π) or a mixed ^3^CT and a triplet locally excited state (^3^LE, ππ*). For instance, the asymmetric emission profiles of 1° and 7 are found in the blue region (*λ*_em_ = 440 nm for 1° and 480 nm for 7), while the variations of lifetimes monitored at different wavelengths are consistent with the proposed assignment. Compounds 3 and 4 with relatively short anion–cation distances display fast decay dynamics that are devoid of longer-lived components (*cf.* ∼10^2^ to 10^3^ μs found for 1° and 7, Table S7), indicative of a more efficient ^3^CT (anion(I^−^)–π) relaxation pathway. Time-dependent density functional (TDDFT) studies reveal that 1° and 7 comprise low-lying T_1_ and T_2_ states having mainly the CT and π–π* configurations, respectively, whereas for 3 these states hold a CT character (Fig. S11 and Table S9).

The packing of 5 is stabilized solely by in-plane C–H⋯Br hydrogen bonding (2.36 Å), and its solid remains non-emissive even upon cooling to 77 K.

**Fig. 3:**
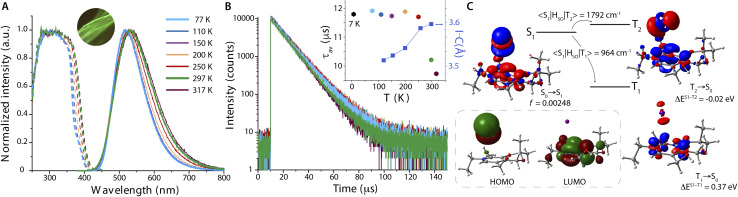
(A) Variable temperature (VT) excitation (dashed), emission (solid), and (B) time-resolved decays of crystalline 2 (inset shows VT *τ*_av_ and I⋯C distance of 2). (C) Plots of HOMO/LUMO; excitation (S_0_ → S_1_) and triplet emission (T_2_, T_1_ → S_0_) electron density difference plots for 2 (loss of electron density: blue and gain: red).

In contrast, the photoluminescence of 1^m^, 2, and 6 in the solid state is observed at room temperature (Tables 1, S8, Fig. 3 and S14), with Q.Y. reaching 7%, 93%, and 3%, respectively. Broad, structureless emission bands in green (*λ*_em_ = 525 nm for 1^m^ and 532 nm for 2) and yellow regions (*λ*_em_ = 565 nm for 6) are characterized by the average lifetimes in the microsecond domain (Table 1) and are assigned to the ^3^CT (anion–π) excited state, *i.e.* phosphorescence. The enhanced SOC and ISC could be related to pronounced anion-π interactions, which correlate with short contacts determined by scRXD in the ground state (*vide supra*). Importantly, both the phosphorescence intensity and excited-state lifetime under aerobic conditions are quenched by molecular oxygen by a modest 2–5% for 1^m^, 2, and 6 (Fig. S14). This slight decrease indicates limited accessibility of quenching oxygen to the triplet chromophore core, likely due to the tight molecular packing in the solid state.

**Table 1:** Photophysical summary of the pyridinium salts 1^m^, 2, and 6

	*λ*_exc_ [nm]	*λ*_em_ [nm]	*τ*_av_ a [μs]	QY	*k*_*r*_ b [×10^4^ s^−1^]
1^m^, RT	330	525	0.53	0.07	13.2
77 K	335	478	13.23^c^		
2, RT	365	532	10.21	0.93	9.1
77 K	350, 380	520	11.91		8.4^d^
7 K	350	520	11.79		8.5^d^
6, RT	330	565	2.75	0.03	1.1
77 K	320, 375	475 and 575	104.81^e^/25.02^f^		

a The average amplitude-weighted emission lifetime for multiexponential decays (*τ*_av_ = Σ*A*_i_*t*_i_, where *A*_i_ – the weight of the exponent).

b *k*_r_ = QY/*τ*_av_.

c Monitored at 480 nm.

d Calculated with the assumption of a QY of 1.0.

e 475 nm.

f 620 nm.

VT measurements were then carried out for solid salts 1^m^, 2, and 6. At 77 K, the excited states of 1^m^ and 6 shift from predominantly ^3^CT to a mixed ^3^CT/^3^LE character, as evidenced by hypsochromic shifts of the emission maxima to 478 (1^m^) and 475 nm (6) (Fig. S15). The LE contribution is particularly visible for 6 as the long-lived lifetime components become dominant when excited state decays are monitored at 470–480 nm (77 and 150 K). On the other hand, the presence of at least two thermally non-equilibrated states in 1^m^ is evidenced by two factors: (i) an average lifetime collected at 130 K, which shows an almost 20-fold increase compared to that at 298 K and (ii) variable lifetimes monitored at different wavelengths at 77 K, *i.e.*, *τ*_av_^@440^ = 10.75 μs; *τ*_av_^@480^ = 13.23 μs; *τ*_av_^@550^ = 12.24 μs (Fig. S15 and Table S8). The proposed emission mechanism, *i.e.* mixing of ^3^CT and ^3^LE configurations, aligns well with TDDFT analysis, as the predicted T_1_ and T_2_ states for 6 show anion–π and π–π* characters, respectively, and 1^m^ exhibits CT with a noticeable π–π* admixture in both T_1_/T_2_ states (Fig. S12).

Ion-pair 2 exhibits a moderate hypsochromic shift (532 → 520 nm, 540 cm^−1^) and narrowing of the band (ΔFWHM = 643 cm^−1^) upon cooling to 7 K, likely due to a rigidochromic effect (Fig. 3A).^46^ The VT scXRD data for 2 (Fig. 3B inset and S8) reveal a nearly 2% contraction of anion–π distance from 3.61 Å at 291 K to 3.54 Å at 120 K, which contributes to an increased optical bandgap and enhances charge-transfer (CT) character. As a result, the excited-state dynamics remain unchanged across the 250–7 K temperature range, indicating negligible radiationless relaxation and a plateau in the radiative decay rate (*k*_*r*_ ≈ 8.5 × 10^4^ s^−1^, Fig. 3B, S16 and Table S8) that rules out the TADF mechanism. The TDDFT data support the decisive role of anion–π^+^ CT in the excited-state dynamics for 2. This plausibly involves fast intersystem crossing from S_1_ to a nearly energetically degenerate T_2_ state (Δ*E*(S_1_–T_2_) = −0.02 eV and Table S9), followed by internal conversion to the T_1_ state and T_1_ → S_0_ phosphorescence (Fig. 3C). The SOC matrix element (SOCME) between S_1_ and T_2_ states (ξ(S_1_, T_2_) = 1792 cm^−1^) is almost twice as large as the corresponding value for S_1_ and T_1_ states (ξ(S_1_, T_1_) = 964 cm^−1^); thus, S_1_ → T_2_ is a favorable path for spin–orbit coupling. Nevertheless, the direct population of T_1_ by ISC from the S_1_ cannot be excluded. The analysis of orbital configurations of S_1_ and T_1_/T_2_ states reveals their hybrid nature with substantial n–π* and π–π* contributions, leading to significant SOC and fast ISC that is consistent with El-Sayed's rules.^47^ Importantly, the conservation of orbital angular momentum required for SOC is provided by the change of orientation of the p orbital of the iodide involved in the electronic transition, *e.g.* from p_*z*_ in S_1_ to p_*x*_/p_*y*_ in T_2_. This formal orthogonal orbital flipping maximizes the angular momentum (*L*), increasing the magnitude of SOC < S_1_|*H*_SO_|*T*_*n*_>:
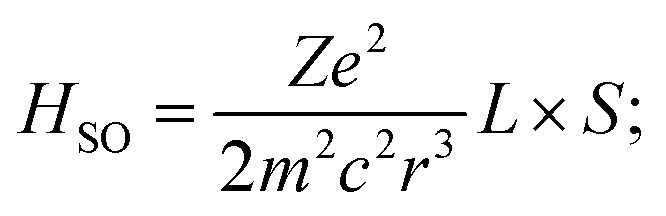
where *e*, *m*, and *c* are universal constants, *r* is the orbital radius, and *L* and *S* are orbital angular and spin angular momenta, respectively. Thus, the large SOC proposed for 2 is not merely a result of the heavy atom effect but is associated with advantageous spatial and orbital alignment of the iodide counterion, which plays a key role in enabling fast ISC within the ion pair.^48^

The computed rates of intersystem crossing (*k*_ISC_) for 1–3, 1^m^, 6 and 7 range from 10^8^ to 10^12^ s^−1^ (Table S9). While these values should be interpreted semi-quantitatively due to their high sensitivity to the selected method, they reflect a high probability of the S_1_ → T_1_/T_2_ processes, which are anticipated to be faster than relatively slow rates of symmetry-forbidden prompt fluorescence. Moreover, the ultrafast *k*_ISC_ (S_1_ → T_2_) rate predicted for 2 aligns with femtosecond emission up-conversion analysis, where the early relaxation is faster than 500 fs (Fig. S17).

Thus, short anion–cation contacts in 1^m^ and 2, *i.e.*, I⋯N/I⋯C (I⋯centroid) 3.62 (3.88) and 3.54 (3.88) Å, respectively, are considered to be the main reason for the realization of exceptionally rapid radiative rates of 1.32 × 10^5^ and 0.91 × 10^5^ s^−1^. These few to ten microseconds of intrinsic lifetimes *τ*_r_ (*τ*_r_ = 1/*k*_r_), *i.e.*, 7.6 μs for 1^m^ and 10.9 μs for 2, are rarely found for metal-free emitters. The elongated distance in 6 gives a high value but an order of magnitude lower value of 1.1 × 10^4^ s^−1^ (*τ*_r_ = 90.9 μs). The calculated radiative rates for 1° and 2 show a gradual decrease upon the increase of the anion–π distance (Table S10) that is consistent with the observed trend across the studied pyridinium ion pairs. Notably, the high radiative rates observed for phosphorescence can be rationalized by SOC-induced intensity borrowed from spin-allowed singlet–singlet transitions, resulting in a significant enhancement of the transition dipole moment associated with the spin-forbidden triplet–singlet transition.^48,49^ Qualitatively, this relationship can be expressed as
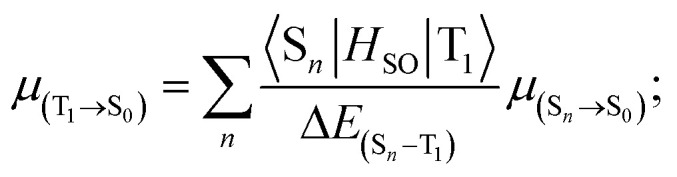
where *μ* is the transition dipole moment, <S_*n*_|*H*_SO_|T_1_> is the spin–orbit coupling matrix element, and Δ*E*(S_*n*_–T_1_) is the energy difference between the singlet excited state (S_*n*_) and the lowest lying triplet state (T_1_).

Tables S11 and S12 summarize the calculated SOCMEs, energy differences between T_1_ and S_2–10_ excited states, and oscillator strengths (*f*) for the spin-allowed S_*n*_ → S_0_ transitions. In all ion pairs studied, strong SOC is observed between T_1_ and S_2_/S_3_ with SOCMEs ranging from 748 cm^−1^ up to 2113 cm^−1^ (Tables S11 and S12). In turn, for the transitions between T_1_ and higher-lying singlet states (S_*m*, *m*>3_), the SOCMEs are predicted to be weaker, *i.e.*, between 2 cm^−1^ and 74 cm^−1^. The S_2_ and S_3_ states are also energetically reasonably close to the T_1_ state, with computed energy gaps between 0.10 eV and 0.72 eV. Thus, given their moderate oscillator strengths (*f* = 0.0005–0.01757, for S_2_/S_3_ → S_0_ transitions in 1°, 1^m^, 2, 3, 6, and 7), these states are likely the dominant contributors to efficient intensity borrowing for the T_1_ → S_0_ phosphorescence process.

The photophysical performance of salt 2 prompted its evaluation as a scintillation material. Indeed, the radioluminescence (RL) of ground 2 correlates with its photoluminescence behavior at both low and room temperatures (Fig. S18).

**Fig. 4:**
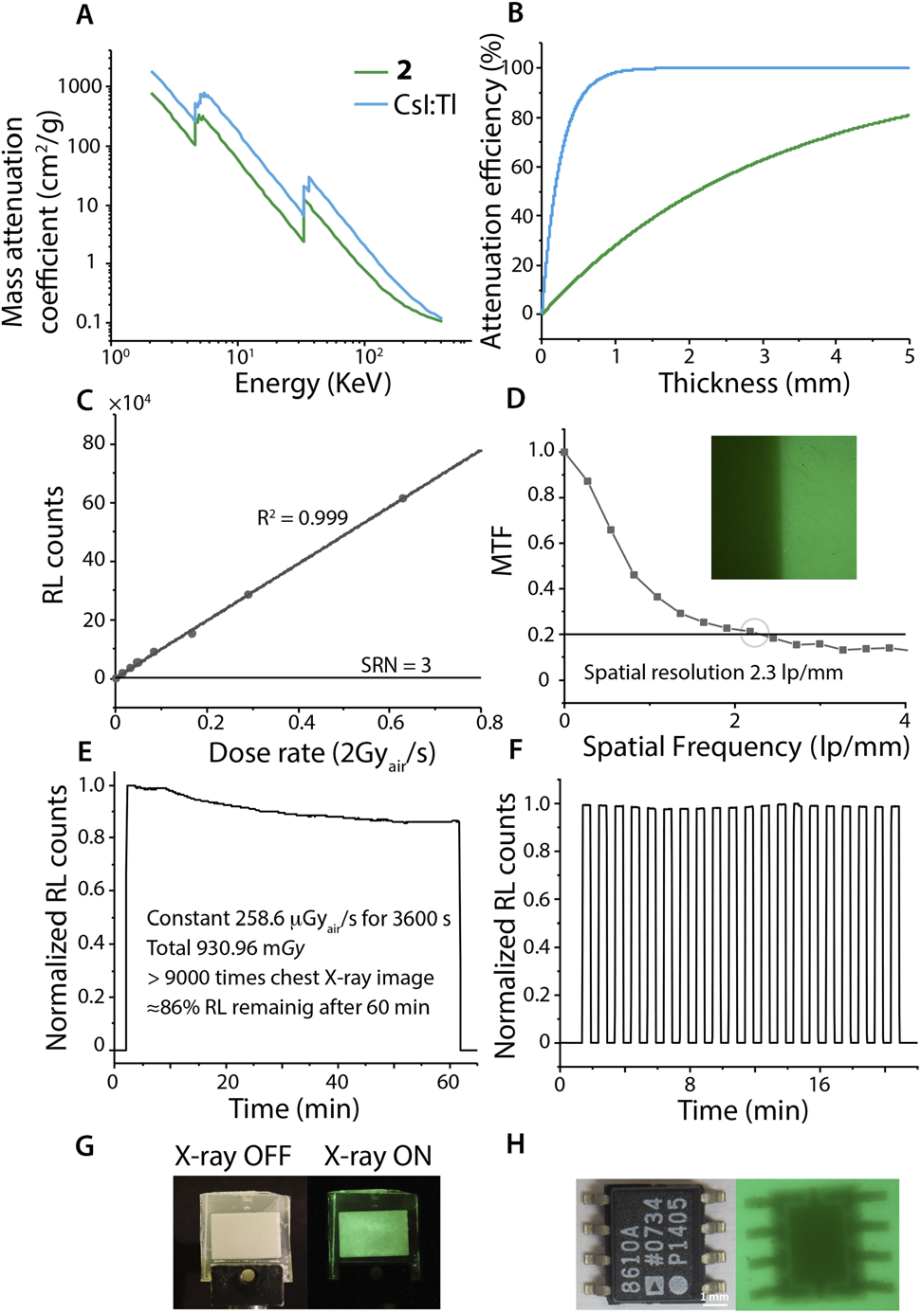
X-ray scintillation performance: (A) the plot of absorption coefficients as a function of photon energy for solid 2 and a CsI:Tl standard; (B) thickness-dependent attenuation efficiency of solid 2 and CsI:Tl at 30 KeV; (C) the linear relationship between RL intensity and the dose rate; (D) spatial resolution obtained by the slanted-edge method; (E) stability of the film upon continuous X-ray irradiation for 60 min; (F) pulse X-ray illumination stability test of the film (30 s ON; 30 s OFF). (G) The photograph of the film under ambient light and X-rays; (H) application of the film containing 2 in a safety check: an X-ray image of the chip.

The estimated light yield for 2 is 24 625 photons per MeV, compared to 54 000 photons per MeV for CsI:Tl. A linear correlation between RL intensity and the X-ray dose rate (Fig. 4A–C, *R*^2^ = 0.999) confirms the reliability of 2 for dose-rate-dependent detection, while the limit of detection (LoD) was determined to be 1 nGy s^−1^ (SNR = 3), following IUPAC guidelines.^50^ X-ray imaging was demonstrated using a scintillation screen composed of finely ground 2 and UV epoxy (1 mg: 2 μL), which retained the luminescent properties of the crystalline emitter (Fig. S19). Spatial resolution, assessed *via* the modulation transfer function (MTF) using the slanted-edge method (Fig. 4D and G), reached an appreciable 2.3 lp per mm at MTF = 0.2.

The stability of the scintillating screen was evaluated under continuous X-ray irradiation (approx. 258.6 μGy_air_ per s) over 60 minutes. The results show only a 14% decrease in radioluminescence intensity after a total dose of 930.96 mGy, which is equivalent to that of more than 9000 chest X-ray exposures (Fig. 4E). Under pulsed irradiation, the scintillating film exhibited remarkable stability, preserving 99% of its original RL intensity after a total dose of 155.16 mGy (Fig. 4F).

Finally, a practical X-ray image of an IC chip (Fig. 4H) revealed a clear contrast between metallic and plastic components, consistent with the strong, linear RL response of 2 across varying dose rates.

## Conclusions

In summary, we demonstrated a successful strategy for realizing strong RTP using a modular single pyridinium core. A tunable platform allows control of the degree of anion self-solvation, hence anion(I^−^)-π(pyridinium)^+^ distances, by introducing sterically enriched or hydroxyl groups. The rigid crystalline environment enforced by these interactions is decisive for suppressing nonradiative decay pathways and enabling efficient phosphorescence with radiative lifetimes in the microsecond range. Notably, the provitamer B6-based RTP materials exhibit high quantum yields up to 93% and rapid radiative rates of 0.9–1.3 × 10^5^ s^−1^, rivaling those of noble metal-based systems while being composed solely of earth-abundant, metal-free components.

The findings validate the utility of first generation pyridinium-based ionic charge-transfer emitters for scintillation applications and provide insights into molecular-level design principles for future high-performance organic photonic materials stemming from atom-photon principles, *i.e.*, maximizing performance with minimal atomic and synthetic complexity.

## Author contributions

Eetu Hakkarainen (data curation, formal analysis, and investigation), Hao-Cheng Lin (data curation and investigation), Anton A. Nechaev (conceptualization, investigation, methodology, and writing – review & editing), Vsevolod A. Peshkov (investigation and writing – review & editing), Toni Eskelinen (investigation, formal analysis, methodology, and writing – review & editing), Kai-Hsin Chang (investigation), Tzu-Hao Liao (formal analysis and investigation), Po-Yu Chen (investigation and resources), Igor O. Koshevoy (funding acquisition, project administration, writing – review & editing, and resources), Hao-Wu Lin (investigation, funding acquisition, project administration, and writing – review & editing), Pi-Tai Chou (conceptualization, supervision, funding acquisition, and writing – review & editing), and Andrey Belyaev, (conceptualization, validation, investigation, project administration, supervision, visualization, writing – original draft, and writing – review & editing).

## Conflicts of interest

There are no conflicts to declare.

## Supplementary Material

SC-OLF-D5SC03813H-s001

SC-OLF-D5SC03813H-s002

## Data Availability

The experimental details, including synthesis and characterization of all compounds, description of physical measurements, crystal data and structure refinement for 1°, 1^m^, 2–7, and *N*-EtpyI, selected structural parameters for these compounds, additional spectroscopic data, and figures, are given in the SI (PDF). Deposition numbers 2400506–2400512, 2418892, and 2420786 for 1°, 2–7, *N*–EtpyI, and 1^m^ contain the supplementary crystallographic data for this paper. These data are provided free of charge by the joint Cambridge Crystallographic Data Centre *via*www.ccdc.cam.ac.uk/structures. CCDC 2400506–2400512, 2418892, and 2420786 contain the supplementary crystallographic data for this paper.^51*a*–*i*^ The data supporting this article have been included as part of the SI. Synthetic experimental details, TGA, DSC and XRD data, NMR spectra, theoretical calculations and photophysical data, including steady state and time-resolved spectra. See DOI: https://doi.org/10.1039/d5sc03813h.
