# Supplementary material for: Metal-free pyridinium salts with strong room-temperature phosphorescence and microsecond radiative lifetime
Source: Chem Sci. 2025 Aug 15;16(37):17261–7. doi: 10.1039/d5sc03813h (PMC12394909; doi:10.1039/d5sc03813h)
Supplement: SC-016-D5SC03813H-s001 [file SC-016-D5SC03813H-s001.pdf]

Supplementary Information (SI) for Chemical Science.
This journal is © The Royal Society of Chemistry 2025

Supporting Information

Metal-free pyridinium salts with strong room-temperature phosphorescence and microsecond radiative lifetime

Eetu Hakkarainen,[a]‡ Hao-Cheng Lin,[b]‡ Anton A. Nechaev,*[c] Vsevolod A. Peshkov,[c] Toni Eskelinen,[d] Kai-Hsin Chang,[e] Tzu-Hao Liao,[b] Po-Yu Chen,[f] Igor O. Koshevoy,[a] Hao-Wu Lin,*[b] Pi-Tai Chou,*[e] Andrey Belyaev *[a]

[a] Department of Chemistry and Sustainable Technology, University of Eastern Finland, Yliopistokatu 7, 80100 Joensuu, Finland.

[b] Department of Materials Science and Engineering, National Tsing Hua University, 30013 Hsinchu, Taiwan, Republic of China

[c] Department of Chemistry/Nanoscience Center, University of Jyväskylä, Survontie 9B, 40500 Jyväskylä, Finland.

[d] Department of Chemistry and Materials Science, Aalto University, FI-00076 Aalto, Finland.

[e] Department of Chemistry, National Taiwan University, Taipei 10617, Taiwan, Republic of China

[f] Advanced Packaging Instrumentation and Metrology Laboratory, Industrial Technology Research Institute, Hsinchu 30013, Taiwan, Republic of China

‡ equal contribution

E-mail: anton.a.nechaev@jyu.fi, hwlin@mx.nthu.edu.tw, chop@ntu.edu.tw, andrei.beliaev@uef.fi

Contents

**General comments, instruments, and procedures**.	4
**Synthesis of pyridinium salts**	8
**Table S1.** Reported contact ion pairs (CIP) and their emissive properties.	13
**Table S2**. Selected structures of N-heterocyclic pyridinium and quinolinium alkyl salts and their shortest anion-π distances reported in the literature.	20
**Figure S1**. Crystal packing of the **N-MepyI** and **N-EtpyI** in the solid state, their photoluminescence properties at 77K.	22
**Figure S2**. TGA (left) and DSC (right) data for the studied salts **1**–**7**.	22
**Figure S3.** HPLC chromatograms of pyridinium salts **1**–**7**.	23
**Table S3.** Crystal data and structure refinement for **1–5**.	24
**Table S4.** Crystal data and structure refinement for **6**, **7**, $\mathbf{1^m}$, and **N–EtpyI**.	25
**Figure S4**. Fragments of the crystal packing with depicted stabilizing hydrogen bonding network H···**I** of $\mathbf{1^o}$ and $\mathbf{1^m}$.	26
**Table S5**. Crystal data and structure refinement for **6** at different temperatures (100–300 K).	27
**Table S6**. Crystal data and structure refinement for **2** at different temperatures (150–291 K).	28
**Figure S5**. Calculated ESP plots on a 0.02 a.u. isosurface for ionic pairs **1**–**7** and their cations.	29
**Figure S6**. Dimer formation and in plane H···**I** bonding in **6** and **7**.	29
**Figure S7**. VT scXRD data for the **6**.	30
**Figure S8**. VT scXRD data for the **2**.	30
**Figure S9**. Simulated and experimental PXRD patterns for $\mathbf{1^o}$, $\mathbf{1^m}$, **2**–**7**, **N-MepyI** and **N-EtpyI**.	31
**Figure S10**. Solid-state reflectance spectra of $\mathbf{1^o}$, **2**–**7** and $\mathbf{1^m}$.	32
**Figure S11**. HOMO/LUMO levels; excitation ($S_0 \rightarrow S_1$) and triplet emission (T_2, $T_1 \rightarrow S_0$) electron density difference plots for $\mathbf{1^o}$, **3** and **7**.	33
**Figure S12**. HOMO/LUMO levels; excitation ($S_0 \rightarrow S_1$) and triplet emission (T_2, $T_1 \rightarrow S_0$) electron density difference plots for $\mathbf{1^m}$ and **6**.	34
**Table S7**. Summary of the photophysical properties of pyridinium salts ($\mathbf{1^o}$, **3**, **4**, **7**, **N-MepyI** and **N-EtpyI**) in the solid state measured at 77 K.	35

Figure S13. Excitation, emission, and time-resolved spectra of the **1°**, **3**, **4** and **7** measured at 77 K. 36

Table S8. Summary of the photophysical properties of pyridinium salts (**1^{m}**, **2** and **6**). 37

Table S9. Calculated oscillator strengths, transition energies, radiative rate constants of phosphorescence, energy differences between the first excited states, spin-orbit coupling matrix elements between singlet and triplet states and intersystem crossing rates for **1°–3**, **1^{m}**, **6** and **7**. 38

Figure S14. Photoluminescence and lifetime of the excited state of **1^{m}**, **2,** and **6** measured under aerobic and anaerobic conditions. 39

Figure S15. Variable temperature excitation, emission profiles of solid samples **1^{m}** and **6**. 40

Figure S16. Excitation, emission, emission decay profile, and fit of solid sample **2** measured at 7 K. 40

Figure S17. Early relaxation dynamics of crystalline **2** (monitored at 450 nm) obtained by femtosecond emission up-conversion. 40

Table S10. Distance-dependent calculated radiative rates for **1°** and **2** using a Fermi Golden Rule approach. 41

Figure S18. Radioluminescence spectra of solid sample **2** upon X-Ray irradiation, and photoluminescence spectra measured upon excitation at 365 nm. 42

Figure S19. Emission spectrum of the composite film (1 mg of **2** mixed with 2 μl of epoxy resin). 42

Tables S11-12. Computed spin-orbit coupling matrix elements, energy differences between the lowest excited triplet state (T_1) and 10 lowest lying singlet excited states (S_n), and oscillator strengths (*f*) for the spin-allowed singlet-singlet transitions for **1°**, **1^{m}**, **2**, **3**, **6**, and **7** Calculated at the optimized T_1 geometries. 43

Figures S20–S33. ^{1}H and ^{13}C NMR data for salts **1–7**. 45

References 49

1. General comments, instruments, and procedures.

All reactions were performed under an inert atmosphere of argon (5.1 grade) or nitrogen (5.0 grade). Crystallization of the ionic pairs for spectroscopic measurements was performed under aerobic conditions. Solvents were used directly from the solvent purification system.

Melting points (mp.) were determined in open capillaries using the melting point apparatus Stuart Scientific SMP3. **IR** spectra were recorded on an FT-IR spectrometer Bruker Alpha. **HR-MS** spectra were obtained using an Agilent 6530C Q-TOF or Agilent 6560 ESI-IM-QTOF mass spectrometer equipped with an AJS ESI ion source. **TGA** measurements were carried out using a Mettler Toledo TGA 2 instrument in the temperature range of 30–600 °C with a heating rate of 10 °C min^{-1}, under a nitrogen atmosphere (flow rate 50 ml min^{-1}). 70 µl aluminum oxide pans were used for the experiments. Before the experiments, the pans were tempered at 1000 °C for 10 minutes and kept in an exicator to remove and protect from humidity. **DSC** measurements were carried out using a Mettler Toledo DSC 823^e instrument in the 25–400 °C temperature range with a heating rate of 10 °C min^{-1}, under a nitrogen atmosphere (flow rate 50 ml min^{-1}). 40 µl aluminum pans were used for the experiments, with an empty pan serving as a reference. **HPLC** chromatograms were obtained from an Agilent 1260 Infinity II liquid chromatograph equipped with a diode array detector (G71115A, monitored at 260 nm), binary pump, heated column compartment at 25 °C, and autosampler controller. Phenomenex Gemini 3μm C18 column (3.5-micrometer diameter particle size, 4.6×150 mm) was used for all analytical experiments. 2 μl aliquots (5–40 mM in H_2O, except for **3** and **4** in H_2O/10%-DMSO) were injected and eluted with H_2O/MeCN solution (TFA 0.1%) in a gradient mode (5-30% of MeCN) within 13 minutes. **Elemental analysis (EA)** was carried out in the analytical laboratory of the University of Eastern Finland and the University of Jyväskylä. **PXRD diffractograms** were collected from bulk recrystallized material (three averaged replicas from ground crystalline material) using a Rigaku XTALAB Synergy-S with Cu K_α radiation (λ = 1.54184 Å, Microfocus beam, Divergence 2.3, 600 sec exposure time) with Gandolfi strategy for data collection. Calculated data was generated using Mercury 2024.3.1 (Build 428092) software.

Photophysical measurements were performed under an inert atmosphere of argon. Prior to measurements, solid samples were dried under a vacuum (10^{-3} mbar) for at

least 30 minutes in order to avoid the influence of residual solvent and water. Both excitation and emission spectra of the samples were recorded on an Edinburgh Instrument FLS1000 spectrometer, equipped with a 450 W ozone-free Xenon arc lamp, double monochromators for the excitation and emission pathways, and a red-sensitive photomultiplier (PMT-980) as detector. The excitation and emission spectra were corrected using the standard corrections supplied by the manufacturer for the spectral power of the excitation source and the sensitivity of the detector. Variable temperature and low-temperature measurements were performed using Oxford Optistat DN 77K or Oxford 4K closed cycle cryostats (coupled with Edinburgh Instrument FLS980 spectrometer and PMT-920 detector). The solid samples' quantum yields were measured by using an integrating sphere in a direct mode (Edinburgh). The luminescence lifetimes were measured using an EP-LED pulsed laser diode (320, 365 nm), Xenon flashlamp, or VP-LED laser diode (375 nm) with multichannel-scaling (MCS) method. The emission was collected at right angles to the excitation source, with the emission wavelength selected using a double-grated monochromator and detected by the respective PMT. Solid-state reflectance spectra were measured using a PerkinElmer Lambda 900 UV/Vis/NIR spectrophotometer equipped with an integrating sphere. Fluoracle and FAST spectrometer operating software and Origin Pro 2019 9.6.0 were used for data analysis and processing.

Fluorescence up-conversion. Ultrafast fluorescence up-conversion measurements (FOG100-DX, CDP Corp.) were carried out using a stable 120-femtosecond laser oscillator (Tsunami, Spectra-Physics). The pump beam (368 nm) was generated from the fundamental output of the oscillator (736 nm) through second harmonic generation (SHG) using a β-barium borate (BBO) crystal. A neutral density (ND) filter and an iris diaphragm were employed to control the energy and beam diameter of the pump beam following SHG. A lens system was subsequently used to focus the pump beam onto the sample, collect the resulting fluorescence, and direct the fluorescence onto a BBO crystal (cut at 38°) for sum-frequency generation.

The crystalline samples were uniformly dispersed onto the sample cell, and measurements were performed in transmission mode using a rotating sample stage to ensure sample uniformity. The gate beam (736 nm) was routed through a mechanical delay line and intersected the fluorescence beam collinearly at the sum-frequency BBO crystal. The polarization angle between the pump and gate beams was set to the magic angle (54.7°) to suppress polarization-dependent artifacts arising

from molecular rotation. The generated sum-frequency signal was passed through a monochromator and detected by a photomultiplier tube (PMT). The full width at half maximum (FWHM) of the instrument response function (IRF) was approximately 220 femtoseconds.

Radioluminescence spectra were collected at room temperature and 150 K from single crystals using an Avantes AvaSpec-3648-spu2 (200–1100 nm) miniature spectrometer with optical fiber and collimating lens. Samples were placed under the cold nitrogen stream and irradiated continuously with CuKα (λ = 1.54184 Å) source (INCOATEC microfocus sealed tube, 50 kV, 1 mA). Spectra were obtained with an integration time of 20-50 seconds and further corrected for baseline dark counts.

Single crystal X-ray diffraction (scRXD): The single crystals of **1°**, **2–7, N–Etpyl,** and **1^m** were immersed in a film of NVH mounted on a polyimide microloop (MiTeGen), transferred to a stream of cold nitrogen (Cryostream 700 cooling system by Oxford Cryosystems), and measured at a temperature of 120–150 K. The X-ray diffraction data were collected on a Rigaku XtaLAB Synergy R or SuperNova Dualflex diffractometers with a HyPix-Arc 100 detector using mirror-monochromated Cu Kα (λ = 1.54184 Å) or Mo Kα (λ = 0.71073 Å) radiation (INCOATEC microfocus sealed tube). The frames were integrated with the CrysAlisPro software package using a narrow-frame algorithm. The CrysAlisPro program package was used for cell refinements and data reductions. The structure was solved using the intrinsic phasing method,[1,2] refined and visualized with the OLEX2-1.5 program.[3] A semiempirical absorption correction (SADABS) was applied to all data. All non-hydrogen atoms were refined anisotropically. Hydrogen atoms were included in structure factors calculations. All Hydrogen atoms were assigned to idealized geometric positions. The crystallographic details are summarized in Tables S3–6. CCDC **2400506–2400512, 2418892, and 2420786** contain supplementary crystallographic data for this paper.

Theoretical calculations were performed using the Orca (version 6.0.1) software package.[4] To model the solid-state materials, QM/MM (quantum mechanics/molecular mechanics) models were utilized. The experimentally obtained crystal structures were expanded in three dimensions to form a supercell. From the centre of the supercell, one cation-anion pair (with the shortest anion-π distance) was assigned as the QM layer and described at the density functional theory (DFT) level of theory. Surrounding ions in the supercell were assigned as the MM layer and their coordinates were kept fixed during optimization. The MM layer was described with a

simple forcefield generated with Orca's built-in makeff utility program. Electronic embedding was used to account for solid-state solvation effects. Ground state geometry optimizations were performed with DFT, while time-dependent DFT (TD-DFT) was utilized for excited state geometry optimizations using the PBE0 hybrid density functional[5] together with a def2-TZVP basis set and the corresponding effective core potential (ECP) for iodine atoms.[6] Frequency calculations were carried out for all optimized structures. Single-point TD-DFT calculations were performed for each optimized structure using the scalar-relativistic X2C Hamiltonian together with perturbative inclusion of spin-orbit coupling effects.[7,8] The relativistic calculations utilized the X2C-TZVPAll all-electron basis set for all atoms.[9] Radiative rates for phosphorescence and non-radiative rates for intersystem crossing were calculated using a Fermi Golden Rule approach implemented in Orca's excited state dynamics (ESD) module.[10] ESD calculations were performed in the Franck-Condon limit, neglecting contributions from Herzberg-Teller coupling. All calculations utilized the resolution-of-the-identity and chain-of-spheres for exchange (RIJCOSX) algorithms to reduce the computational burden.[11–14]

Scintillation film preparation: Crystalline **2** was finely ground and mixed with a UV Epoxy (ratio = 1 mg : 2 μl) using mortar and pestle. The mixture was poured into a glass cavity with a depth of 0.2 mm and cured under UV light for 5 minutes.

X-ray scintillator measurements: The X-ray attenuation efficiency was calculated from the following equation:

$$\text{X-ray attenuation efficiency (\%)} = (1-e^{-\mu t})\times 100\%$$

Where μ is the product of the attenuation coefficient and the density of the sample. The attenuation coefficient was obtained from the National Institute of Standards and Technology (NIST) database. The RL response and the limit of detection (LoD) were measured with a PMT (PMC-100-20, Becker & Hickl) combined with a single photon counter (SR400, Stanford Research Systems). The light yield was calculated considering the PMT responsivity across different wavelengths and the X-ray attenuation of the samples. All samples were measured under the same illuminated area and dose rate.

$$\frac{LY_{sample}}{LY_{CsI:Tl}} = \frac{R_{sample}/A_{sample}}{R_{CsI:Tl}/A_{CsI:Tl}} = \frac{\int I_{sample}(\lambda)S(\lambda)d\lambda / \int I_{sample}(\lambda)d\lambda}{\int I_{CsI:Tl}(\lambda)S(\lambda)d\lambda / \int I_{CsI:Tl}(\lambda)d\lambda} \div \frac{A_{sample}}{A_{CsI:Tl}}$$

Where R is the RL counts, I(λ) is the RL intensity, S(λ) is the responsivity of the PMT and the A is the equivalent X-ray attenuation efficiency.

The X-ray images were captured by a commercial camera (ZV-E10, Sony). The modulation transfer function (MTF) was calculated by the slanted-edge method from the following equation:

$$MTF\ (v) = F\ \big(LSF\ (x)\big) = F\ (\frac{dESF(x)}{dx})$$

The edge spread function (ESF) was obtained from the edge image generated using a 0.03 mm lead sheet. The line spread function (LSF) was derived by differentiating the ESF. The MTF was then obtained by performing a Fourier transform on the LSF.

2. Synthesis of pyridinium salts

N-MepyI. Pyridine (3 ml, 0.04 mol) was stirred in diethyl ether (20 ml) with methyl iodide (5 ml, 0.08 mol) for 12 h at room temperature in a sealed flask with no access to oxygen. Resulted white precipitate was filtered off, washed with diethyl ether (5×50 mL), and dried (8.4 g, yield 95%).

N-EtpyI. It was synthesized similarly to **N-MepyI** by stirring pyridine (3 ml, 0.04 mol) and iodoethane (6.4 ml, 0.08 mol) in diethyl ether (10 ml) for 72 h in a sealed flask with no access to oxygen. The obtained white precipitate was washed with diethyl ether (5×50 mL), dried, and used without any further purification (7.2 g, yield 77%). Single crystals suitable for scXRD measurements were obtained by evaporating a dichloromethane solution of **N-EtpyI** at room temperature.

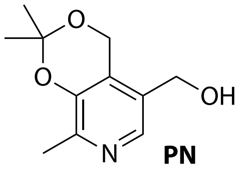

PN

(2,2,8-trimethyl-4H-[1,3]dioxino[4,5-c]pyridin-5-yl)methanol (PN). Synthesized according to a modified protocol.[15] To a solution of pyridoxine (3.4 g, 20.0 mmol) in acetone (57 mL) in a 250 mL round-bottom flask, 2,2-dimethoxypropane (39.2 mL, 320.0 mmol) and *p*-toluenesulfonic acid monohydrate (15.2 g, 80.0 mmol) were added subsequently. The reaction mixture was stirred at room temperature for 16 hours, then quenched with a saturated aqueous solution of $NaHCO_3$ to a neutral pH and extracted with DCM (3 × 100 mL). The combined organic layers were dried over Na_2SO_4, filtered, and concentrated under reduced pressure. Column chromatography on silica gel with hexanes/ethyl acetate (80/20 → 50/50 → 0/100) as eluent delivered the desired product as a pale powder (2.83 g, 67%). The recorded spectral data are found in agreement with previously reported values.

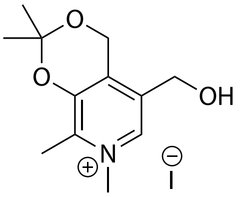

1

1. In a screw-cap vial under an argon atmosphere, pyridoxine **PN** (0.52 g, 2.5 mmol) was dissolved in dry dichloromethane (5 mL). After adding iodomethane (0.31 mL, 5 mmol), the reaction was stirred for 3 hours at 60°C until the starting material was consumed. The precipitate started to appear approximately after 1 hour. Upon the end of the reaction, the screw-cap vial was cooled down in an acetone bath to - 40 °C. The formed precipitate was separated and washed with diethyl ether (3×30 ml), and dried under vacuum to obtain **1** as white crystalline material (0.85 g, 96%). Single crystals were obtained by gas-phase diffusion of diethyl ether into an acetonitrile solution of **1**, whereas another non-solvated polymorph $\mathbf{1^m}$ was obtained from a hot saturated DCM solution of **1**.

MP 173 °C; **EA.** Calcd for $C_{12}H_{18}INO_3$: C 41.04%, H 5.17%, N 3.99%; Found: C 41.06%, H 5.77%, N 3.98%; **IR** (neat) ν_{max} 3349, 1332, 1202, 1072, 831 cm^{-1}; ^{1}**H NMR** (300 MHz, DMSO-*d*$_6$) δ 8.49 (s, 1H), 5.74 (bs, 1H), 5.10 (s, 2H), 4.61 (s, 2H), 4.24 (s, 3H), 2.56 (s, 3H), 1.57 (s, 6H); ^{13}**C NMR** (75 MHz, DMSO-*d*$_6$) δ 148.04, 144.43, 135.94, 134.46, 133.75, 102.52, 58.57, 57.50, 46.43, 24.84, 13.03; **HR-MS ESI$^+$: M$^+$** (m/z) found 224.1278 (calcd 224.1282).

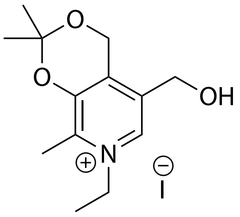

2

2. In a screw-cap vial under an argon atmosphere, pyridoxine **PN** (0.52 g, 2.5 mmol) was dissolved in dry dichloromethane (10 mL). After adding iodoethane (4.0 mL, 50 mmol), the reaction was stirred for 20 hours at 60°C until the starting material was consumed. After, the screw-cap vial was transferred to an ice-cold bath and diluted with diethyl ether. The precipitate was separated, washed with diethyl ether (3×30 ml) and dried. The crystalline material was further purified by subsequent crystallization from an acetonitrile-diethyl ether mixture, giving **2** as beige polycrystalline material (0.88 g, 96%). Single crystals were obtained by slow diffusion of diethyl ether into dichloromethane solution at room temperature.

MP 135 °C; **EA**. Calcd for $C_{13}H_{20}INO_3$: C 42.75%, H 5.52%, N 3.84%; Found: C 42.51%, H 5.78%, N 3.85%; **IR** (neat) ν_{max} 3370, 2981, 2858, 1306, 1074, 825 cm^{-1}; ^{1}**H NMR** (300 MHz, $CDCl_3$) δ 8.84 (s, 1H), 5.03 (s, 2H), 4.78 (s, 2H), 4.68 (q, *J* = 7.3 Hz, 2H), 2.66 (s, 3H), 1.68 (t, *J* = 7.3 Hz, 3H), 1.63 (m, 6H); ^{13}**C NMR** (75 MHz, $CDCl_3$) δ 149.18, 141.98, 136.42, 134.54, 134.32, 102.83, 59.01, 58.03, 54.84, 24.99, 15.77, 12.92. **HR-MS ESI$^+$: M$^+$** (m/z) 238.1440 (calcd 238.1438).

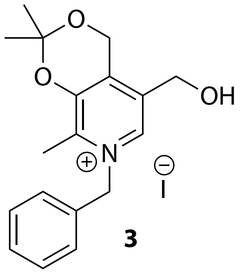

3. In a screw-cap vial under an argon atmosphere, pyridoxine **PN** (0.30 g, 1.4 mmol) and benzyl iodide (0.65 g, 3.0 mmol) were dissolved in dry acetonitrile (10 mL). The reaction was stirred for 12 hours at 60°C until the starting material was consumed. After, the reaction mixture was cooled in an ice-cold bath and diluted with diethyl ether (20 ml). The precipitate was filtered, washed with diethyl ether (3×30 ml) and dried. The crystalline material was further purified by subsequent crystallization from a dichloromethane-diethyl ether mixture, giving **3** as pale amorphous material (0.35 g, 57%). Single crystals were obtained by slow diffusion of diethyl ether into dichloromethane solution at room temperature, giving **3** as a DCM solvate.

MP 145 °C; **EA**. Calcd for $C_{18}H_{22}INO_3$: C 50.60%, H 5.19%, N 3.28%; Found: C 50.60%, H 5.09%, N 3.14%; **IR** (neat) ν_{max} 3294, 2996, 2930, 1630, 1605, 1579, 1497, 1309, 1075, 828 cm^{-1}; ^{1}**H NMR** (500 MHz, $CDCl_3$) δ 8.87 (s, 1H), 7.36–7.40 (s, 3H), 7.26–7.29 (s, 2H), 5.86 (s, 2H), 5.04 (s, 2H), 4.79 (s, 2H), 2.57 (s, 3H), 1.62 (m, 6H); ^{13}**C NMR** (125 MHz, $CDCl_3$) δ 149.57, 142.92, 136.55, 135.71, 134.99, 131.74, 129.64, 129,35, 127.59, 103.05, 62.78, 59.08, 58.07, 25.02, 13.33. **HR-MS ESI$^+$: M$^+$** (m/z) 300.1595 (calcd 300.1601).

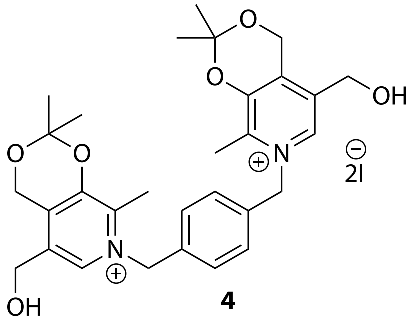

4. Was synthesized similarly to **3** from **PN** (0.42 g, 2.0 mmol) and diiodoxylene (0.32 g, 0.9 mmol) to give orange solid **4** (0.67 g, 96%). Single crystals were obtained by slow diffusion of diethyl ether into acetonitrile solution at room temperature giving **4** as pale-yellow needles.

MP 155 °C; **EA**. Calcd for $C_{30}H_{38}I_2N_2O_6$: C 46.41%, H 4.93%, N 3.61%; Found: C 46.56%, H 5.00%, N 3.50%; **IR** (neat) ν_{max} 3291, 3004, 2948, 1633, 1580, 1511, 1380, 1091, 828 cm^{-1}; ^{1}**H NMR** (500 MHz, DMSO-D_6) δ 8.59 (s, 2H), 7.30 (s, 4H), 5.93 (s, 4H), 5.11 (s, 4H), 4.64 (s, 4H), 2.48 (s, 6H), 1.57 (s, 12H); ^{13}**C NMR** (75 MHz, $CDCl_3$) δ 148.76, 143.98, 136.94, 134.71, 134.49, 134.28, 128.60, 102.87, 60.64, 58.67, 57.58, 55.50, 24.92, 13.19. **HR-MS ESI$^+$: M$^+$** (m/z) 261.1360 (calcd 261.1355).

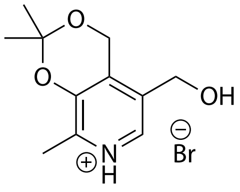

5

5. Pyridoxine **PN** (0.31 g, 1.5 mmol) was dissolved in methanol (10 ml), and HBr (aq., 20%, 2 ml) was added dropwise. The reaction mixture was stirred at room temperature for 5 minutes and evaporated to dryness. The obtained precipitate was dissolved in methanol (10 ml), and diethyl ether was added in portions (10×1 ml), causing the formation of needle-like transparent crystals. They were further separated, washed with diethyl ether (3×20 ml) and dried in vacuo to obtain **5** with a nearly quantitative yield (98%).

MP 210 °C; **EA**. Calcd for $C_{11}H_{16}BrNO_3$: C 45.53%, H 5.56%, N 4.83%; Found: C 45.34%, H 5.43%, N 4.99%; **IR** (neat) ν_{max} 3363, 2739, 2699, 1928, 1680, 1635, 1541, 1379, 1017, 841 cm^{-1}; **^{1}H NMR** (500 MHz, DMSO-d_4) δ 8.22 (s, 1H), 5.08 (s, 2H), 4.59 (s, 2H), 1.56 (s, *6*H); **^{13}C NMR** (125 MHz, DMSO-d_4) δ 147.75, 142.36, 136.07, 134.50, 129.84, 102.19, 58.64, 57.60, 24.87, 14.73. **HR-MS ESI$^+$: M$^+$** (m/z) 210.1127 (calcd 210.1125).

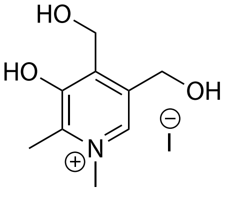

6

6. Pyridinium salt **1** (0.42 g, 1.2 mmol) was dissolved in a mixture of tetrahydrofuran (10 mL) and aqueous HCl (aq., 10%, 2 mL). The reaction was refluxed for 4 hours under an inert atmosphere, and then the resulting mixture was evaporated to dryness. Orange viscous oil was dissolved in an ethanol solution of *n*-Bu_4NI (1 g in 5 ml) and precipitated with diethyl ether (20 ml). The obtained material was washed with dichloromethane (3×15 ml) and recrystallized three times from hot ethanol (heating to reflux with subsequent cooling to -21 °C), washed additionally with dichloromethane, and dried, yielding white crystalline material **6** (0.32 g, 85%).

MP 190 °C; **EA.** Calcd for $C_9H_{14}INO_3$: C 34.75%, H 4.54%, N 4.50%; Found: C 34.80%, H 4.33%, N 4.67%; **IR** (neat) ν_{max} 3426, 3242, 3073, 1452, 1247, 1012, 763, 536 cm^{-1}; **^{1}H NMR** (300 MHz, DMSO-d_6) δ 8.44 (s, 1H), 4.81 (s, 2H), 4.69 (s, 2H), 4.23 (s, 3H), 2.60 (s, 3H); **^{13}C NMR** (75 MHz, DMSO-d_6) δ 152.69, 144.57, 140.00, 138.35, 135.06, 58.10, 56.51, 46.57, 13.83. **HR-MS ESI$^+$: M$^+$** (m/z) 180.0950 (calcd 180.0969).

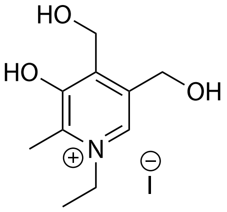

7

7. Pyridinium salt **2** (0.44 g, 1.2 mmol) was dissolved in a mixture of tetrahydrofuran (10 mL) and aqueous HCl (aq., 10%, 2 mL). The reaction was refluxed for 4 hours under an inert atmosphere, and then the resulting mixture was evaporated to dryness. The obtained oil was dissolved in ethanol solution of *n*-Bu_4NI (1g in 5 ml), and further precipitated with diethyl ether (20 ml). The obtained material was washed with

dichloromethane (3×15 ml) and recrystallized three times from an acetonitrile-diethyl ether mixture, yielding white crystalline material **7** (0.28 g, 71%).

MP 115 °C; **EA.** Calcd for $C_{10}H_{16}INO_3$: C 36.94%, H 4.96%, N 4.31%; Found: C 37.01%, H 4.87%, N 4.30%; **IR** (neat) ν_{max} 3390, 3223, 3072, 1448, 1286, 1017, 762, 549 cm^{-1}; **^{1}H NMR** (300 MHz, Methanol–d_4) δ 8.39 (s, 1H), 5.10 (s, 2H), 4.71 (s, 2H), 4.63 (q, *J* = 7.3 Hz, 2H), 2.72 (s, 3H), 1.58 (t, *J* = 7.3 Hz, 3H); **^{13}C NMR** (75 MHz, Methanol–d_4) δ 154.45, 143.21, 138.38, 137.28, 133.03, 59.02, 58.08, 53.77, 14.38, 11.40. **HR-MS ESI$^+$: M$^+$** (m/z) 198.1117 (calcd 198.1125).

Table S1. Reported contact ion pairs (CIP) and their emissive properties.

Structure	Counter anions / R-groups	Anion-π interaction (Å)	Emission maxima, λ_{em} (nm)	Quantum yield, Φ_{em}	Lifetime, t_{av}[a]	Radiative rate, k_r (s^{-1})[b]	Reference, additional notes
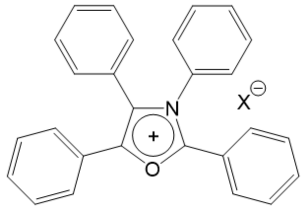	X = I	3.98	559	0.16 (P, powder)<br>0.35 (P, SC)	48.74 μs	3.28×10^3 (P, powder)<br>7.18×10^3 (P, SC)	[16,17]<br><br>Average fluorescence or phosphorescence lifetime calculated by $\tau = \Sigma A_i \tau_i^2 / \Sigma A_i \tau_i$, where A_i is the pre-exponential factor for lifetime τ_i
	X = Br	3.49	434 (F) 549 (P)	0.18 (F)<br>0.37 (P, SC)	2.52 ns (F)<br>706.42 μs (P, SC)	7.14×10^7 (F)<br>5.24×10^2 (P, SC)	
	X = Cl	-	435	0.20	1.60 ns	1.25×10^8	
	X = F	-	420	0.11	0.80 ns	1.38×10^8	
	X = PF_6	2.88*	422	0.19	1.02 ns	1.86×10^8	
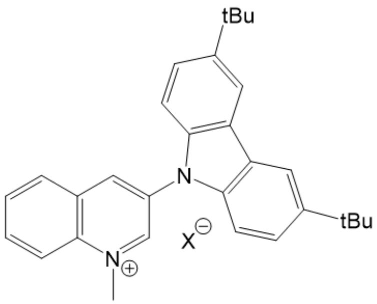	X = I	-	586	-	-	-	[18]<br><br>Measured in 1wt% PMMA films
	X = OTf	3.69	578 (PMMA matrix)<br>574 (neat film)	0.04 (PMMA matrix)<br>0.13 (neat film)	28 ns (PF, neat film)<br>9 μs (TADF, neat film)	1.44×10^4 (TADF, neat film)	
	X = BF_4	4.84	581	-	-	-	
	X = I	-	590	-	-	-	[18]<br><br>Measured in 1wt% PMMA films
	X = OTf	4.01	581 (PMMA matrix)<br>582 (neat film)	0.13 (PMMA matrix)<br>0.34 (neat film)	15 ns (PF, neat film)	4.86×10^4 (TADF, neat film)	

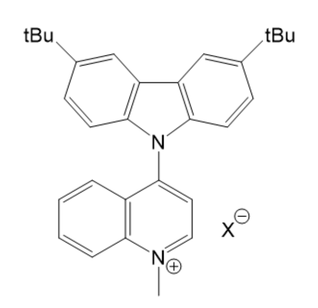					7 µs (TADF, neat film)		
	X = BF_4	3.70	585	-	-	-	
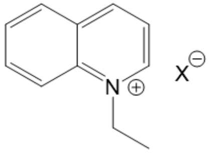	X = I	4.27	-	-	-	-	
	X = Br	3.84	551	-	286 µs	-	
	X = PF_6	4.96	412	-	11.20 ns	-	
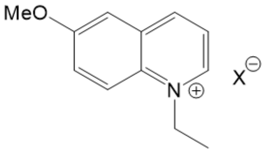	X = I	4.00	-	-	-	-	
	X = Br	3.99	547	-	490 µs	-	[19]
	X = PF_6	4.28	411	-	5.96 ns	-	
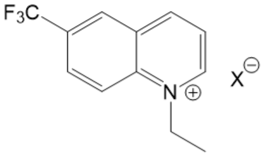	X = I	3.78	615	-	1.44 µs	-	
	X = Br	3.83	533	-	80.40 µs	-	
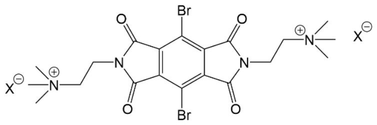	X = I	3.69	470 (LE) 560 (CT)	0.11* (in air) 0.40* (vacuum)	0.62 ms (LE, in air) 0.89 (LE, vacuum)	1.77 $\times10^2$ (LE, in air) 4.49 $\times10^2$ (LE, vacuum) 1.69 $\times10^2$ (CT, in air)	[20] Drop-casted films, concentration of the hybrid solution used for drop-casting is 1

					0.65 ms (CT, in air)<br>0.99 ms (CT, vacuum)	4.04×10^{2} (CT, vacuum)	mM of **BrPmDI-LP** and 5 wt % **LP** (laponite) in water<br><br>*Absolute phosphorescence quantum yield
	X = Br	-	-	-	-	-	
	X = BF_4	-	-	-	-	-	
	X = BPh_4	-	-	-	-	-	
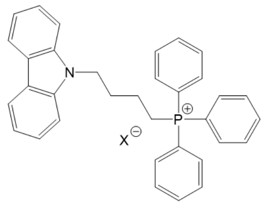	X = I	3.93	375, 606	0.0771	17 ms (606)	4.54 ($\times 10^{0}$)	[21]
	X = Br	3.78	375, 411, 555, 606, 664	0.0397	81 ms (555)<br>84 ms (606)<br>80 ms (664)	4.90×10^{-1} (555)<br>4.73×10^{-1} (606)<br>4.96×10^{-1} (664)	
	X = Cl	3.74	375, 606	0.0745	112 ms (606)	6.65×10^{-1}	
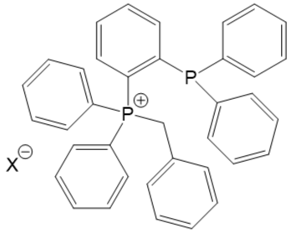	X = I	-	529	0.10	44.22 µs	2.26×10^{3}	[22]<br><br>T = 300 K<br><br>Deposition number for X = Br was given (2225928), but the structure was not found.
	X = Br	-	522	0.0137 (F)<br>0.27 (TADF)	2.37 ns (F)<br>55.63 µs (TADF)	5.78×10^{6} (F)<br>4.85×10^{3} (TADF)	
	X = Cl	-	522	0.07	2.98 ns	2.35×10^{7}	

	X = Ac	-	581	-	-	-	
	X = ClO_4	-	540	-	-	-	
	X = BF_4	-	534	-	-	-	
	X = NO_3	-	538	0.065	19.85 μs	3.27×10^3	
	X = CF_3SO_3	-	541	0.051	20.75 μs	2.46×10^3	
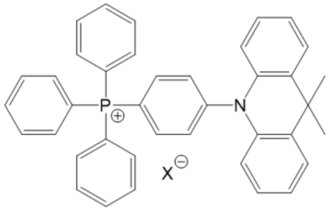	X = Br	4.80*	518	0.22 (PF)<br>0.41 (TADF)	14.7 ns (PF)<br>1.6 μs (TADF)	1.50×10^7 (PF)<br>2.56×10^5 (TADF)	[23]<br><br>Measured using 30 wt% doped emitters in PMMA films<br><br>Measured at room temperature<br><br>*Cation-anion distance (crystal data is reported, deposition numbers were not given)
	X = BF_4	6.50*	512	0.23 (PF)<br>0.50 (TADF)	16.7 ns (PF)<br>1.8 μs (TADF)	1.38×10^7 (PF)<br>2.78×10^5 (TADF)	
	X = $BArF_{24}$	9.00*	507	0.23 (PF)<br>0.45 (TADF)	15.8 ns (PF)<br>2.2 μs (TADF)	1.46×10^7 (PF)<br>2.05×10^5 (TADF)	
	R = CH_3	-	487	0.428<br>(overall Φ_{em})	-	-	[24]<br><br>T = 300 K

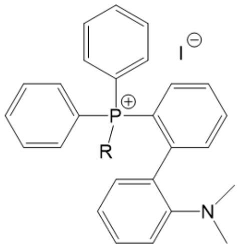	R = C_4H_9	-	473	0.0086 (PF) 0.9914 (TADF)	18.55 ns (PF) 469.01 μs (TADF)	4.64×10^5 (PF) 2.11×10^3 (TADF)	Deposition number for R = C_4H_9 was given (2356422), but the structure was not found.
	R = C_6H_{13}	-	492	0.639 (overall Φ_{em})	-	-	
	R = C_8H_{17}	-	-	-	-	-	
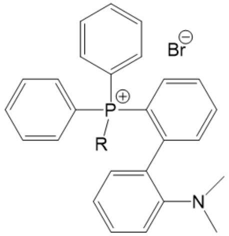	R = C_4H_9	-	478	0.4333 (PF) 0.2307 (TADF)	16.22 ns (PF) 155.29 μs (TADF)	2.46×10^7 (PF) 1.49×10^3 (TADF)	
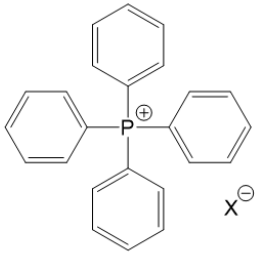	X = I	6.24	430	0.42	9.90 μs	4.24×10^4	[25] T = 298 K
	X = Br	5.55	486	0.04	6.04 ms	6.62 ($\times 10^0$)	
	X = Cl	4.80	400 (F) 500 (P, AG)	<0.01 (F) 0.02 (P, AG)	0.90 ns (F) 108.12 ms (P, AG)	1.11×10^7 (F) 1.85×10^{-1} (P, AG)	
	X = F	-	400 (F) 480 (P, AG)	<0.01 (F) 0.02 (P, AG)	1.60 ns (F) 25.70 ms (P, AG)	6.25×10^6 (F) 7.78×10^{-1} (P, AG)	
	X = BF_4	6.28	430 (P) 490 (P, AG)	0.48 (P) <0.01 (P, AG)	4.5 μs (P) 43.41 ms (P, AG)	1.07×10^5 (P) 2.30×10^{-1} (P, AG)	[26] T = 298 K

	X = ClO_4	6.34	420 (P)<br>475 (P, AG)	0.56 (total Φ_{em})	2.8 μs (P)<br>399.71 ms<br>(P, AG)	2.00×10^5 (P)<br>1.40 ($\times 10^0$)<br>(P, AG)	
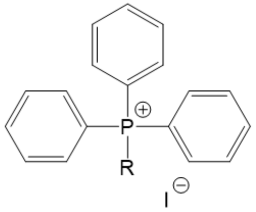	R = CH_3	3.94	487	0.26 / 0.17	3.4 μs / 2.1 μs	0.76×10^5 / 0.81×10^5	[25]<br>/<br>[27] (CCDC 1977060)
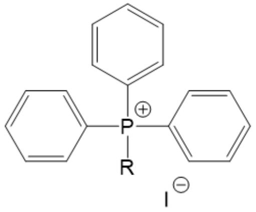	R = CH_3	3.94	490	0.039	1.05 ms	228.57 ($\times 10^0$)	[28]<br><br>Data for PPh_3MeI does not match previous reports.<br><br>R = alkyl chain<br><br>T = 298 K
	R = C_2H_5	-	482	0.0666	2.911 ms	22.67 ($\times 10^0$)	
	R = C_3H_7	3.89	475	0.097	4.28 ms	22.66 ($\times 10^0$)	
	R = C_4H_9	3.87	483	0.1664	8.31 ms	19.74 ($\times 10^0$)	
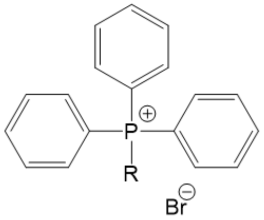	R = CH_3	3.83	486	0.0465	43 ms	1.08 ($\times 10^0$)	
	R = C_2H_5	3.77	465, 494	0.0260	25 ms	1.04 ($\times 10^0$)	
	R = C_3H_7	3.75	455, 481	0.0621	146 ms	0.425 ($\times 10^0$)	
	R = C_4H_9	3.72	455, 476	0.0846	229 ms	0.368 ($\times 10^0$)	
	R = C_5H_{11}	-	462, 490	0.0096	9.32 ms (462),<br>10.46 ms (490)	1.03 ($\times 10^0$, 462)<br>0.92 ($\times 10^0$, 490)	
	R = C_6H_{13}	-	452, 482	0.0147	1.89 ms (452), 4.86 ms (482)	7.78 ($\times 10^0$, 452)<br>3.02 ($\times 10^0$, 482)	

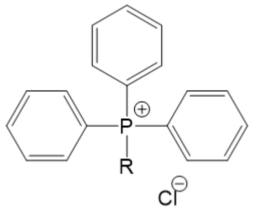	R = CH_3	-	486	0.045	284.6 ms	0.158 (×10^0)	[28] R = alkyl chain T = 298 K
	R = C_2H_5	-	495	0.2447	279.26 ms	0.513 (×10^0)	
	R = C_3H_7	-	481	0.1432	508.22 ms	0.28 (×10^0)	
	R = C_4H_9	3.64	479	0.4919	739.18 ms	0.62 (×10^0)	
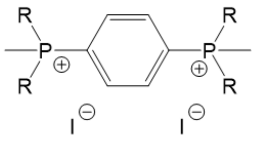	R = Ph	4.17	560	0.54	2.13 μs	2.54×10^5	[27] T = 297 K
	R = iPr	4.27	520	0.75	2.71 μs	2.77×10^5	
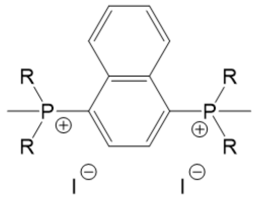	R = Ph	3.98	645	0.07	0.46 μs	1.64×10^5	

[a] Amplitude-weighted average emission lifetimes for the biexponential decays determined by the equation $\tau_{av}=\Sigma A_i\tau_i$, A_i=weight of the i-th exponent, unless otherwise stated.
[b] k_r were estimated by Φ_{em}/τ_{av}
Abbreviations: F = fluorescence, P = phosphorescence, PF = prompt fluorescence, TADF = thermally activated delayed fluorescence, AG = afterglow, SC = single crystal, LE = locally excited, CT = charge transfer

Table S2. Selected structures of N-heterocyclic pyridinium and quinolinium alkyl salts and their shortest anion-π distances reported in the literature.

	CCDC code	Shortest anion – π-cation interaction (Å)
1-ethyl-3,5-bis{[(3,3,4,4,5,5,6,6,7,7,8,8,8-tridecafluorooctyl)oxy]carbonyl}pyridin-1-ium iodide	1550606 / LEDSEV	3.489
3,5-bis(methoxycarbonyl)-1-methylpyridinium iodide	983186 / CONQIH	3.500/3.512
1-[2-oxo-2-(quinolin-8-yl)ethyl]pyridin-1-ium iodide	1581997 / PEHGUH	3.515
3-iodo-1-(3-iodopropyl)pyridin-1-ium iodide	2109495 / CAGPEJ	3.571
3-bromo-1-ethylpyridin-1-ium iodide	2109491 / CAGNIL	3.575
3-(4-Ethylpyridinium)-1,5-diphenylverdazyl radical iodide	249720 / LEBGUV	3.610
Dipyridinio-methane di-iodide	1144780 / DOWVAM	3.620, 3.703
1,1'-methylenebis(pyridin-1-ium) diiodide monohydrate	2174457 / LIBLUH	3.664
bis(4-Methylpyridin-1-io)methane bis(iodide) monohydrate	180871 / TAFMIX	3.667, 3.703
1-Ethyl-2-methyl-3-nitro-pyridinium iodide	1211146 / MEPRIC	3.714
1-((benzyloxy)carbonyl)-3-(iodomethyl)-2,3-dihydro-1H-imidazo[1,2-a]pyridin-4-ium iodide toluene solvate	1563634 / DURTUI	3.720
3-chloro-1-ethylpyridin-1-ium iodide	2109490 / CAGNEH	3.729
1-(2-hydroxyethyl)pyridinium iodide	2093335 / HEHQEU	3.733
1-(propan-2-yl)pyridin-1-ium iodide	1970366 / DOVQEN	3.734
2-(iodomethyl)-5,7-dimethyl-2,3-dihydro[1,3]oxazolo[3,2-a]pyridin-4-ium iodide	1984103 / GUHSUA	3.734
1-ethylquinolinium iodide	1507135 / AXAHEO	3.756
N-Methylpyridinium iodide	1214289 / MPYRMI	3.761
1-(2-Oxo-2-phenylethyl)pyridinium iodide	814012 / UTEZEZ	3.761
1-butylpyridin-1-ium iodide	2375766 / LOXLIX	3.767
1-(Pentafluorobenzyl)pyridinium iodide	936266 / XIVJAP	3.772
1,1'-[1,4-phenylenebis(methylene)]bis(pyridin-1-ium) bis(iodide)	2341090 / XOTKAW	3.773
4-Amino-(1-ethoxycarbonylmethyl)pyridinium iodide	610850 / ADAYEK	3.773
1-ethyl-6-(trifluoromethyl)quinolinium iodide	1507145 / AXAKER	3.775
1-ethyl-3-iodopyridin-1-ium iodide	2109492 / CAGNOR	3.778
1-ethyl-2-methylpyridin-1-ium iodide	1969586 / COYHAC	3.824
1-((1-Methyl-5-nitro-1H-imidazol-2-yl)methyl)pyridinium iodide	1045139 / MOYHOZ	3.847

3-Anilino-1-isopropylpyridinium iodide	838685 / SATMUX	3.876
1-(2-oxopropyl)pyridin-1-ium iodide	1983259 / KUMFUW	3.887
4-(Dimethylamino)-1-ethylpyridinium iodide	1208687 / LUJVUH	3.933
N(1)-(2,6-Dichlorobenzyl)-3-carbamido-pyridinium iodide monohydrate	1137378 / DCAPYR	3.954
1-[2-oxo-2-(pyrazin-2-yl)ethyl]pyridin-1-ium iodide	1872474 / RIMNOT	3.966
1-butyl-4-(pyrrolidin-1-yl)pyridin-1-ium iodide	1992665 / ZUPBOE	3.984
trans-4-((N-Methylpyrrol-2-yl)styryl)-N-ethylpyridinium iodide	720298 / XUVVEQ	4.080
1-heptyl-4-[2-(2-hydroxynaphthalen-1-yl)ethenyl]quinolin-1-ium iodide	1584172 / ZEMKEK	4.115

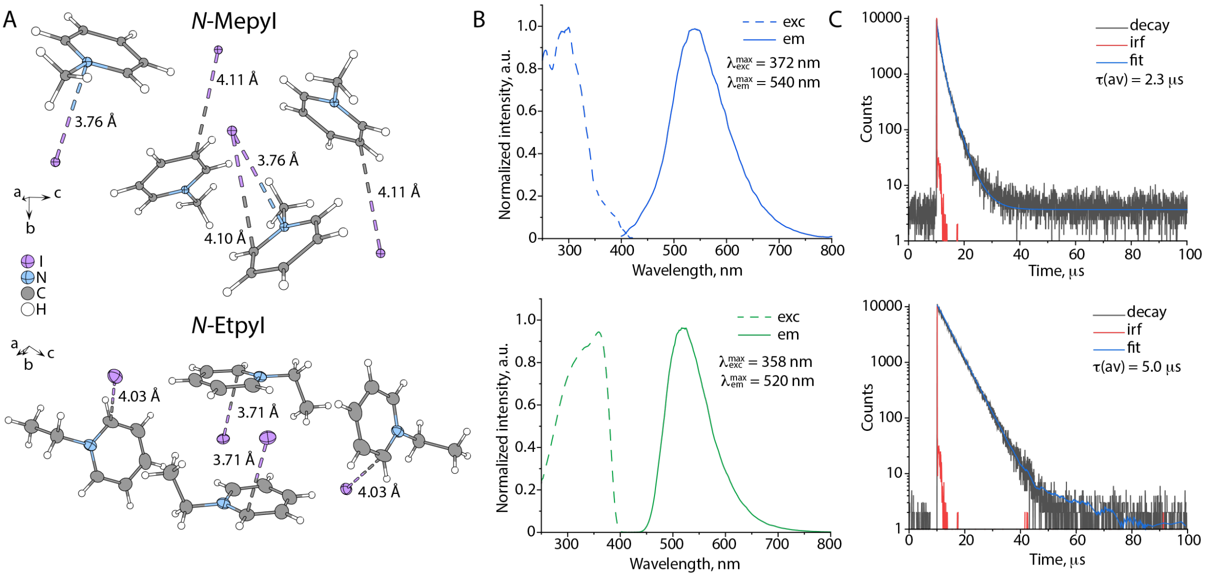

Figure S1. (A) Fragment of crystal packing of the **N-MepyI** (upper, ball and stick representation, CCDC database – MPYRMI) and **N-EtpyI** (bottom, displacement ellipsoids are shown at the 50% probability level) in the solid state obtained by scXRD with depicted shortest anion-cation distances; (B) Emission and excitation profiles of the **N-MepyI** and **N-EtpyI** measured at 77 K; (C) Time-resolved emission decay curves detected at 77 K for **N-MepyI** and **N-EtpyI**. See Table S7 for more details.

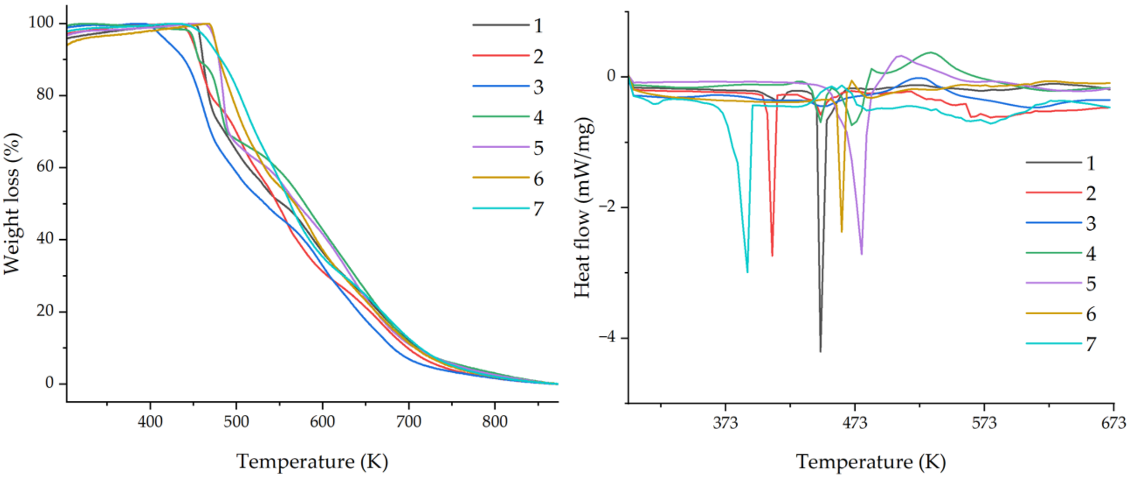

Figure S2. TGA (left) and DSC (right) data for the studied salts **1–7**.

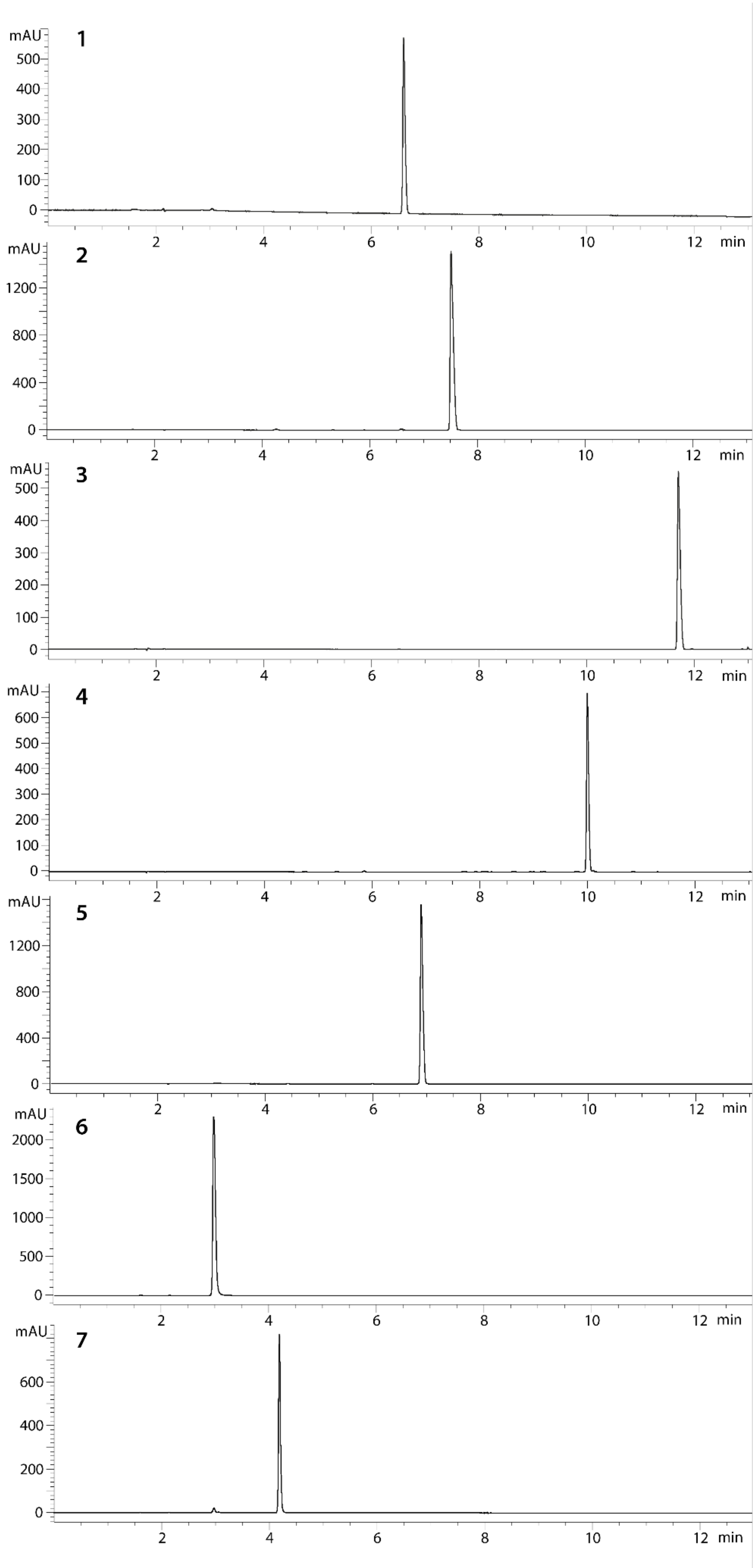

Figure S3. HPLC chromatograms of pyridinium salts **1–7**.

Table S3. Crystal data and structure refinement for **1–5**.

Identification code	**1°**	**2**	**3**	**4**	**5**
CCDC number	2400506	2400507	2400508	2400509	2400510
Empirical formula	$C_{12}H_{18}INO_3$	$C_{13}H_{20}INO_3$	$C_{74}H_{88}Cl_4I_4N_4O_{12}$	$C_{30}H_{38}I_2N_2O_6$	$C_{11}H_{16}BrNO_3$
Formula weight	351.17	365.20	1874.88	776.42	290.16
Temperature [K]	120.00(10)				
Crystal system	orthorhombic	monoclinic	monoclinic	triclinic	monoclinic
Space group	Pbca	Cc	$P2_1/n$	P-1	$P2_1/c$
a [Å]	9.8944(4)	8.5894(2)	15.38142(13)	8.94630(10)	9.27720(10)
b [Å]	10.0780(4)	16.0241(4)	9.39589(8)	12.7534(3)	16.3448(2)
c [Å]	27.6271(11)	10.5135(3)	27.9600(2)	17.3129(3)	7.94350(10)
α [°]	90	90	90	105.939(2)	90
β [°]	90	92.256(2)	104.7932(9)	93.744(2)	99.3750(10)
γ [°]	90	90	90	94.0170(10)	90
Volume [$Å^3$]	2754.86(19)	1445.93(6)	3906.90(6)	1887.35(6)	1188.42(2)
Z	8	4	2	2	4
ρ_{calc} [g/cm^3]	1.693	1.678	1.594	1.366	1.622
μ [mm^{-1}]	2.322	2.216	14.273	13.383	4.673
F(000)	1392.0	728.0	1872.0	772.0	592.0
Crystal size [mm^3]	0.092 × 0.091 × 0.077	0.101 × 0.099 × 0.076	0.076 × 0.034 × 0.021	0.301 × 0.031 × 0.028	0.18 × 0.14 × 0.03
Radiation type	Mo Kα (λ = 0.71073)	Mo Kα (λ = 0.71073)	Cu Kα (λ = 1.54184)	Cu Kα (λ = 1.54184)	Cu Kα (λ = 1.54184)
2Θ range for data collection [°]	5.064 to 67.934	5.384 to 62.844	6.008 to 158.352	7.694 to 158.476	9.662 to 153.04
Index ranges	-13 ≤ h ≤ 11, -14 ≤ k ≤ 9, -34 ≤ l ≤ 42	-12 ≤ h ≤ 11, -22 ≤ k ≤ 21, -14 ≤ l ≤ 14	-19 ≤ h ≤ 19, -10 ≤ k ≤ 11, -35 ≤ l ≤ 35	-9 ≤ h ≤ 11, -16 ≤ k ≤ 16, -21 ≤ l ≤ 21	-11 ≤ h ≤ 11, -7 ≤ k ≤ 20, -9 ≤ l ≤ 9
Reflections collected	17541	6163	55764	42103	10040
Independent reflections	4466 [R_{int} = 0.0304, R_{sigma} = 0.0287]	2913 [R_{int} = 0.0238, R_{sigma} = 0.0306]	8382 [R_{int} = 0.0434, R_{sigma} = 0.0253]	8064 [R_{int} = 0.0946, R_{sigma} = 0.0606]	2456 [R_{int} = 0.0293, R_{sigma} = 0.0259]
Data/restraints/parameters	4466/0/159	2913/2/168	8382/0/453	8064/7/374	2456/0/149
Goodness-of-fit on F^2 (a)	1.063	1.043	1.026	1.094	1.063
Final R indexes [I>=2σ (I)] (b)	R_1 = 0.0250, wR_2 = 0.0631	R_1 = 0.0181, wR_2 = 0.0414	R_1 = 0.0375, wR_2 = 0.0995	R_1 = 0.0697, wR_2 = 0.1966	R_1 = 0.0248, wR_2 = 0.0642
Final R indexes [all data] (b)	R_1 = 0.0347, wR_2 = 0.0671	R_1 = 0.0188, wR_2 = 0.0419	R_1 = 0.0419, wR_2 = 0.1033	R_1 = 0.0804, wR_2 = 0.2054	R_1 = 0.0278, wR_2 = 0.0657
Largest diff. peak/hole [$e/Å^{-3}$]	1.02/-0.63	0.36/-0.33	1.70/-1.15	3.02/-1.10	0.64/-0.34
Flack parameter	–	-0.027(18)	–	–	–

(a) GooF = S = $[[\sum w(F_o^2-F_c^2)^2]/(m-n)]^{1/2}$, where m = number of reflexes and n = number of parameters; (b) $R_1 = \sum||F_o|-|F_c||/\sum|F_o|$; $wR_2 = [\sum[w(F_o^2-F_c^2)^2/\sum[(wF_o^2)^2]]^{1/2}$; $w = 1/[\sigma^2(F_o^2)+(aP)^2+bP]$, where $P = (F_o^2+2F_c^2)/3$

Table S4. Crystal data and structure refinement for **6**, **7**, **1^{m}**, and **N–EtpyI**.

Identification code	**6**	**7**	**1^{m}**	**N–EtpyI**
CCDC number	2400511	2400512	2418892	2420786
Empirical formula	$C_9H_{14}INO_3$	$C_{40}H_{64}I_4N_4O_{12}$	$C_{12}H_{18}INO_3$	$C_7H_{10}IN$
Formula weight	311.11	1300.55	351.17	235.06
Temperature [K]	100.00(10)	120(11)	150.01(10)	150.01(10)
Crystal system	monoclinic	monoclinic	monoclinic	monoclinic
Space group	$P2_1/n$	$P2_1/n$	$P2_1/c$	$P2_1/c$
a [Å]<br>b [Å]<br>c [Å]<br>α [°]<br>β [°]<br>γ [°]	6.62750(10)<br>11.30050(10)<br>14.8296(2)<br>90<br>98.5070(10)<br>90	6.3438(2)<br>12.3508(4)<br>15.0717(4)<br>90<br>98.931(3)<br>90	14.3171(4)<br>10.7495(4)<br>8.9701(2)<br>90<br>90.739(2)<br>90	9.09315(15)<br>8.55139(12)<br>11.5954(2)<br>90<br>106.7085(19)<br>90
Volume [Å^3]	1098.43(2)	1166.57(6)	1380.40(7)	863.58(3)
Z	4	1	4	4
ρ_{calc} [g/cm^3]	1.881	1.851	1.690	1.808
μ [mm^{-1}]	22.797	2.734	18.220	28.490
F(000)	608.0	640.0	696.0	448.0
Crystal size [mm^3]	0.03 × 0.015 × 0.015	0.171 × 0.101 × 0.023	0.129 × 0.102 × 0.098	0.078 × 0.055 × 0.03
Radiation type	Cu Kα (λ = 1.54184)	Mo Kα (λ = 0.71073)	Cu Kα (λ = 1.54184)	Cu Kα (λ = 1.54184)
2Θ range for data collection [°]	9.882 to 159.232	4.284 to 67.804	6.174 to 129.958	10.156 to 158.876
Index ranges	-8 ≤ h ≤ 8, -8 ≤ k ≤ 14, -18 ≤ l ≤ 18	-9 ≤ h ≤ 9, -16 ≤ k ≤ 18, -23 ≤ l ≤ 19	-16 ≤ h ≤ 16, -12 ≤ k ≤ 12, -10 ≤ l ≤ 8	-11 ≤ h ≤ 11, -10 ≤ k ≤ 9, -14 ≤ l ≤ 13
Reflections collected	8215	14690	10253	6192
Independent reflections	2322 [R_{int} = 0.0241, R_{sigma} = 0.0203]	3938 [R_{int} = 0.0271, R_{sigma} = 0.0258]	2349 [R_{int} = 0.0731, R_{sigma} = 0.0642]	1822 [R_{int} = 0.0421, R_{sigma} = 0.0374]
Data/restraints/parameters	2322/0/133	3938/0/141	2349/0/165	1822/0/83
Goodness-of-fit on F^2 (a)	1.091	1.049	1.036	0.839
Final R indexes [I>=2σ (I)] (b)	R_1 = 0.0229, wR_2 = 0.0639	R_1 = 0.0225, wR_2 = 0.0517	R_1 = 0.0617, wR_2 = 0.1620	R_1 = 0.0349, wR_2 = 0.0976
Final R indexes [all data] (b)	R_1 = 0.0234, wR_2 = 0.0644	R_1 = 0.0269, wR_2 = 0.0535	R_1 = 0.0651, wR_2 = 0.1656	R_1 = 0.0366, wR_2 = 0.1012
Largest diff. peak/hole [e/Å^{-3}]	0.77/-0.75	0.94/-0.50	2.09/-1.23	1.30/-1.93

(a) GooF = S = $[[\sum w(F_o^2-F_c^2)^2]/(m-n)]^{1/2}$, where m = number of reflexes and n = number of parameters; (b) $R_1 = \sum||F_o|-|F_c||/\sum|F_o|$; $wR_2 = [\sum[w(F_o^2-F_c^2)^2/\sum[(wF_o^2)^2]]^{1/2}$; $w = 1/[\sigma^2(F_o^2)+(aP)^2+bP]$, where $P = (F_o^2+2F_c^2)$

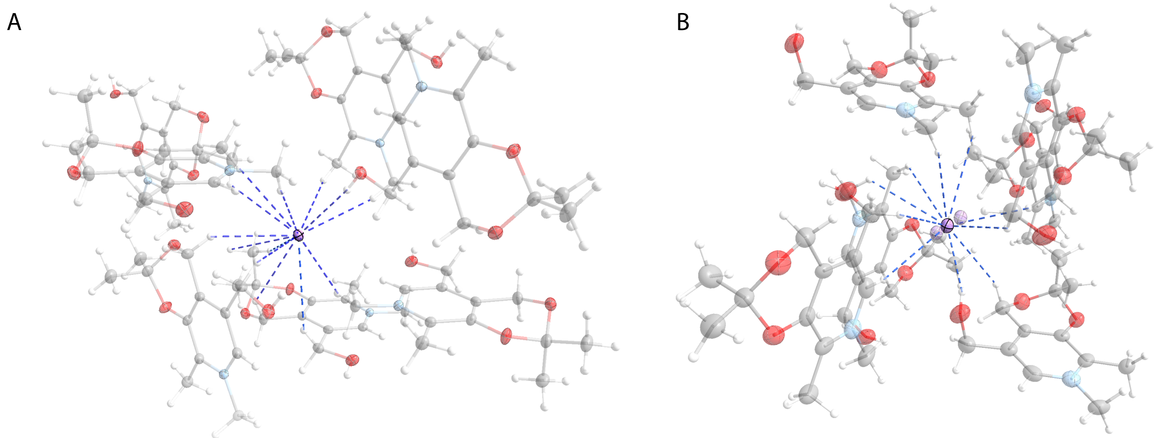

13 H – I stabilizating bonding with 2.75–4.27 Å length *versus* 9 H – I stabilizating bonding with 2.62–3.47 Å length

Figure S4. Fragments of the crystal packing with depicted stabilizing hydrogen bonding network H···I of **1**^{o} (A) and **1**^{m} (B) (displacement ellipsoids are shown at the 50% probability level).

Table S5. Crystal data and structure refinement for **6** at different temperatures (100–300 K).

Identification code	**6_100K**	**6_150K**	**6_200K**	**6_250K**	**6_300K**
Empirical formula	$C_9H_{14}INO_3$				
Formula weight	311.11				
Temperature [K]	100.00(10)	150.02(10)	200.06(12)	250.00(10)	300.2(4)
Crystal system	monoclinic				
Space group	$P2_1/n$				
a [Å]<br>b [Å]<br>c [Å]<br>α [°]<br>β [°]<br>γ [°]	6.62750(10)<br>11.30050(10)<br>14.8296(2)<br>90<br>98.5070(10)<br>90	6.61766(8)<br>11.35259(11)<br>14.86740(17)<br>90<br>98.4485(11)<br>90	6.61682(8)<br>11.40298(12)<br>14.91799(18)<br>90<br>98.3315(11)<br>90	6.61280(10)<br>11.45760(10)<br>14.9724(2)<br>90<br>98.1850(10)<br>90	6.61007(14)<br>11.5090(2)<br>15.0251(3)<br>90<br>98.020(2)<br>90
Volume [Å^3]	1098.43(2)	1104.83(2)	1113.71(2)	1122.86(2)	1131.86(4)
Z	4				
ρ_{calc} [g/cm^3]	1.881	1.870	1.855	1.840	1.826
μ [mm^{-1}]	22.797	22.665	22.485	22.301	22.124
F(000)	608.0				
Crystal size [mm^3]	0.03 × 0.015 × 0.015				
Radiation type	Cu Kα (λ = 1.54184)				
2Θ range for data collection [°]	9.882 to 159.232	9.842 to 159.692	9.802 to 158.964	9.758 to 158.526	9.716 to 159.814
Index ranges	-8 ≤ h ≤ 8, -8 ≤ k ≤ 14, -18 ≤ l ≤ 18	-8 ≤ h ≤ 8, -5 ≤ k ≤ 14, -18 ≤ l ≤ 18	-8 ≤ h ≤ 8, -10 ≤ k ≤ 14, -18 ≤ l ≤ 18	-8 ≤ h ≤ 8, -9 ≤ k ≤ 14, -18 ≤ l ≤ 18	-8 ≤ h ≤ 8, -10 ≤ k ≤ 14, -18 ≤ l ≤ 18
Reflections collected	8215	8311	8047	8489	8739
Independent reflections	2322 [R_{int} = 0.0241, R_{sigma} = 0.0203]	2338 [R_{int} = 0.0250, R_{sigma} = 0.0210]	2348 [R_{int} = 0.0284, R_{sigma} = 0.0238]	2363 [R_{int} = 0.0347, R_{sigma} = 0.0279]	2394 [R_{int} = 0.0449, R_{sigma} = 0.0356]
Data/restraints/ parameters	2322/0/133	2338/0/133	2348/0/133	2363/0/133	2394/0/132
Goodness-of-fit on F^2 [(a)]	1.091	1.084	1.091	1.103	1.094
Final R indexes [I>=2σ (I)] [(b)]	R_1 = 0.0229, wR_2 = 0.0639	R_1 = 0.0251, wR_2 = 0.0699	R_1 = 0.0249, wR_2 = 0.0677	R_1 = 0.0269, wR_2 = 0.0761	R_1 = 0.0372, wR_2 = 0.1090
Final R indexes [all data] [(b)]	R_1 = 0.0234, wR_2 = 0.0644	R_1 = 0.0260, wR_2 = 0.0708	R_1 = 0.0259, wR_2 = 0.0687	R_1 = 0.0280, wR_2 = 0.0771	R_1 = 0.0389, wR_2 = 0.1108
Largest diff. peak/hole [e/Å^{-3}]	0.77/-0.75	1.16/-0.96	1.03/-1.07	0.94/-1.08	1.13/-1.17

[(a)] GooF = S = $[[\sum w(F_o^2-F_c^2)^2]/(m-n)]^{1/2}$, where m = number of reflexes and n = number of parameters; [(b)] $R_1 = \sum||F_o|-|F_c||/\sum|F_o|$; $wR_2 = [\sum[w(F_o^2-F_c^2)^2/\sum[(wF_o^2)^2]]^{1/2}$; $w = 1/[\sigma^2(F_o^2)+(aP)^2+bP]$, where $P = (F_o^2+2F_c^2)/3$

Table S6. Crystal data and structure refinement for **2** at different temperatures (150–291 K).

Identification code	**2_150K**	**2_200K**	**2_257K**	**2_291K**
Empirical formula	$C_{13}H_{20}INO_3$			
Formula weight	365.20	364.19	365.20	365.20
Temperature [K]	150.15	200.00(10)	257.00(10)	290.89(11)
Crystal system	monoclinic			
Space group	Cc			
a [Å]<br>b [Å]<br>c [Å]<br>α [°]<br>β [°]<br>γ [°]	8.59543(9)<br>16.07503(17)<br>10.52397(11)<br>90<br>92.0919(10)<br>90	8.60993(9)<br>16.14795(16)<br>10.54425(10)<br>90<br>91.9287(9)<br>90	8.61484(9)<br>16.27823(16)<br>10.58109(10)<br>90<br>91.6592(9)<br>90	8.60801(10)<br>16.39039(17)<br>10.61080(11)<br>90<br>91.4459(10)<br>90
Volume [Å^3]	1453.15(3)	1465.17(3)	1483.21(2)	1496.59(3)
Z	4			
ρ_{calc} [g/cm^3]	1.669	1.651	1.635	1.621
μ [mm^{-1}]	17.333	17.190	16.981	16.829
F(000)	728.0	724.0	728.0	728.0
Crystal size [mm^3]	0.121 × 0.083 × 0.07			
Radiation type	Cu Kα (λ = 1.54184)			
2Θ range for data collection [°]	11.008 to 159.498	10.958 to 159.008	10.87 to 159.308	10.796 to 158.97
Index ranges	-10 ≤ h ≤ 10, -20 ≤ k ≤ 20, -13 ≤ l ≤ 11	-10 ≤ h ≤ 10, -20 ≤ k ≤ 20, -13 ≤ l ≤ 13	-10 ≤ h ≤ 10, -20 ≤ k ≤ 20, -13 ≤ l ≤ 13	-10 ≤ h ≤ 10, -20 ≤ k ≤ 20, -12 ≤ l ≤ 13
Reflections collected	9169	9495	10199	13010
Independent reflections	2609 [R_{int} = 0.0606, R_{sigma} = 0.0459]	2637 [R_{int} = 0.0588, R_{sigma} = 0.0454]	2651 [R_{int} = 0.0580, R_{sigma} = 0.0429]	2981 [R_{int} = 0.0567, R_{sigma} = 0.0411]
Data/restraints/parameters	2609/2/169	2637/2/169	2651/2/169	2981/2/169
Goodness-of-fit on F^2 (a)	1.104	1.045	1.079	1.127
Final R indexes [I>=2σ (I)] (b)	R_1 = 0.0486, wR_2 = 0.1282	R_1 = 0.0452, wR_2 = 0.1183	R_1 = 0.0317, wR_2 = 0.0846	R_1 = 0.0616, wR_2 = 0.1711
Final R indexes [all data] (b)	R_1 = 0.0488, wR_2 = 0.1286	R_1 = 0.0454, wR_2 = 0.1186	R_1 = 0.0321, wR_2 = 0.0850	R_1 = 0.0620, wR_2 = 0.1718
Largest diff. peak/hole [e/Å^{-3}]	1.33/-1.73	1.29/-1.12	0.83/-0.71	1.09/-0.88
Flack parameter	-0.006(14)	-0.014(13)	-0.013(9)	-0.012(17)

(a) GooF = S = $[[\sum w(F_o^2-F_c^2)^2]/(m-n)]^{1/2}$, where m = number of reflexes and n = number of parameters; (b) $R_1 = \sum||F_o|-|F_c||/\sum|F_o|$; $wR_2 = [\sum[w(F_o^2-F_c^2)^2/\sum[(wF_o^2)^2]]^{1/2}$; $w = 1/[\sigma^2(F_o^2)+(aP)^2+bP]$, where $P = (F_o^2+2F_c^2)/3$

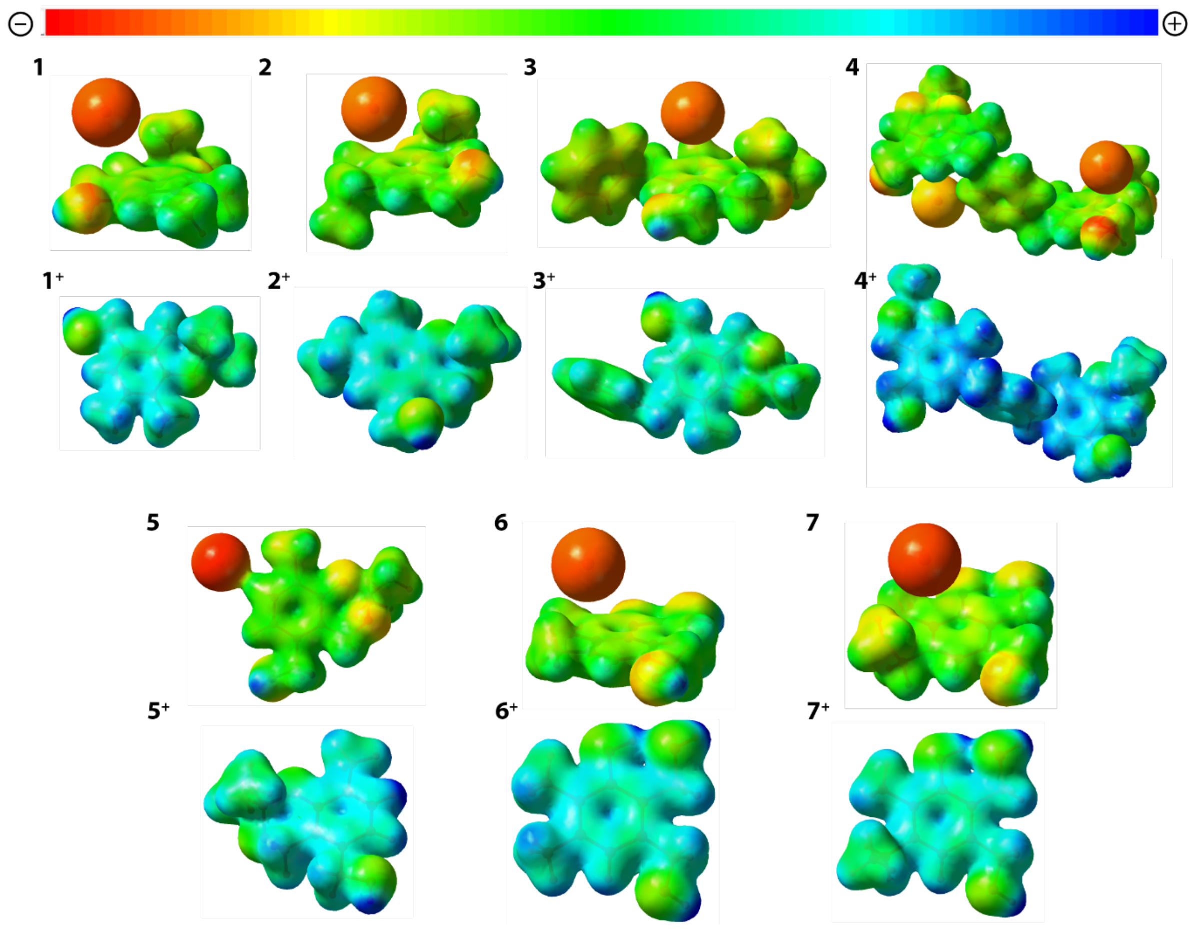

Figure S5. Calculated ESP plots on a 0.02 a.u. isosurface for ionic pairs **1**–**7** and their cations.

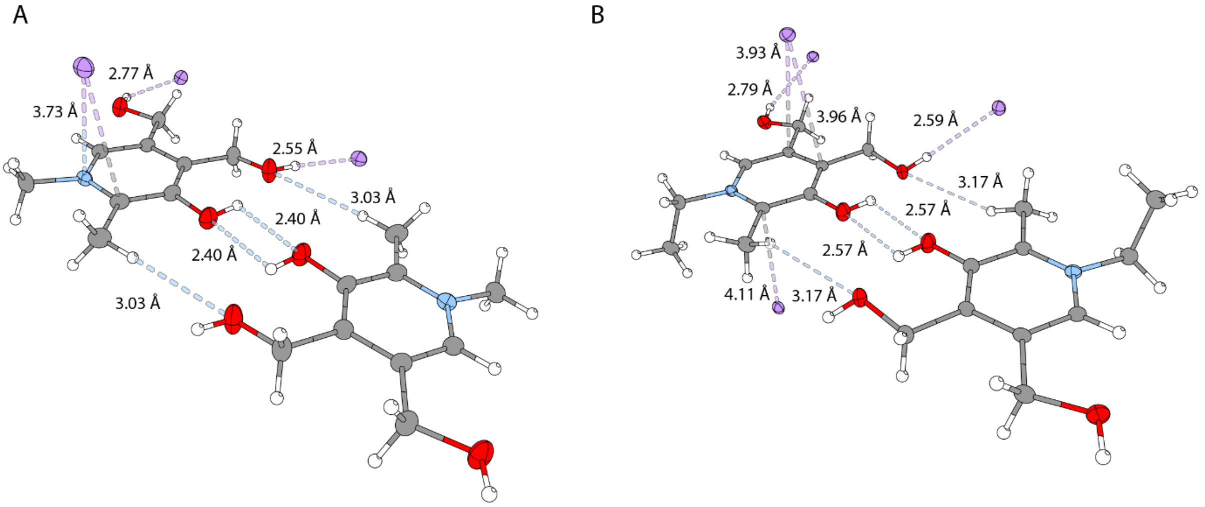

Figure S6. Dimer formation and in plane H···I bonding in **6** (A) and **7** (B), (displacement ellipsoids are shown at the 50% probability level).

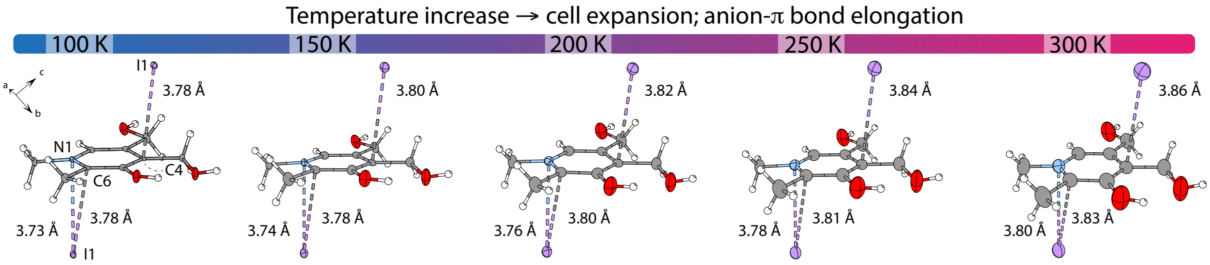

T, K	N1···I1, Å	C6···I1, Å	C4···I1, Å
100	3.7301(20)	3.7750(23)	3.7792(23)
150	3.7434(20)	3.7837(24)	3.7948(24)
200	3.7639(19)	3.7987(21)	3.8164(23)
250	3.7795(22)	3.8099(25)	3.8377(25)
300	3.7967(24)	3.8312(30)	3.8630(32)

Figure S7. Molecular view of the **6** with selected shortest I···E (E = C or N) anion-π distances (displacement ellipsoids are shown at the 50% probability level). Shortest anion-π distances I···E(E = C or N) in the crystal packing of **6** at variable temperatures.

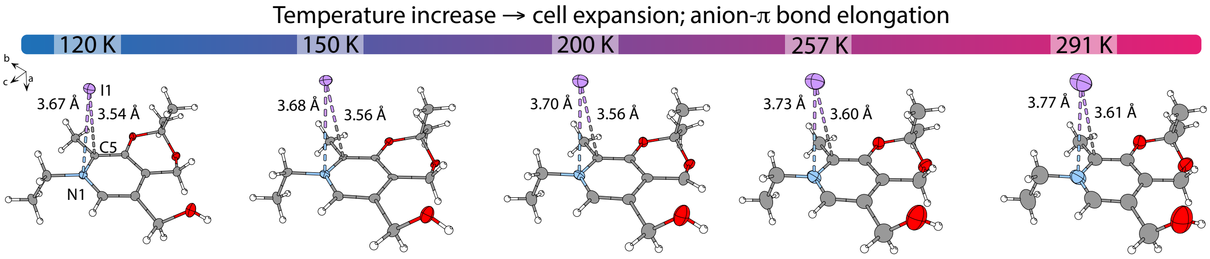

T, K	N1···I1, Å	C5···I1, Å
120	3.6676(29)	3.5423(32)
150	3.6793(64)	3.5551(82)
200	3.6963(60)	3.5615(79)
257	3.7327(46)	3.596(5)
291	3.7666(93)	3.6094(111)

Figure S8. Molecular view of the **2** with selected shortest I···E (E = C or N) anion-π distances (displacement ellipsoids are shown at the 50% probability level). Shortest anion-π distances I···E(E = C or N) in the crystal packing of **2** at variable temperatures.

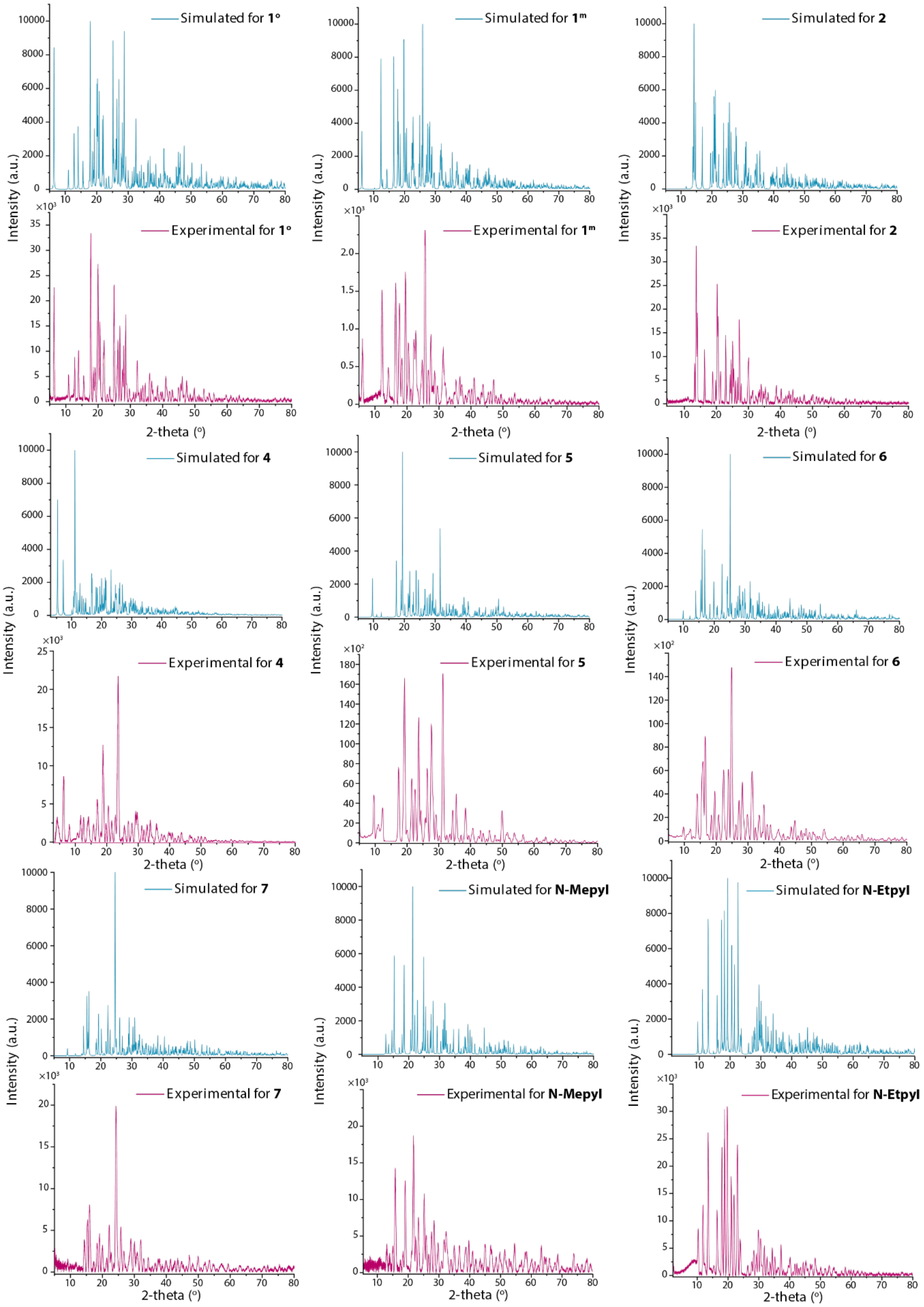

Figure S9. Simulated and experimental PXRD patterns for **1°**, **1ᵐ**, **2–7**, **N-Mepyl** and **N-Etpyl**.

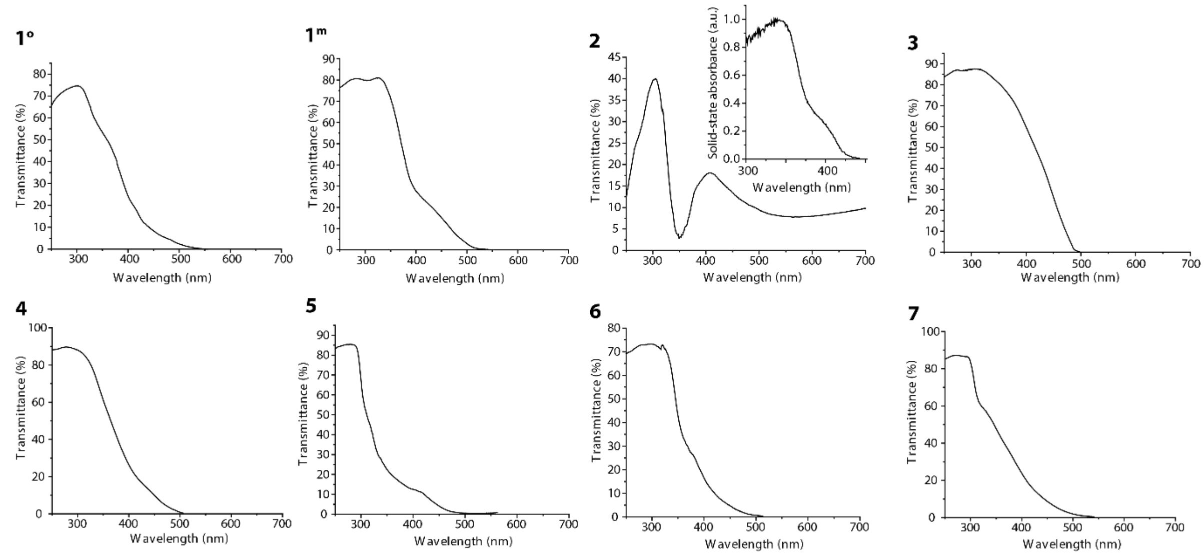

Figure S10. Solid-state reflectance spectra of **1**^{o}, **2**–**7** and **1**^{m} (inset for **2** shows the solid-state absorption spectrum measured with integrating sphere via synchronous scan). Due to the intense phosphorescence of **2**, the reflectance spectrum in high energy region (250-400 nm) is affected by emission photons.

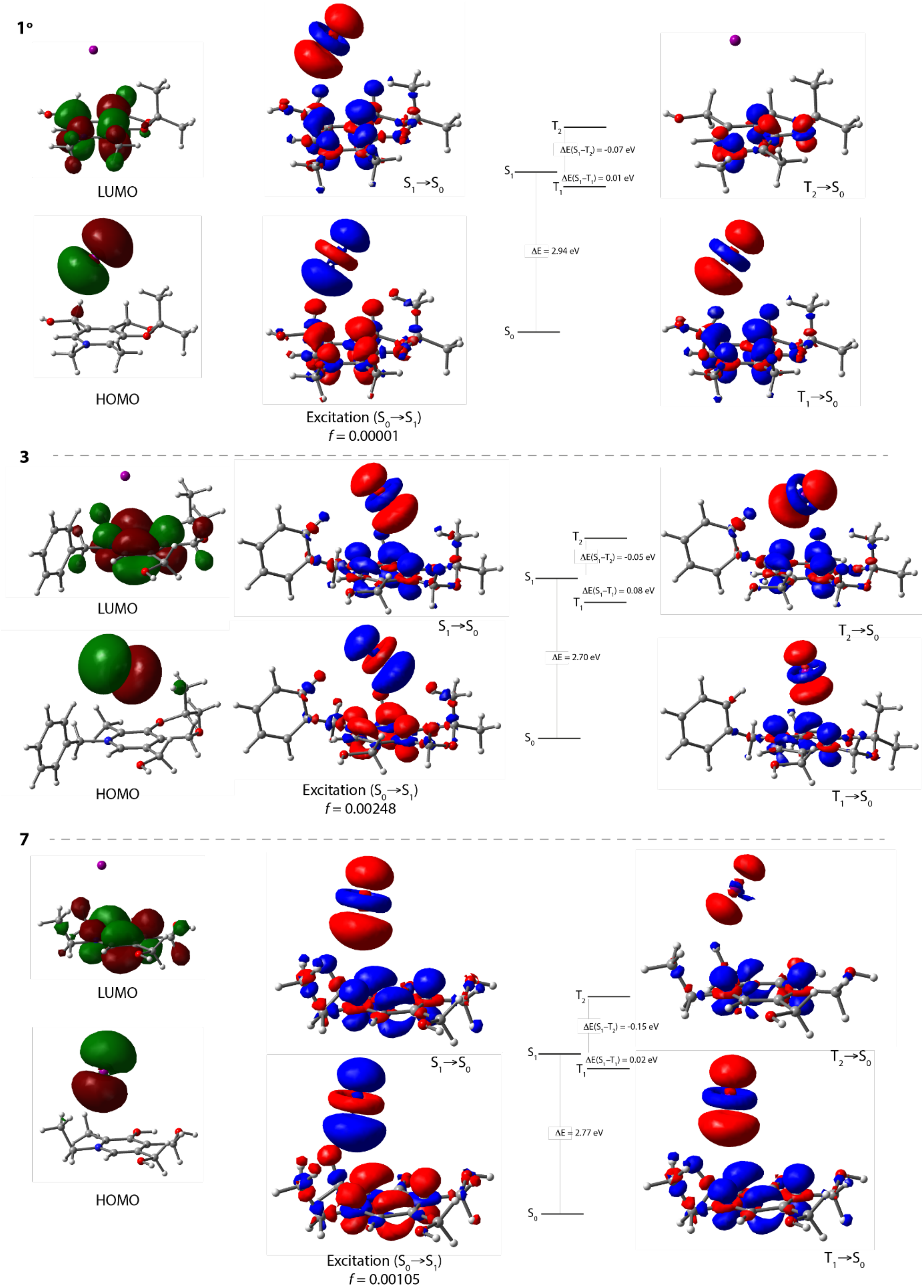

Figure S11. HOMO/LUMO levels; excitation ($S_0 \rightarrow S_1$) and triplet emission (T_2, $T_1 \rightarrow S_0$) electron density difference plots for **1°**, **3** and **7** (isovalue of 0.04 a.u., loss electron density: blue, gain: red).

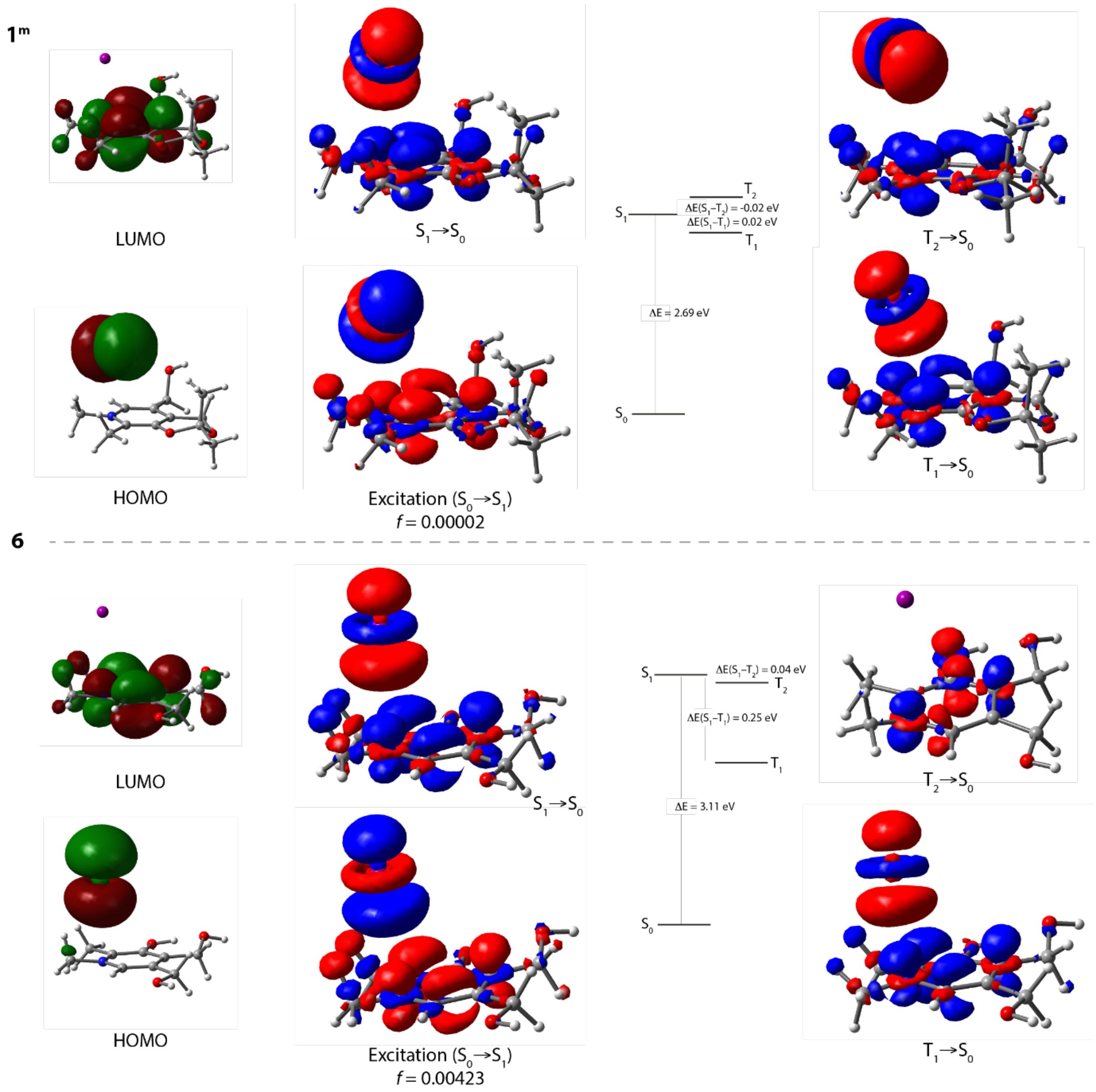

Figure S12. HOMO/LUMO levels; excitation ($S_0 \rightarrow S_1$) and triplet emission (T_2, $T_1 \rightarrow S_0$) electron density difference plots for **1ᵐ** and **6** (isovalue of 0.04 a.u., loss electron density: blue, gain: red).

Table S7. Summary of the photophysical properties of pyridinium salts (**1°**, **3**, **4**, **7**, **N-Mepyl** and **N-Etpyl**) in the solid state measured at 77 K.

	λ_{exc} [nm]	λ_{em} [nm]	τ_i (A_i) [μs]	τ_{av} [μs] [a]	Character	(I^-)–π [Å]
**1°**	277, 340	410^{sh}, 440	4.89 (69), 16.46 (29), 119.41 (2), 851.60 (<0.01) @440<br>6.72 (70), 15.87 (28), 58.65 (2), 190.13 (<0.01) @570	14.32@440<br>10.88@570	^{3}LE + ^{3}CT	4.09
**3**	330, 390^{sh}	545	0.03 (54), 0.49 (23), 2.65 (19), 8.62 (3)	0.97	^{3}CT	3.75
**4**	320	510	9.57 (61), 17.04 (39), 98.95 (1)	12.69	^{3}CT + ^{3}LE	3.86/3.87
**7**	315	480	21.81 (72), 214.90 (18), 1749.96 (8), 5984.98 (2)@440<br>22.99 (83), 176.20 (12), 1525.95 (4), 5925.39 (1)@480<br>25.08 (94), 411.26 (5), 4284.21 (1) @540<br>13.20 (87), 82.09 (12), 816.77 (1), 5859.18 (<0.01) @600	335.22@440<br>176.14@480<br>84.81@540<br>51.12@600	^{3}LE + ^{3}CT	3.93/4.10
**N-Mepyl**	372	540	1.30 (58), 3.86 (42)	2.38	^{3}CT	3.76/4.11
**N-Etpyl**	358	520	5.02	–	^{3}CT	3.71/4.03

[a] – average amplitude-weighted emission lifetime for multiexponential decays ($\tau_{av} = \Sigma A^i \tau^i$, where A^i – weight of the exponent.)

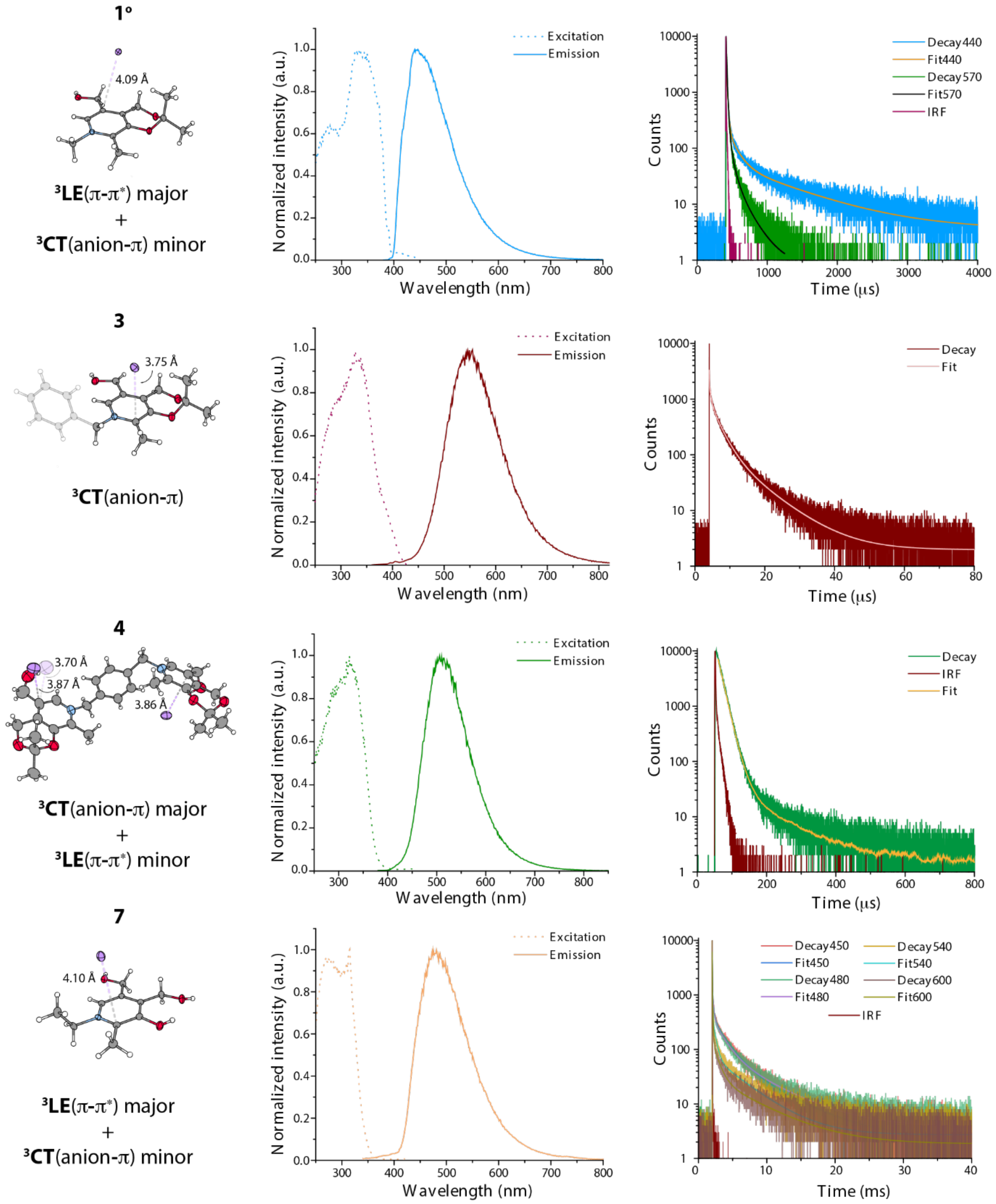

Figure S13. Excitation (dotted), emission (solid), and time-resolved spectra of the **1°**, **3**, **4** and **7** measured at 77 K.

Table S8. Summary of the photophysical properties of pyridinium salts (**1**^{m}, **2** and **6**).

	T [K]	λ_{exc} [nm]	λ_{em} [nm]	τ_i (A_i) [μs]	τ_{av} [μs][a]	Q.Y.	k_r [b] [$\times 10^4 s^{-1}$]	k_{nr} [b] [$\times 10^4 s^{-1}$]
**1**^{m}	298	330	525	0.33 (34), 0.63 (66)	0.53	0.07	13.2	175.5
	250	330	522	2.57 (58), 3.75 (42)	3.07			
	190	330	507	3.95 (23), 8.03 (77)	7.10			
	130	333	498	4.33 (6), 16.55 (94)	14.30			
	77	335	478	4.82 (34), 13.79 (66)$^{@440}$<br>8.40 (13), 13.98 (86)$^{@480}$<br>3.2 (12), 13.44 (88)$^{@555}$	10.75$^{@440}$<br>13.23$^{@480}$<br>12.24$^{@550}$			
**2**	317	365	535	9.39 (95), 15.76 (5)	9.73			
	298	365	532	9.93 (96), 16.92 (4)	10.21	0.93	9.1	0.7
	250	360	527	11.58 (99), 25.35 (1)	11.70		8.6 [c]	
	200	355	522	11.04 (79), 15.10 (21)	11.88		8.4 [c]	
	150	350	520	11.29 (94), 18.23 (6)	11.73		8.5 [c]	
	110	350	520	10.93 (82), 15.63 (18)	11.78		8.5 [c]	
	77	350	520	11.12 (85), 16.57 (15)	11.91		8.4 [c]	
	7	350	520	11.79	–		8.5 [c]	
**6**	298	330	565	2.33 (84), 4.90 (16)	2.75	0.03	1.1	35.3
	250	327	532	4.90 (14), 32.38 (79), 304.87 (7)	5.45			
	200	324	512	22.86 (91), 73.42 (8), 629.69 (1)	29.08			
	150	322	488	16.69 (72), 95.89 (18), 255.95 (9), 1063.84 (1)$^{@480}$<br>13.55 (84), 64.66 (10), 212.69 (5), 1089.89 (1)$^{@560}$<br>11.05 (80), 42.48 (14), 185.68 (5), 1000.00 (1)$^{@620}$	58.84$^{@480}$<br>32.20$^{@560}$<br>27.77$^{@620}$			
	77	320	470, 575^{sh}	17.37 (77), 87.24 (15), 524.15 (4), 1328.21 (4)$^{@470}$<br>13.87 (89), 53.68 (8), 276.41 (2), 1363.51 (1)$^{@570}$<br>11.82 (87), 42.60 (11), 271.20 (2), 1566.65 (<0.01)$^{@620}$	104.81$^{@470}$<br>28.72$^{@570}$<br>25.02$^{@620}$			

[a] Average amplitude-weighted emission lifetime for multiexponential decays ($\tau_{av} = \Sigma A^i \tau^i$, where A^i – the weight of the exponent). [b] Calculated from amplitude average lifetimes $k_r = Q.Y/\tau_{av}$; $k_{nr} = (1\text{-}Q.Y)/\tau_{av}$. [c] Calculated with the assumption of Q.Y. equal to 1.0.

Table S9. Calculated oscillator strengths (f), transition energies (E), radiative rate constants (k_r) of phosphorescence, energy differences between the first excited states ($\Delta E(S_1–T_1)/\Delta E(S_1–T_2)$), spin-orbit coupling matrix elements ($<S_1|H_{SO}|T_1>$) between singlet and triplet states and intersystem crossing rates (k_{ISC}) for **1°–3**, **1m**, **6** and **7**.

Sample<br>Anion-π (Å)	**1°**<br>4.09	**1m**<br>3.62	**2**<br>3.54	**3**<br>3.75	**6**<br>3.73	**7**<br>3.93
Excitation $S_0 \rightarrow S_1$						
f	0.00001	0.00002	0.00248	0.00254	0.00423	0.00105
E (eV)	2.94	2.69	3.24	2.70	3.11	2.77
λ (nm)	422	461	382	460	399	448
Character	CT					
Phosphorescence $T_1 \rightarrow S_0$						
$\Delta E(S_1-T_1)$ (eV)	0.01	0.02	0.37	0.08	0.02	0.25
E (eV)	2.15	2.11	2.49	1.92	2.48	2.21
λ (nm)	578	587	497	645	500	561
$\lambda_{Em, exp, max}$ (nm)	440	525	532	545	565	480
k_r (s^{-1}) [a]	7.56×10^{2}	1.52×10^{3}	5.52×10^{3}	5.36×10^{2}	3.12×10^{4}	1.6×10^{3}
k_r (s^{-1}) [b]	1.82×10^{6}	2.98×10^{7}	1.04×10^{7}	5.12×10^{6}	1.29×10^{7}	1.30×10^{6}
$<S_1\|\boldsymbol{H_{SO}}\|T_1>$ (cm^{-1}) [c]	116	648	964	212	449	95
k_{ISC} (s^{-1})	7.05×10^{11}	7.35×10^{8}	– [d]	6.74×10^{9}	3.00×10^{9}	1.21×10^{10}
Character	CT					
Phosphorescence $T_2 \rightarrow S_0$						
$\Delta E(S_1-T_2)$ (eV)	-0.07	-0.02	-0.02	-0.05	0.04	-0.15
$\Delta E(T_1-T_2)$ (eV)	0.08	0.04	0.38	0.13	0.21	0.16
$<S_1\|\boldsymbol{H_{SO}}\|T_2>$ (cm^{-1}) [c]	–	–	1792	–	1782	–
Character	π-π*	CT	CT	CT	π-π*	π-π*/CT
k_{ISC} (s^{-1})	–	–	6.21×10^{12}	–	–	–

[a] – calculated using a Fermi Golden Rule approach; [b] – calculated with the Einstein coefficient for spontaneous emission; [c] – obtained at the S_1 geometry; [d] – not obtained due to breakdown of the harmonic oscillator approximation.

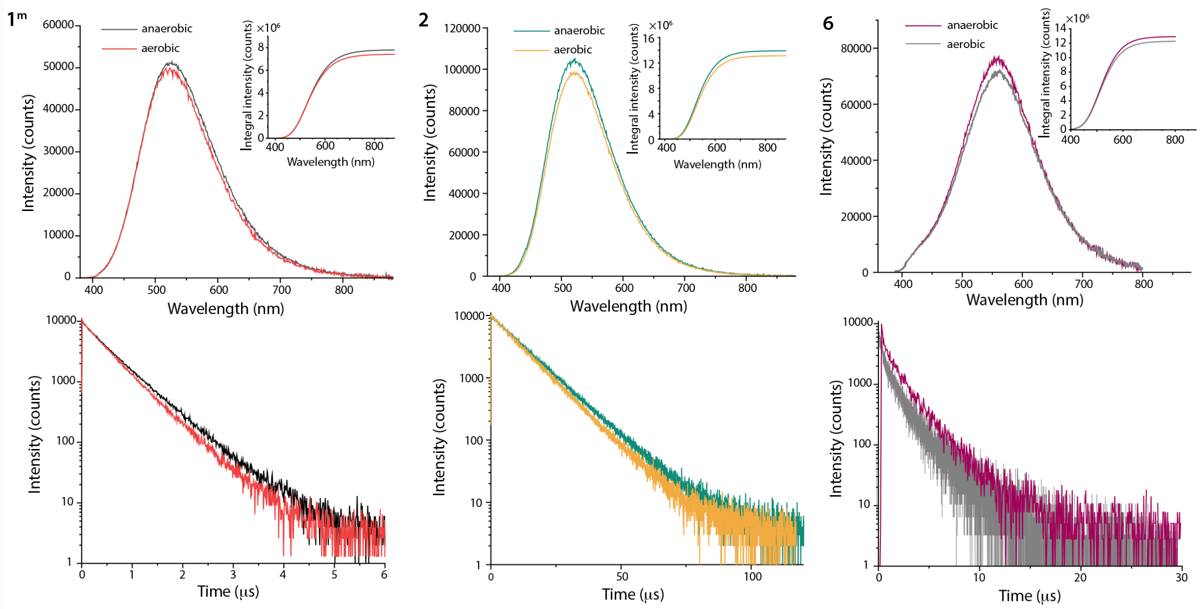

Figure S14. Photoluminescence and lifetime of the excited state of **1^{m}**, **2,** and **6** measured under aerobic and anaerobic conditions.

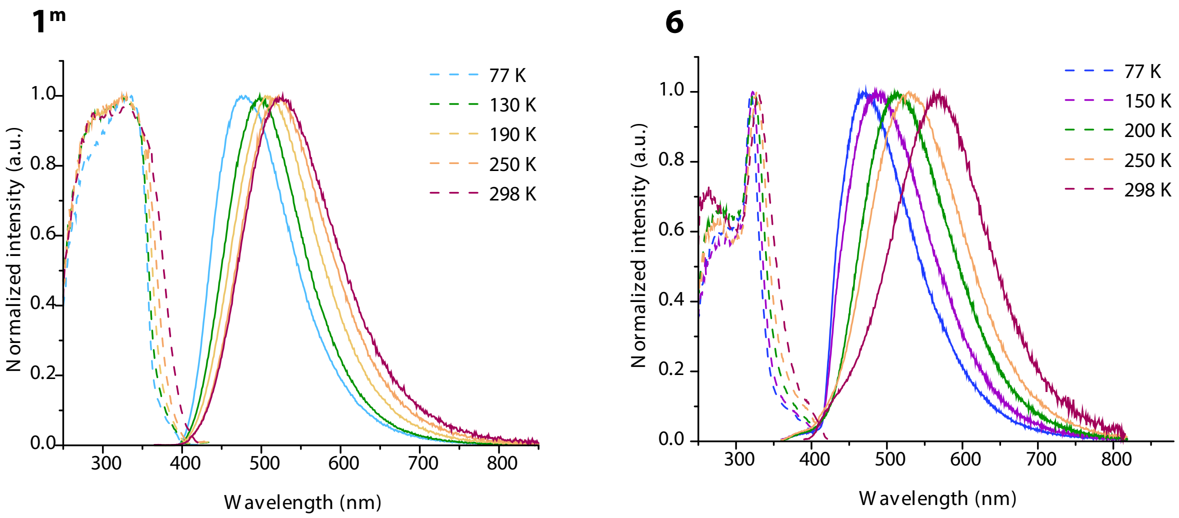

Figure S15. Variable temperature excitation (dashed line), emission (solid line) profiles of solid samples **1ᵐ** and **6**.

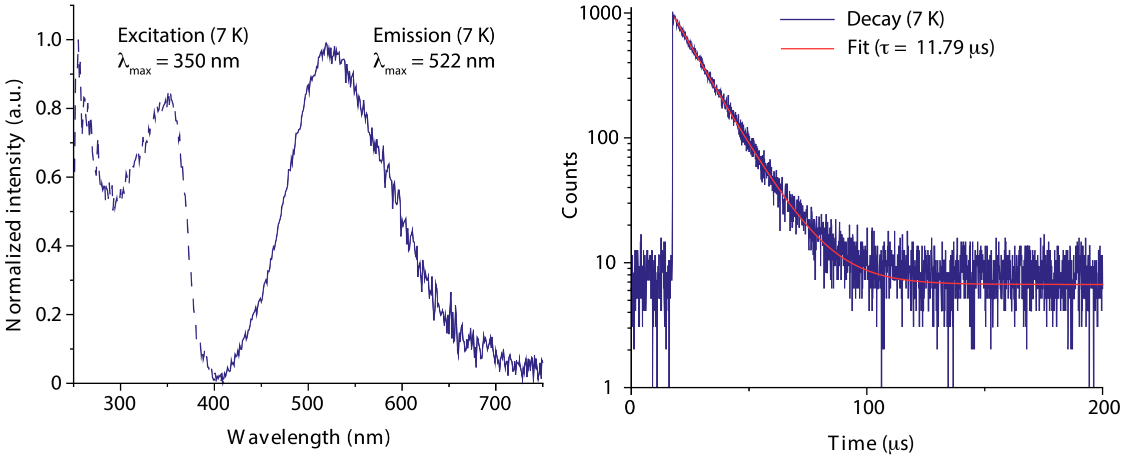

Figure S16. Excitation (dashed line), emission (solid line), emission decay profile (blue), and fit (red) of solid sample **2** measured at 7 K.

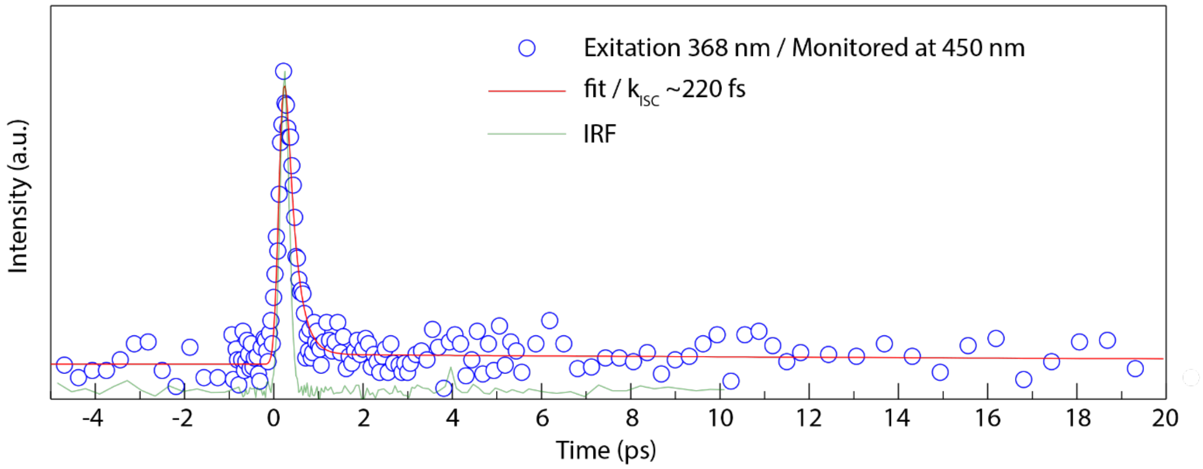

Figure S17. Early relaxation dynamics of crystalline **2** (monitored at 450 nm) obtained by femtosecond emission up-conversion.

Table S10. Distance-dependent calculated radiative rates for **1°** and **2** using a Fermi Golden Rule approach.[7]

Variable anion-π distance

Distance vs phosphorescence rate ($T_1 \rightarrow S_0$)

**1°**		**2**	
distance (Å)	k_r×10^{-3} (s^{-1})	distance (Å)	k_r×10^{-3} (s^{-1})
3.13	1.56	**Optimized T_1**	5.52
3.23	1.58	3.41	5.31
3.34	1.46	3.53	5.16
3.45	1.27	3.64	4.85
3.56	1.32	3.77	4.84
3.67	1.21	3.89	4.79
3.78	1.13	4.00	4.50
3.88	1.03	4.22	4.28
3.98	0.97		
4.09	0.86		
**Optimized T_1**	0.76		

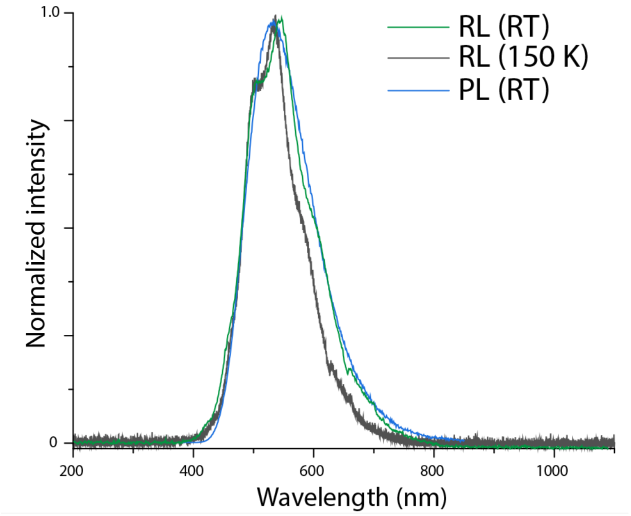

Figure S18. Radioluminescence (RL) spectra of solid sample **2** upon CuKα (λ = 1.54184 Å) irradiation at RT and 150 K, and photoluminescence (PL) spectra measured upon excitation at 365 nm at RT.

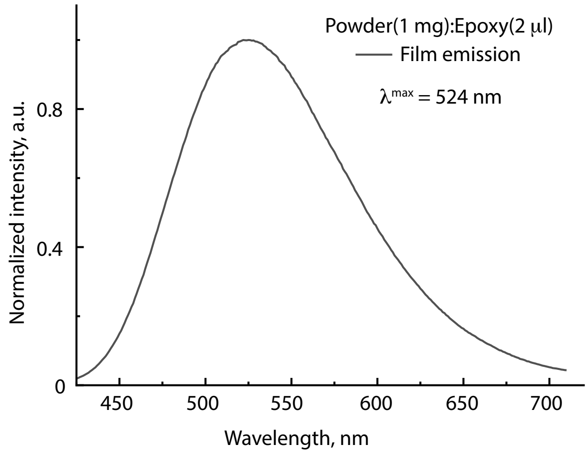

Figure S19. Emission spectrum of the composite film (1 mg of **2** mixed with 2 μl of epoxy resin) upon excitation with 380 nm.

Table S11. Computed spin-orbit coupling matrix elements, energy differences between the lowest excited triplet state (T_1) and 10 lowest lying singlet excited states (S_n), and oscillator strengths (*f*) for the spin-allowed singlet-singlet transitions for **1°**, **1ᵐ**, and **2**. Calculated at the optimized T_1 geometries.

1°

S_n	SOCME(S_n-T_1) [cm^{-1}]	*f* ($S_n \rightarrow S_0$)	ΔE(S_n,T_1) [eV]
1	128	0,00060	0,01
2	2113	0,00008	0,10
3	2103	0,00075	0,18
4	5	0,00466	1,58
5	2	0,00065	1,68
6	4	0,00256	1,76
7	16	0,13617	2,16
8	28	0,01639	2,42
9	52	0,02503	2,53
10	20	0,00747	3,28

1^m

S_n	SOCME(S_n-T_1) [cm^{-1}]	*f* ($S_n \rightarrow S_0$)	ΔE(S_n,T_1) [eV]
1	958	0,00018	0,05
2	1859	0,00670	0,11
3	2047	0,00070	0,29
4	11	0,00084	1,53
5	11	0,00104	1,58
6	16	0,00382	1,78
7	74	0,12776	2,17
8	19	0,00703	2,48
9	13	0,01963	2,58
10	21	0,00641	3,22

2

S_n	SOCME(S_n-T_1) [cm^{-1}]	f ($S_n \rightarrow S_0$)	ΔE(S_n,T_1) [eV]
1	533	0,03445	0,51
2	1410	0,00106	0,59
3	1311	0,01757	0,63
4	35	0,04637	1,88
5	17	0,08103	2,00
6	29	0,00379	2,07
7	28	0,02166	2,11
8	25	0,00176	2,58
9	15	0,02729	2,60
10	5	0,00253	2,74

Table S12. Computed spin-orbit coupling matrix elements, energy differences between the lowest excited triplet state (T_1) and 10 lowest lying singlet excited states (S_n) and oscillator strengths (*f*) for the spin-allowed singlet-singlet transitions for **3**, **6**, and **7**. Calculated at the optimized T_1 geometries.

3

S_n	SOCME(S_n-T_1) [cm^{-1}]	*f* ($S_n \rightarrow S_0$)	ΔE(S_n,T_1) [eV]
1	290	0,00704	0,21
2	1882	0,00053	0,32
3	1878	0,00618	0,50
4	22	0,00220	1,92
5	18	0,00395	2,05
6	11	0,00064	2,15
7	24	0,03893	2,19
8	22	0,00565	2,23
9	9	0,00497	2,24
10	26	0,03401	2,37

6

S_n	SOCME(S_n-T_1) [cm^{-1}]	*f* ($S_n \rightarrow S_0$)	ΔE(S_n,T_1) [eV]
1	233	0,01888	0,46
2	748	0,00158	0,55
3	767	0,00084	0,72
4	14	0,18057	1,82
5	5	0,00387	2,22
6	11	0,00216	2,35
7	10	0,01071	2,52
8	12	0,00911	2,53
9	12	0,00946	3,01
10	11	0,01128	3,39

7

S_n	SOCME(S_n-T_1) [cm^{-1}]	*f* ($S_n \rightarrow S_0$)	ΔE(S_n,T_1) [eV]
1	193	0,00287	0,03
2	2094	0,00108	0,15
3	2078	0,00124	0,34
4	11	0,00054	1,74
5	5	0,00020	1,87
6	10	0,00451	2,05
7	54	0,16194	2,15
8	25	0,03568	2,68
9	14	0,03937	3,04
10	2	0,01380	3,35

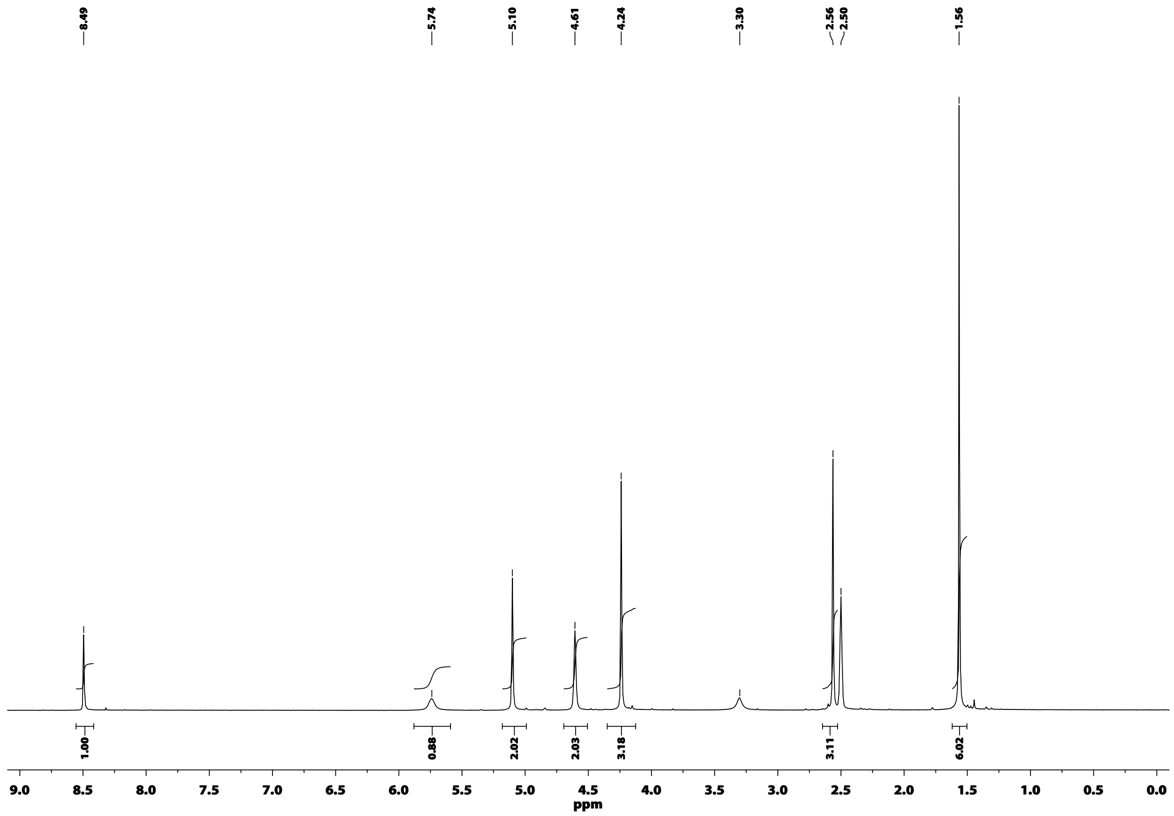

Figure S20. ^{1}H NMR spectra of the pyridinium salt **1** (300 MHz, DMSO-*d*$_6$, 298K).

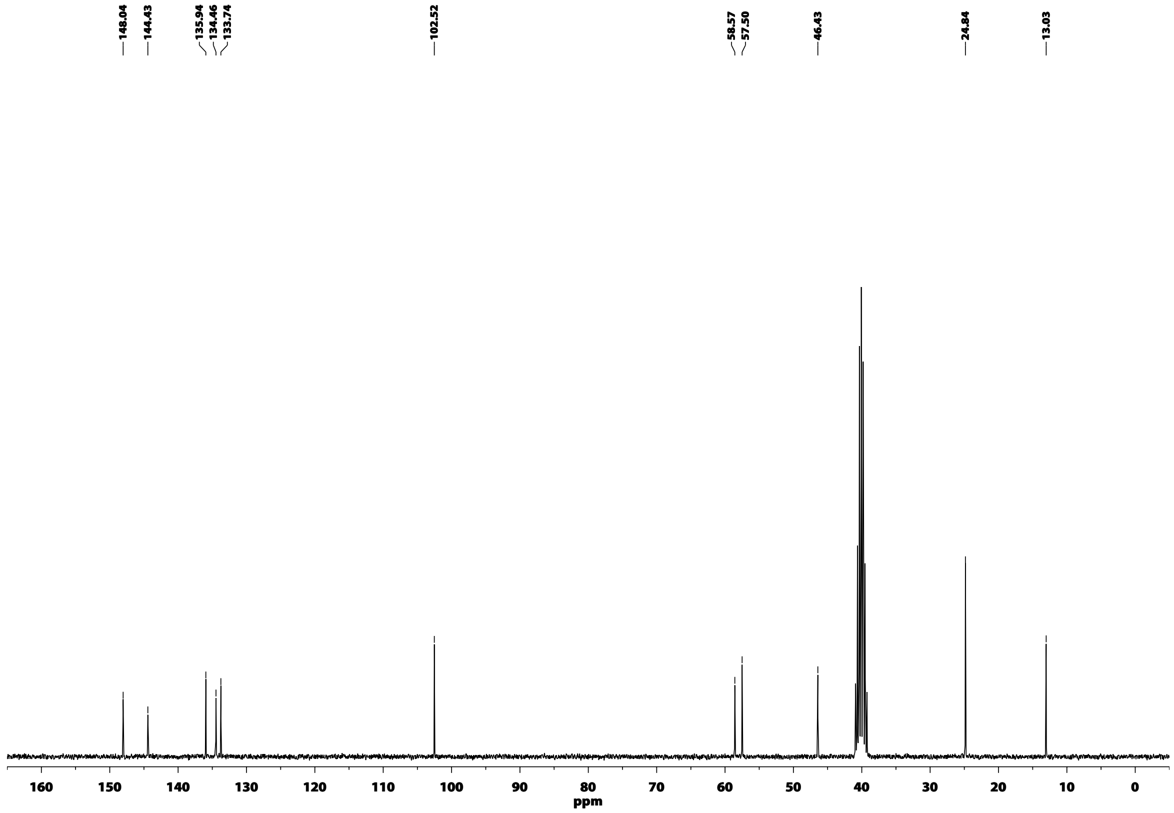

Figure S21. ^{13}C NMR spectra of the pyridinium salt **1** (75 MHz, DMSO-*d*$_6$, 298K).

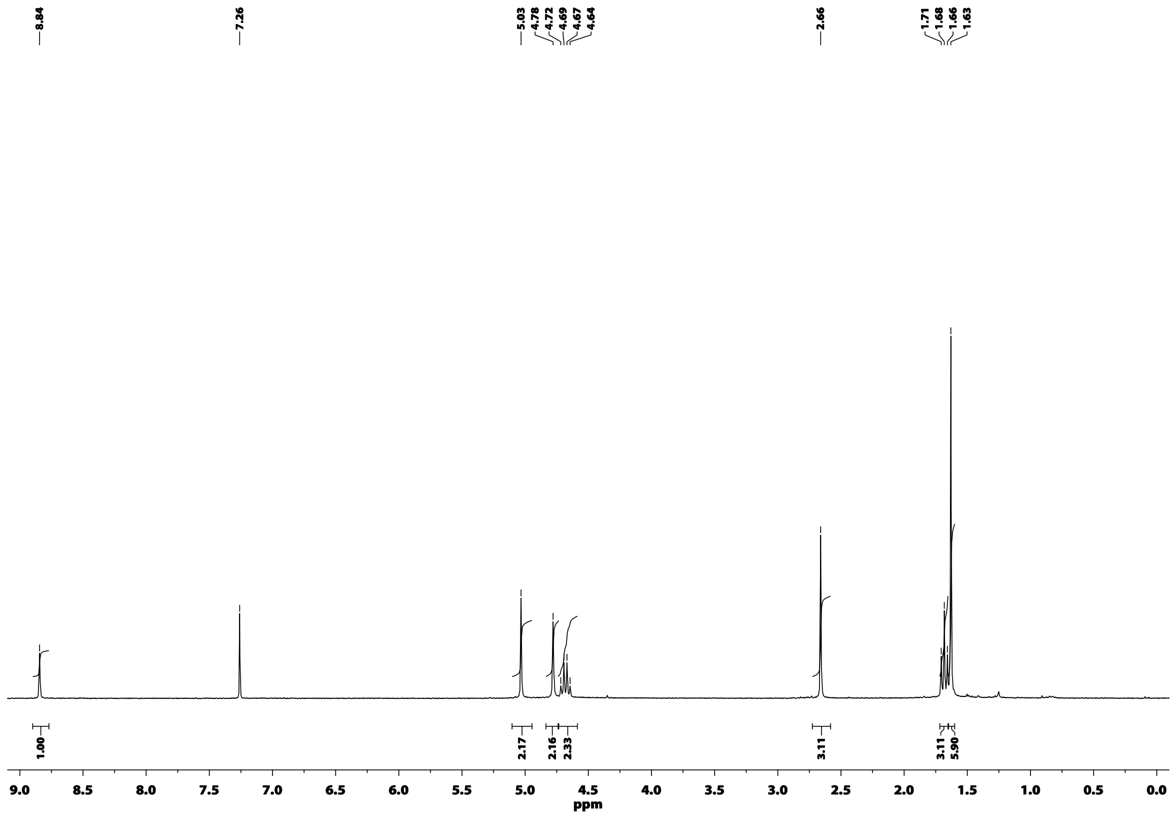

Figure S22. ^{1}H NMR spectra of the pyridinium salt **2** (300 MHz, $CDCl_3$, 298K).

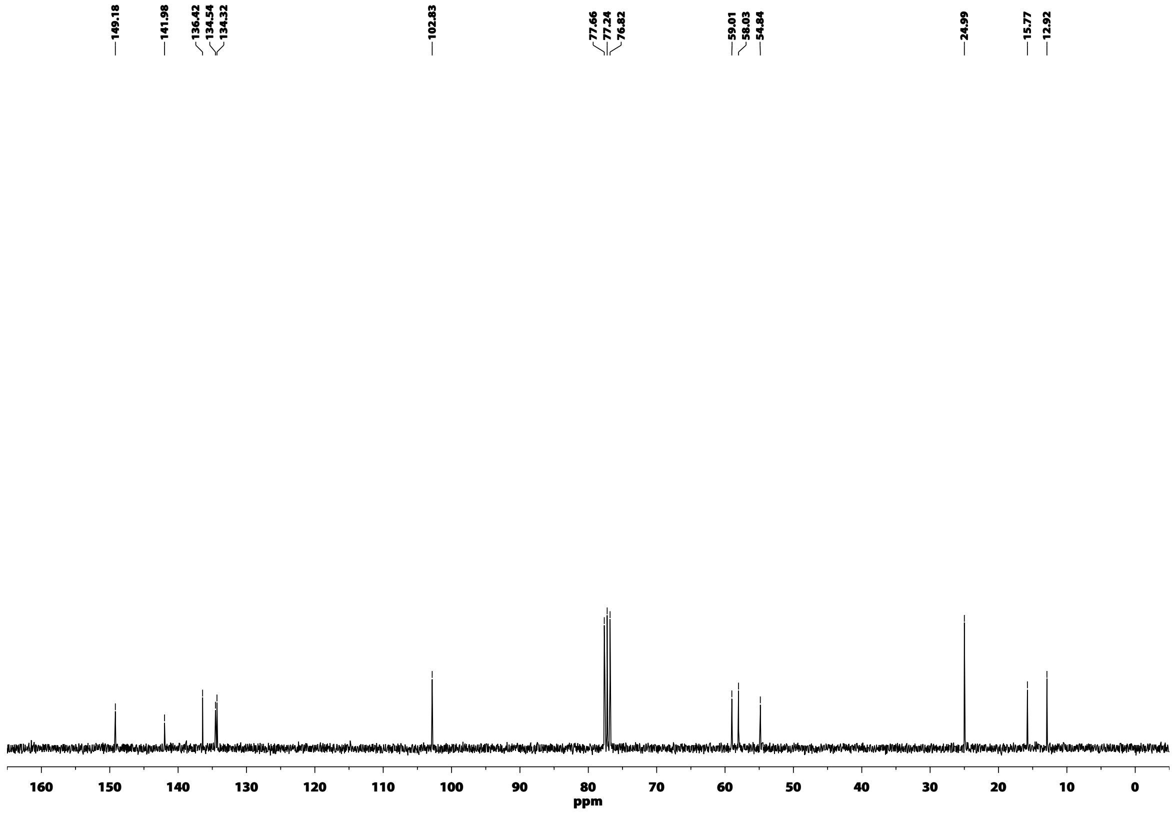

Figure S23. ^{13}C NMR spectra of the pyridinium salt **2** (75 MHz, $CDCl_3$, 298K).

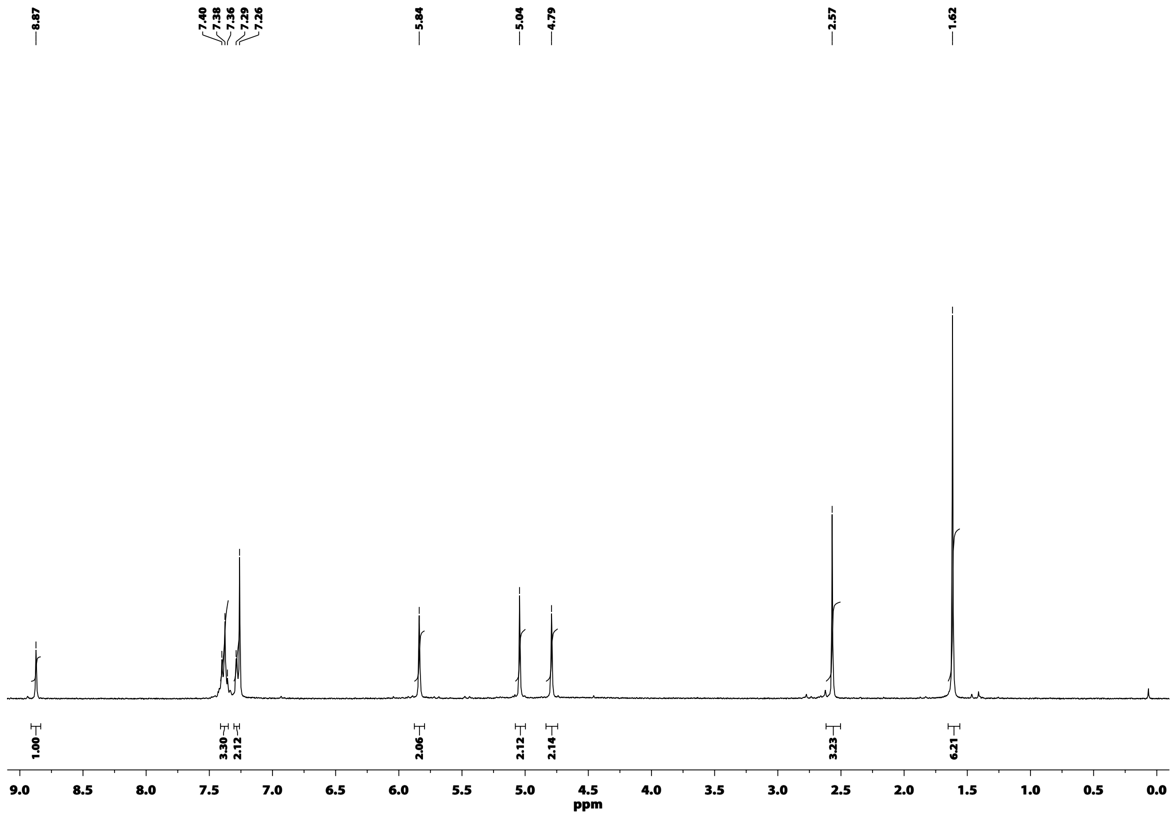

Figure S24. ^{1}H NMR spectra of the pyridinium salt **3** (300 MHz, $CDCl_3$, 298K).

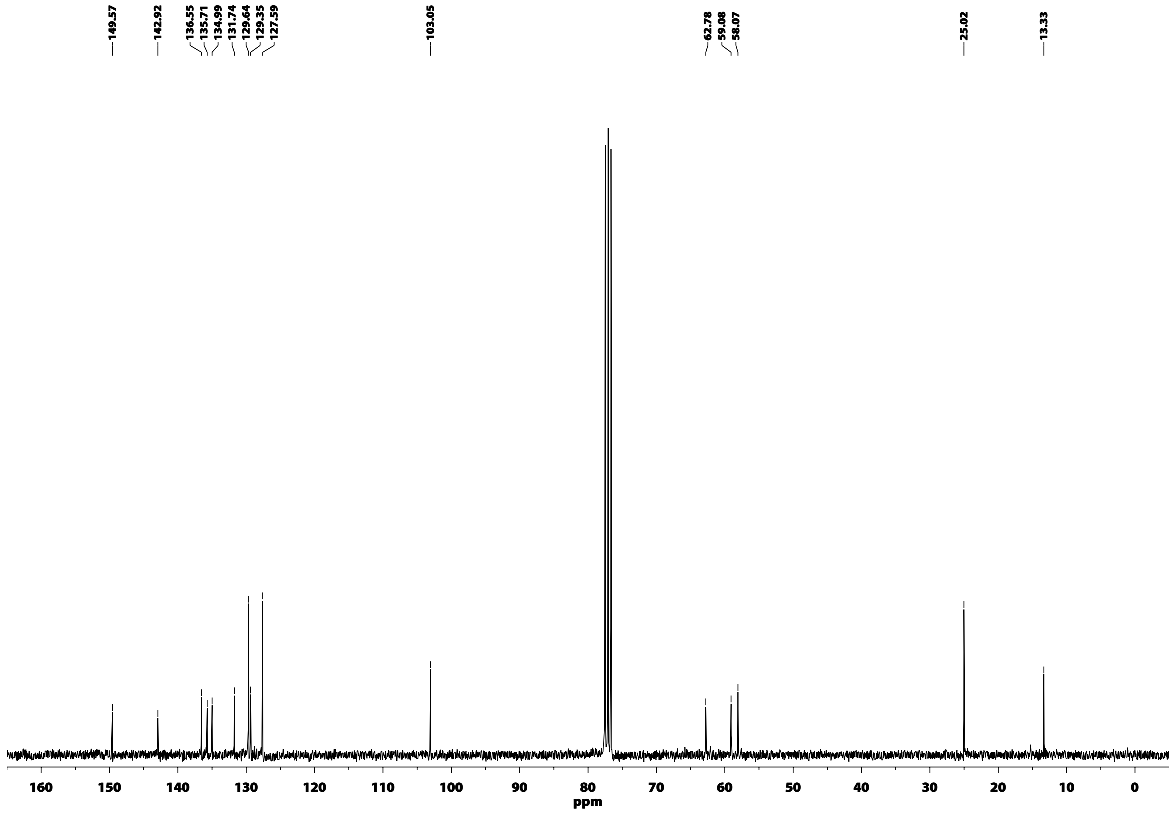

Figure S25. ^{13}C NMR spectra of the pyridinium salt **3** (75 MHz, $CDCl_3$, 298K).

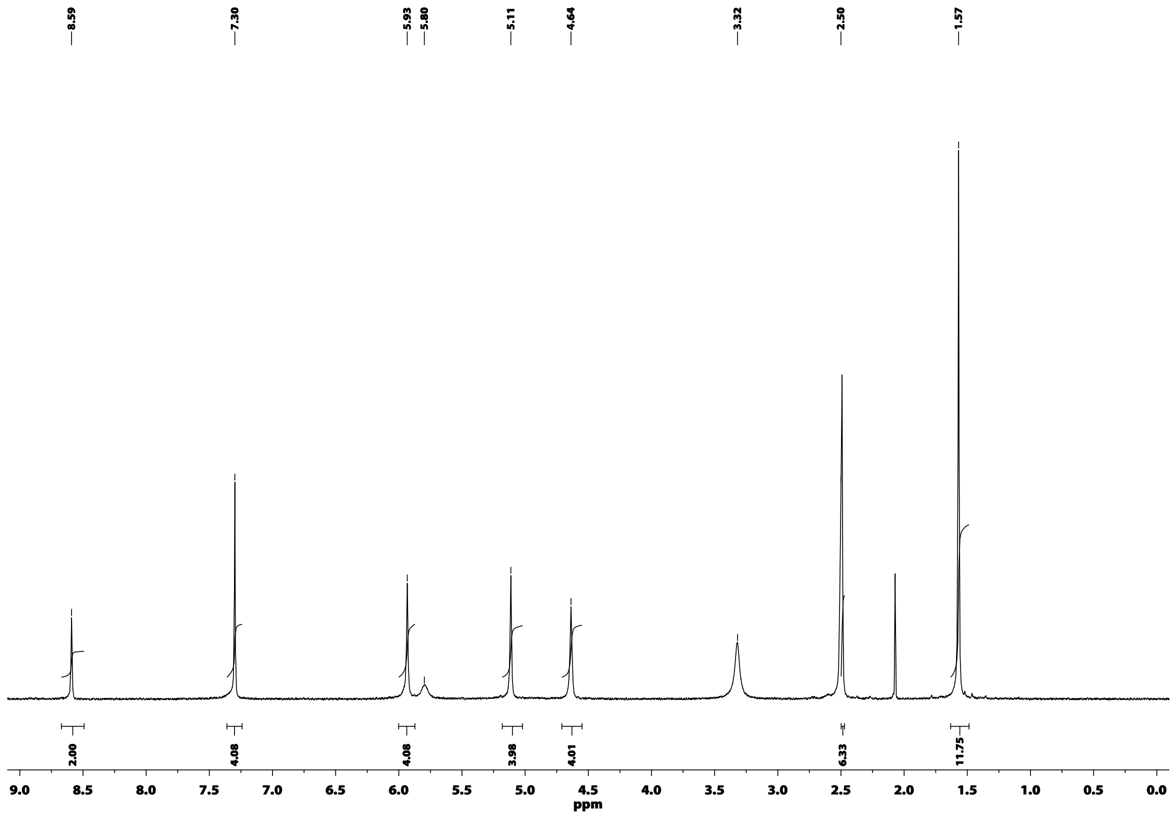

Figure S26. ^{1}H NMR spectra of the pyridinium salt **4** (500 MHz, DMSO-d_6, 298K).

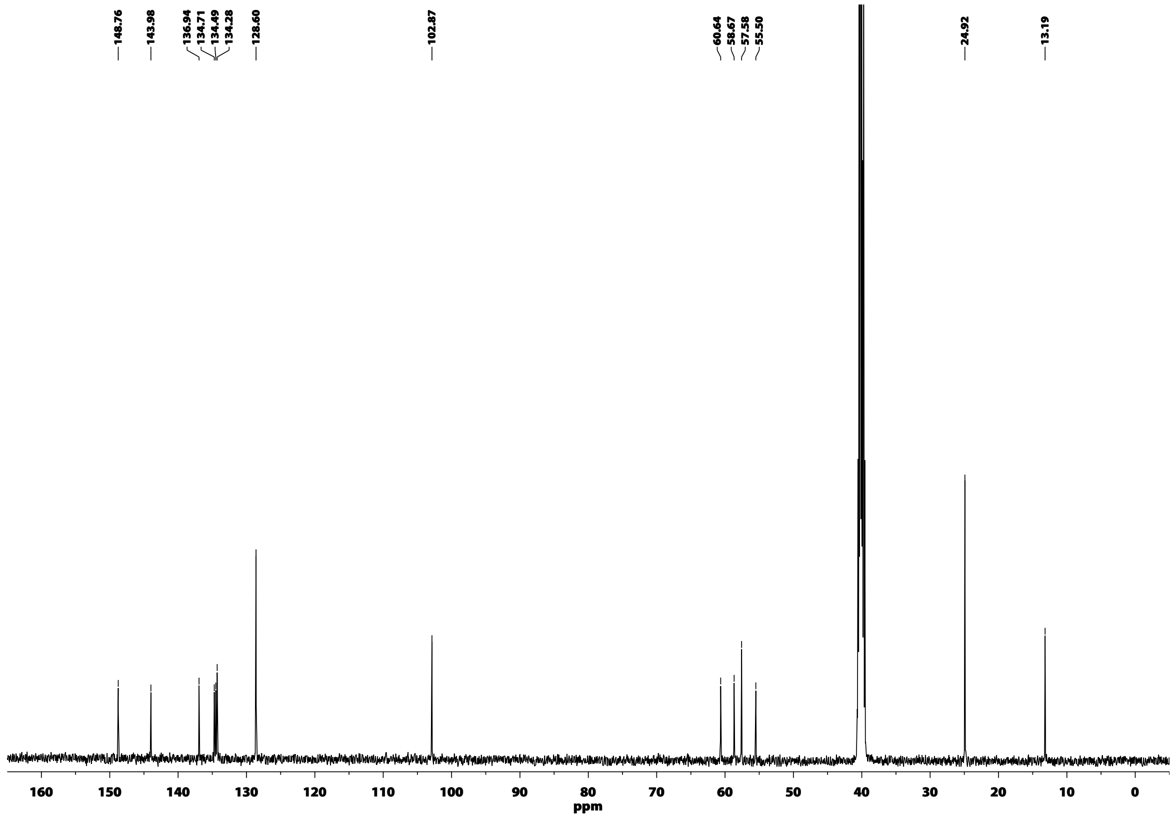

Figure S27. ^{13}C NMR spectra of the pyridinium salt **4** (125 MHz, DMSO-d_6, 298K).

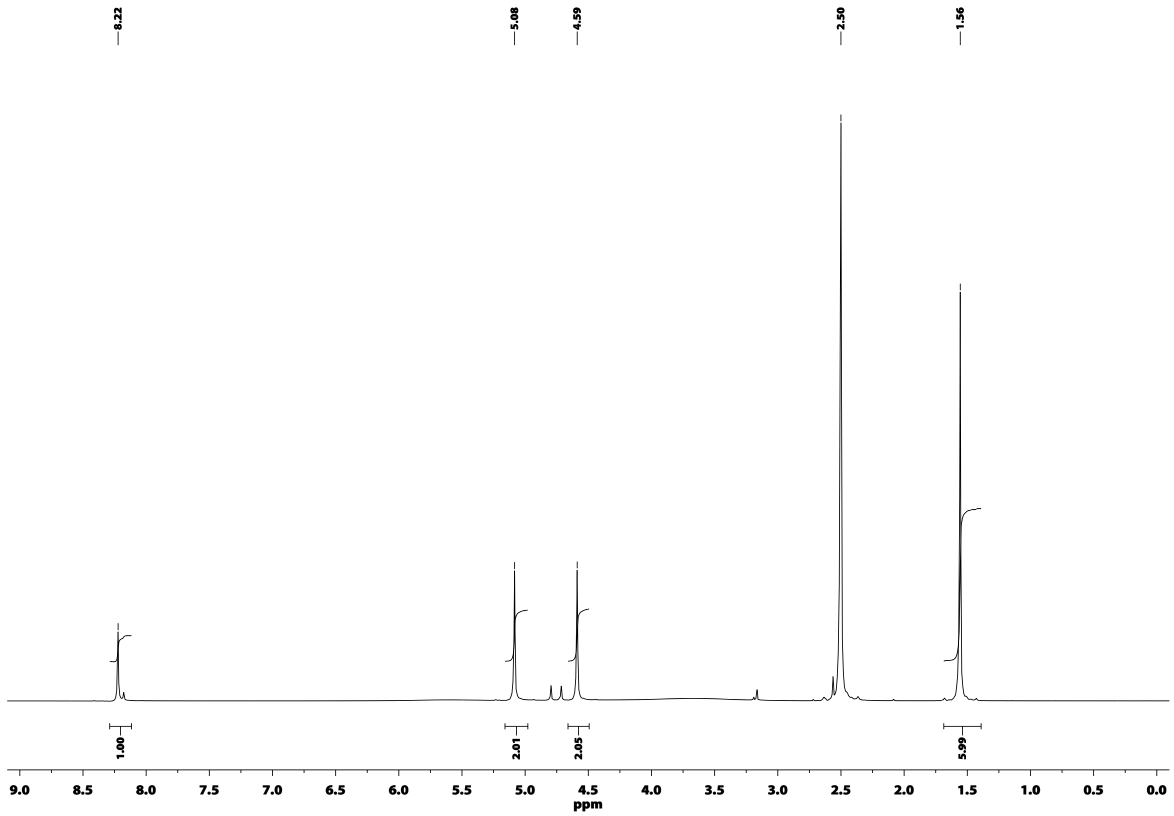

Figure S28. ^{1}H NMR spectra of the pyridinium salt **5** (500 MHz, DMSO-d_6, 298K).

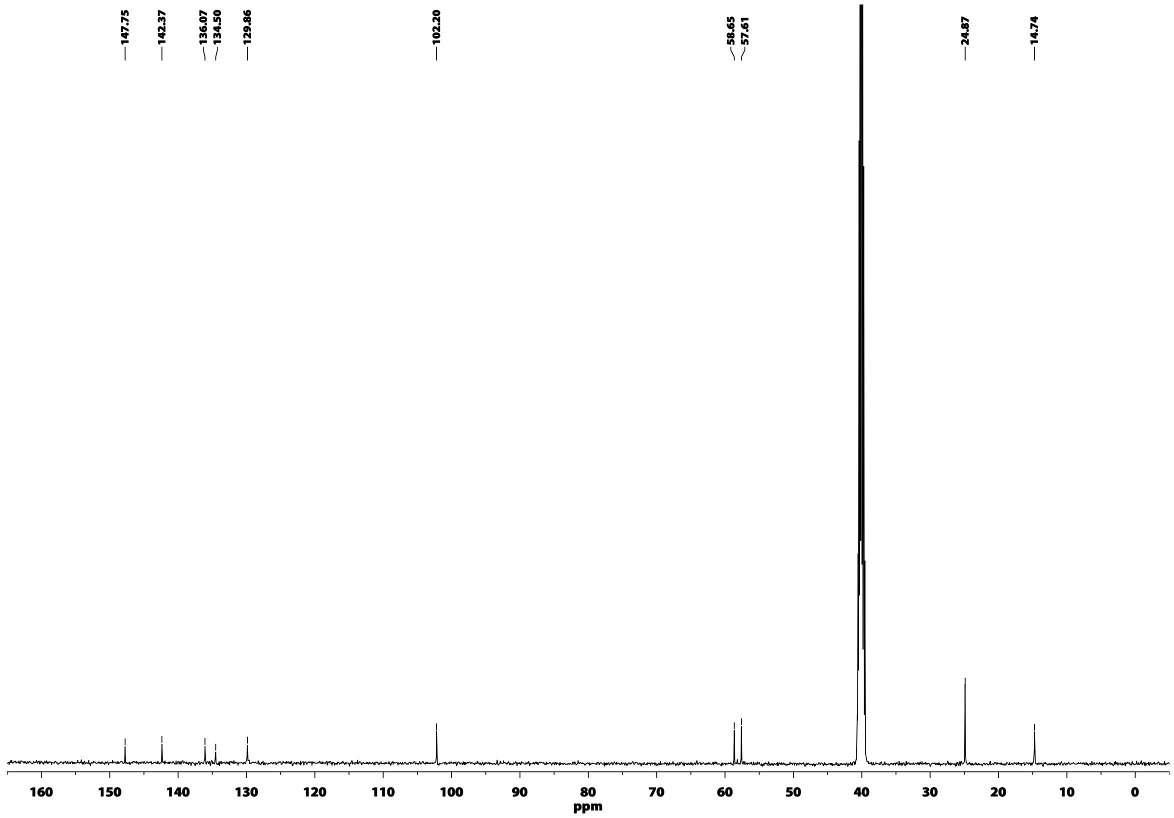

Figure S29. ^{13}C NMR spectra of the pyridinium salt **5** (125 MHz, DMSO-d_6, 298K).

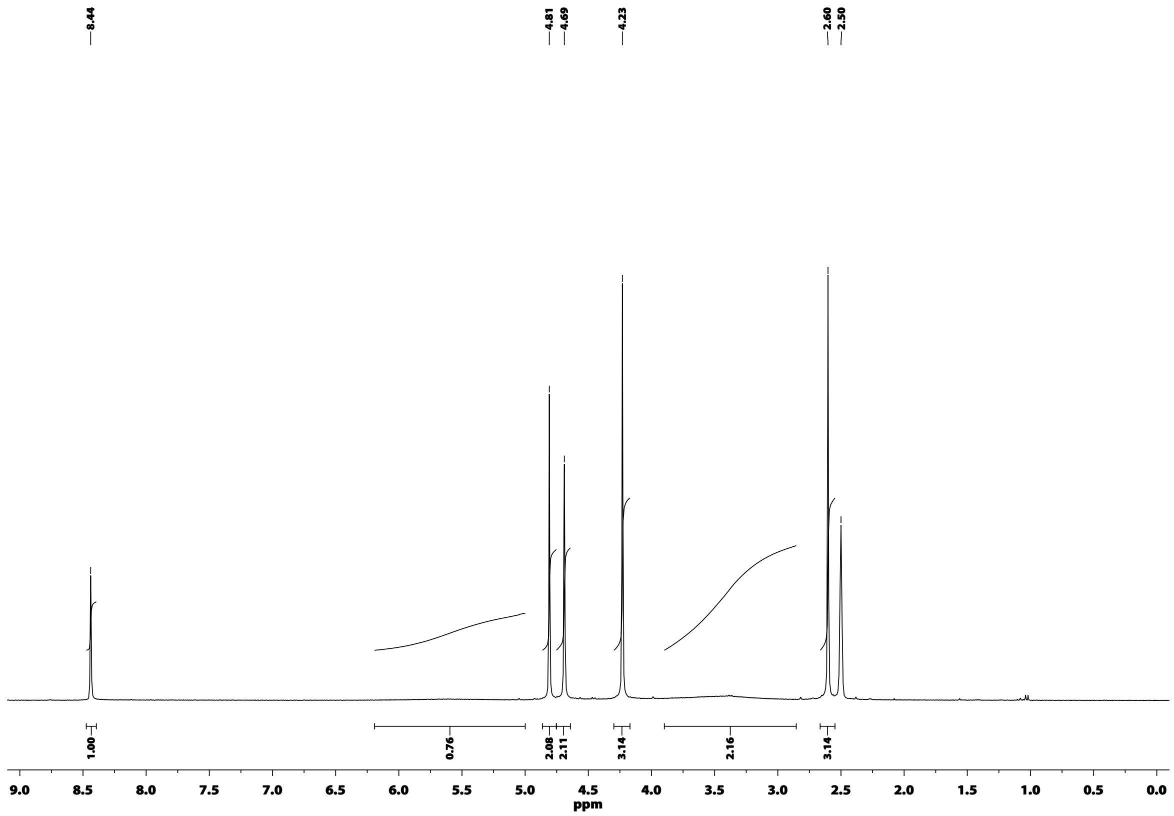

Figure S30. ^{1}H NMR spectra of the pyridinium salt **6** (300 MHz, DMSO-d_6, 298K).

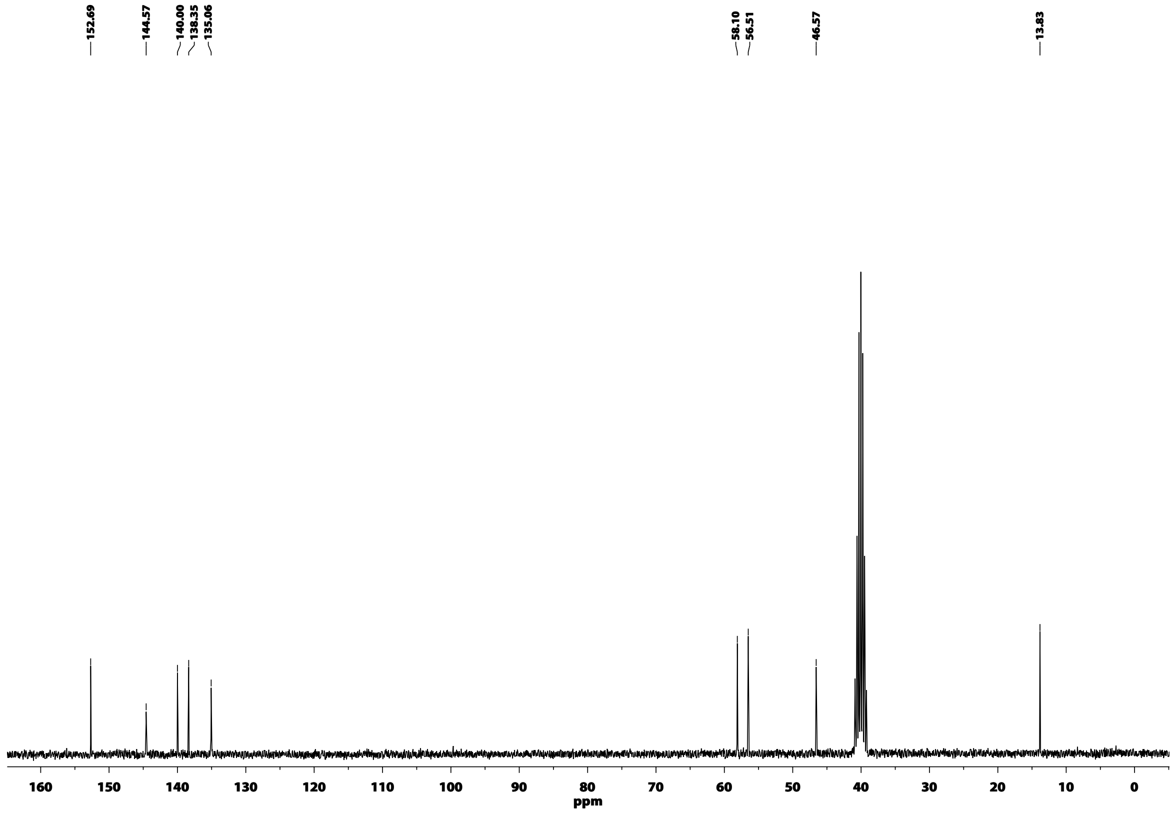

Figure S31. ^{13}C NMR spectra of the pyridinium salt **6** (75 MHz, DMSO-d_6, 298K).

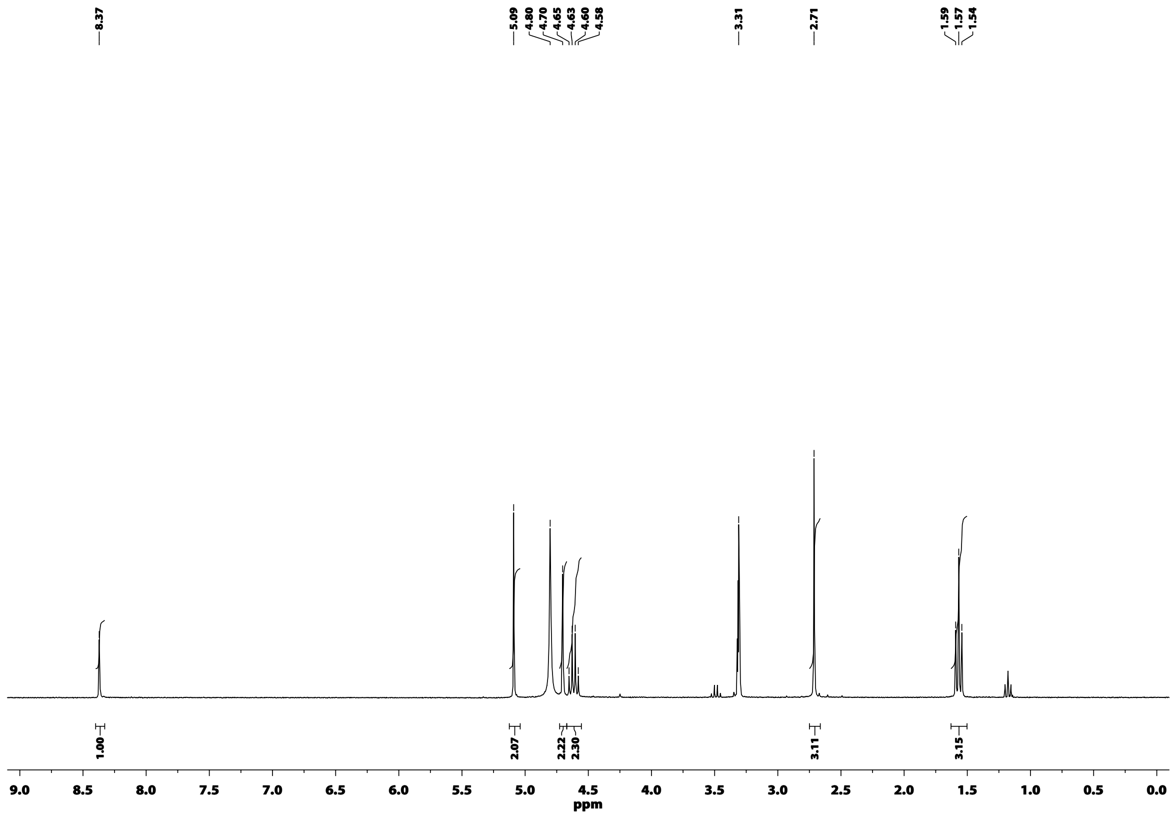

Figure S32. ^{1}H NMR spectra of the pyridinium salt **7** (300 MHz, MeOD-d_4, 298K).

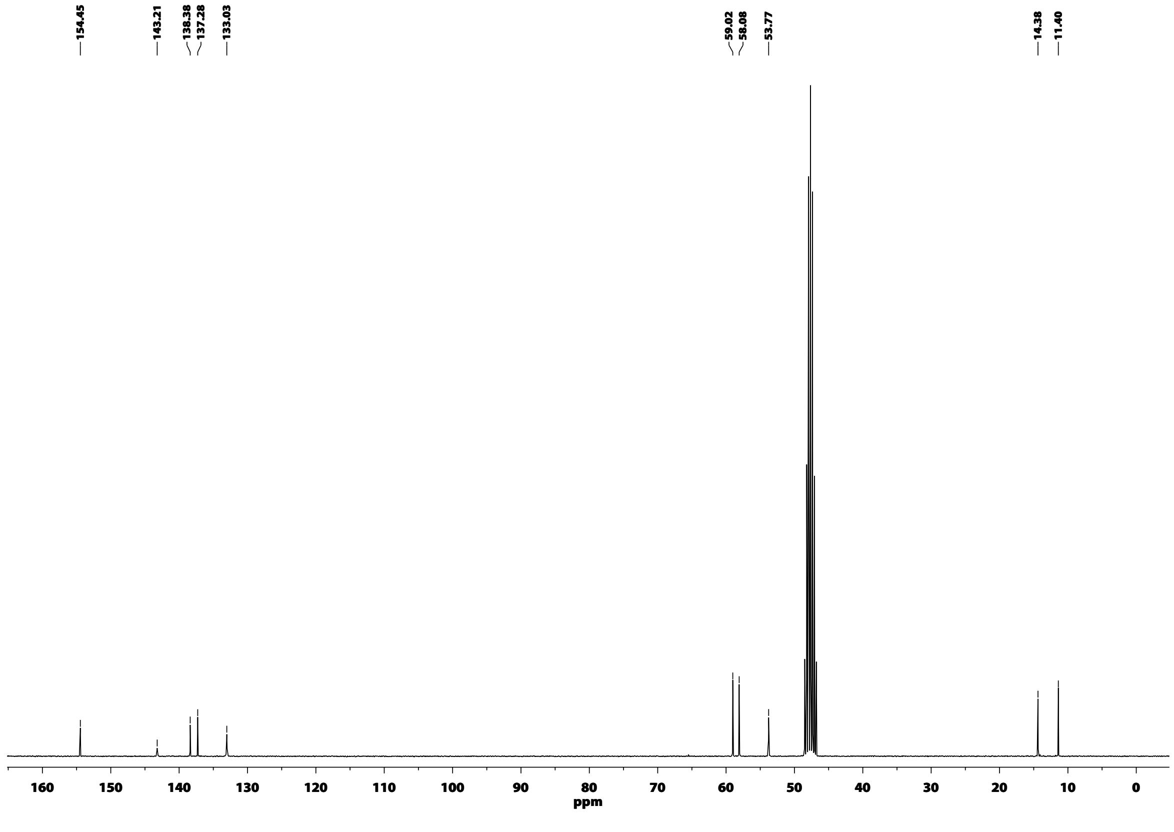

Figure S33. ^{13}C NMR spectra of the pyridinium salt **7** (75 MHz, MeOD-d_4, 298K).

3. References

[1] G. M. Sheldrick, *Acta Cryst A Foundations and Advances* **2015**, *71*, 3–8.
[2] G. M. Sheldrick, IUCr, *Acta Crystallogr C Struct Chem* **2015**, *71*, 3–8.
[3] O. V. Dolomanov, L. J. Bourhis, R. J. Gildea, J. A. K. Howard, H. Puschmann, *J Appl Crystallogr* **2009**, *42*, 339–341.
[4] F. Neese, *Wiley Interdiscip Rev Comput Mol Sci* **2022**, *12*, e1606.
[5] C. Adamo, V. Barone, *J Chem Phys* **1999**, *110*, 6158–6170.
[6] F. Weigend, R. Ahlrichs, *Physical Chemistry Chemical Physics* **2005**, *7*, 3297–3305.
[7] B. De Souza, G. Farias, F. Neese, R. Izsák, *J Chem Theory Comput* **2019**, *15*, 1896–1904.
[8] D. Peng, N. Middendorf, F. Weigend, M. Reiher, *Journal of Chemical Physics* **2013**, *138*, 184105.
[9] P. Pollak, F. Weigend, *J Chem Theory Comput* **2017**, *13*, 3696–3705.
[10] B. De Souza, F. Neese, R. Izsák, *Journal of Chemical Physics* **2018**, *148*, 034104.
[11] F. Neese, *J Comput Chem* **2003**, *24*, 1740–1747.
[12] F. Neese, F. Wennmohs, A. Hansen, U. Becker, *Chem Phys* **2009**, *356*, 98–109.
[13] B. Helmich-Paris, B. de Souza, F. Neese, R. Izsák, *Journal of Chemical Physics* **2021**, *155*, 104109.
[14] F. Neese, *J Comput Chem* **2023**, *44*, 381–396.
[15] B. Huang, R. Danso-Danquah, M. K. Safo, Y. Zhang, *SynOpen* **2020**, *4*, 51–54.
[16] J. Wang, X. Gu, H. Ma, Q. Peng, X. Huang, X. Zheng, S. H. P. Sung, G. Shan, J. W. Y. Lam, Z. Shuai, B. Z. Tang, *Nat Commun* **2018**, *9*, 1–9.
[17] J. Wang, X. Gu, P. Zhang, X. Huang, X. Zheng, M. Chen, H. Feng, R. T. K. Kwok, J. W. Y. Lam, B. Z. Tang, *J Am Chem Soc* **2017**, *139*, 16974–16979.
[18] A. Klimash, A. Prlj, D. S. Yufit, A. Mallick, B. F. E. Curchod, P. R. McGonigal, P. J. Skabara, M. K. Etherington, *J Mater Chem C Mater* **2022**, *10*, 9484–9491.
[19] X. Sun, B. Zhang, X. Li, C. O. Trindle, G. Zhang, *Journal of Physical Chemistry A* **2016**, *120*, 5791–5797.
[20] S. Garain, S. M. Wagalgave, A. A. Kongasseri, B. C. Garain, S. N. Ansari, G. Sardar, D. Kabra, S. K. Pati, S. J. George, *J Am Chem Soc* **2022**, *144*, 10854–10861.
[21] P. She, Y. Yu, Y. Qin, Y. Zhang, F. Li, Y. Ma, S. Liu, W. Huang, Q. Zhao, *Adv Opt Mater* **2020**, *8*, 1901437.
[22] J. H. Wei, J. Bin Luo, Z. L. He, Z. Z. Zhang, D. Bin Kuang, *Adv Opt Mater* **2023**, *11*, 2300328.
[23] Y. S. Wang, T. Zhao, J. H. Song, X. D. Tao, D. H. Zhang, L. Meng, X. L. Chen, C. Z. Lu, *Chemical Engineering Journal* **2023**, *460*, 141836.
[24] J. H. Wei, J. Bin Luo, Z. L. He, Q. P. Peng, J. H. Chen, Z. Z. Zhang, X. X. Guo, D. Bin Kuang, *Angewandte Chemie International Edition* **2024**, *63*, e202410514.
[25] G. Chen, H. Feng, F. Feng, P. Xu, J. Xu, S. Pan, Z. Qian, *Journal of Physical Chemistry Letters* **2018**, *9*, 6305–6311.
[26] G. Chen, S. Guo, H. Feng, Z. Qian, *J Mater Chem C Mater* **2019**, *7*, 14535–14542.
[27] I. Partanen, O. Al-Saedy, T. Eskelinen, A. J. Karttunen, J. J. Saarinen, O. Mrózek, A. Steffen, A. Belyaev, P. T. Chou, I. O. Koshevoy, *Angewandte Chemie International Edition* **2023**, *62*, e202305108.
[28] P. She, Y. Qin, Y. Ma, F. Li, J. Lu, P. Dai, H. Hu, X. Liu, S. Liu, W. Huang, Q. Zhao, *Sci China Mater* **2021**, *64*, 1485–1494.
